# Past foraminiferal acclimatization capacity is limited during future warming

**DOI:** 10.1038/s41586-024-08029-0

**Published:** 2024-11-13

**Authors:** Rui Ying, Fanny M. Monteiro, Jamie D. Wilson, Malin Ödalen, Daniela N. Schmidt

**Affiliations:** 1 School of Earth Sciences, University of Bristol, Bristol, UK; 2 School of Geographical Sciences, University of Bristol, Bristol, UK; 3 Department of Earth, Ocean and Ecological Sciences, University of Liverpool, Liverpool, UK; 4 GEOMAR Helmholtz Centre for Ocean Research Kiel, Kiel, Germany; 5 Department of Meteorology, Stockholm University, Stockholm, Sweden

**Keywords:** Marine biology, Climate and Earth system modelling, Climate-change ecology, Biogeography, Palaeoecology

## Abstract

Climate change affects marine organisms, causing migrations, biomass reduction and extinctions^1,2^. However, the abilities of marine species to adapt to these changes remain poorly constrained on both geological and anthropogenic timescales. Here we combine the fossil record and a global trait-based plankton model to study optimal temperatures of marine calcifying zooplankton (foraminifera, Rhizaria) through time. The results show that spinose foraminifera with algal symbionts acclimatized to deglacial warming at the end of the Last Glacial Maximum (LGM, 19–21 thousand years ago, ka), whereas foraminifera without symbionts (non-spinose or spinose) kept the same thermal preference and migrated polewards. However, when forcing the trait-based plankton model with rapid transient warming over the coming century (1.5 °C, 2 °C, 3 °C and 4 °C relative to pre-industrial baseline), the model suggests that the acclimatization capacities of all ecogroups are limited and insufficient to track warming rates. Therefore, foraminifera are projected to migrate polewards and reduce their global carbon biomass by 5.7–15.1% (depending on the warming) by 2100 relative to 1900–1950. Our study highlights the different challenges posed by anthropogenic and geological warming for marine plankton and their ecosystem functions.

## Abstract (web summary)

Data from the fossil record, together with computational modelling, are used to assess the response of foraminifera (marine zooplankton) to temperature changes through time and to predict how well they will adjust to future climate-change-induced ocean warming.

## Main

Climate change affects marine biodiversity and ecosystem function^1^. In response to warming, some marine organisms, such as fish, have shifted their habitat to track suitable temperatures^2–4^. In addition, some species have maintained or even increased performance in their local habitat through evolutionary adaptation or non-evolutionary acclimatization, both of which are commonly observed in marine microorganisms (plankton)^5–7^. However, the adaptive capacity of plankton and its limits remain poorly constrained in response to both past environmental changes and the ongoing climate crisis. This lack of knowledge causes uncertainty in estimating extinction risk^8^, distribution shifts^2,9^ and effects on the marine food web^10^ in response to a future warmer climate.

Planktic foraminifera are calcifying zooplankton and contribute roughly half of the modern global pelagic calcium carbonate production^11^. Their comprehensive microfossil records have been used to estimate the realized niche (the observed living conditions) of foraminifera through the late Quaternary glacial–interglacial climatic cycles^12,13^. Specifically, foraminifera were thought to have a limited potential to adaptively change ecological niches across time^12,13^. However, studies revealed that some foraminifera species could exhibit great plasticity in their optimal niche (the subset of the realized niche with the highest fitness)^13^, and extensive morphological, physiological and developmental plasticity that facilitates responses to modern^14^ and past^15^ environmental changes. This apparent disagreement between niche stability and plasticity leaves a key question open about the adaptive potential and vulnerability of the planktic ecosystem.

Here we aim to understand the adaptive capacity of foraminifera in response to environmental change at different rates and amplitudes by drawing on the extensive foraminiferal microfossil record and a novel trait-based model. We apply an Earth system model of intermediate complexity (cGENIE) to (a) the LGM (19–21 ka, around 6 °C cooler than the pre-industrial era); (b) the pre-industrial era (1765–1850); and (c) the next century (2100) under 1.5–4 °C warming scenarios relative to the pre-industrial baseline. The cGENIE Earth system model includes a trait-based mechanistic plankton model^16^ that recently incorporated the main foraminifera ecogroups, which are distinguished by the presence or absence of photosynthetic symbionts and the presence or absence of calcareous spines associated with grazing enhancement^17^. Any plankton in the model are allowed to grow anywhere, but the emerging biogeography is constrained by the local abiotic (temperature, nutrient and irradiance) and biotic factors (prey concentration, resource competition and grazing pressure) (Methods). This modelling principle mimics the process of natural selection and supports the plasticity of the plankton niche instead of specifying niche parameters^18^.

**Extended Data Fig. 1:**
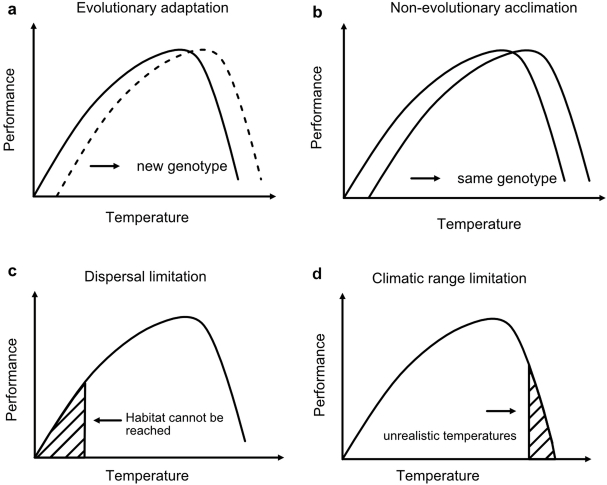
Schematic illustration of realized thermal niche change (the thermal performance function) under different causes. **a**–**d**, The adaptive responses including) evolution (defined by generation of new genotypes) (**a**) and acclimatization (non-evolutionary plasticity) (**b**) change the thermal performance function. By contrast, dispersal limitation where some species cannot reach a particular geographical area (hatched area) (**c**) or climatic range limitation where the temperature range is not available at a given time (e.g., temperatures above 25 °C during the LGM) (**d**) only affect the range of realized thermal niche.

The mechanistically simulated foraminiferal thermal performance curves (TPCs: abundance as a function of temperature) represent realized niches and are compared with estimates based on fossil abundance in well-dated marine sediments and temperature reconstructions for the LGM and pre-industrial era (Methods). On the basis of the TPCs, the optimal temperatures are quantified as the temperature range that leads to abundances exceeding half of the highest abundance, as shown in a previous report^19^ We regard a shift in optimal temperatures with warming as the signature of adaptive behaviour exemplified by higher growth rates or abundance as defined in previous experimental studies (Extended Data Fig. 1). However, because of the lack of absolute flux (accumulation rate) data in micropalaeontology studies, we use relative abundance here to determine the optimal condition of each species and the adaptive capacity of the whole assemblage. Such relative representations of foraminiferal optimal condition are consistent with experimental measurements^20^ and estimates based on maximal body size^21^ (Extended Data Table 1), confirming that they can act as a proxy of optimal condition.

## Foraminiferal niche in the LGM and pre-industrial era

**Fig. 1:**
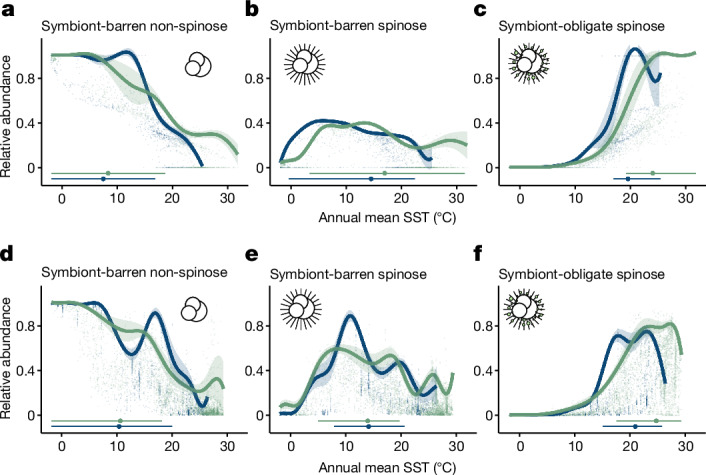
Reconstructed thermal performance of planktic foraminiferal ecogroups. The thermal performance of planktic foraminiferal ecogroups is reconstructed for the LGM (blue; 19–21 ka) and for the pre-industrial climate (green; 0 ka). **a**–**f**, Relative abundance of foraminifera ecogroups in the cGENIE model (**a**–**c**) and the fossil record (**d**–**f**), along with annual mean sea surface temperature (SST). Both model and observation show a stable thermal niche for symbiont-barren non-spinose foraminifera (**a**,**d**) and a shifted niche for symbiont-obligate spinose foraminifera (**c**,**f**) from the LGM to the pre-industrial age. The model–data mismatch (**b**,**e**) occurs for symbiont-barren spinose foraminifera probably because of the local adaptation of *G. bulloides* in this group^22^. All of the thermal performance curves or thermal niche envelopes (continuous lines) are estimated using an ensemble quantile regression model from 90th–99th levels and calculating the mean and s.d. (in the shading area). The raw data are plotted as shaded dots. The fossil sample size is for 1,433 for the LGM and 4,205 for the pre-industrial age. Optimal temperatures that exceed the 50% maximum abundance in both ages are labelled using horizontal bars (minimum to maximum), with the mean value shown as a dot. The foraminifera icon is for illustration and does not indicate a specific species. Source Data

**Extended Data Fig. 2:**
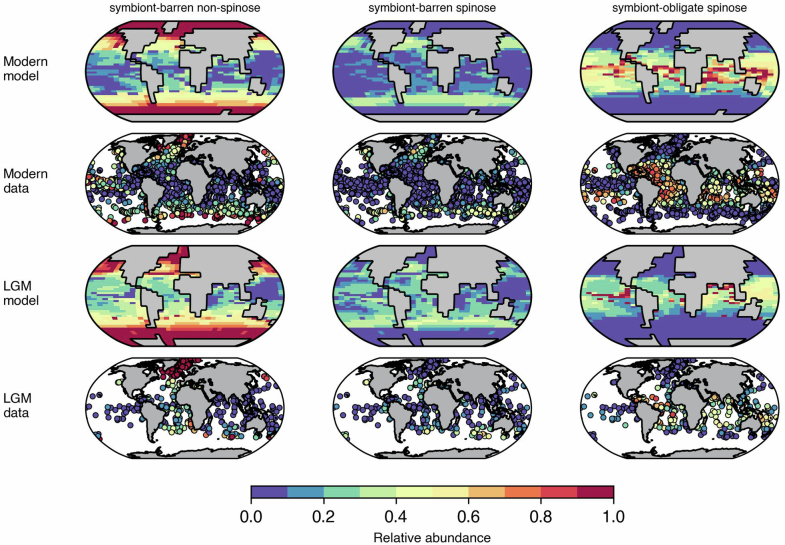
Model and fossil data comparison of relative abundance from sediment cores for the three foraminiferal ecogroups in the pre-industrial era and the LGM. The model captures both the observed habitat contraction of the symbiont-barren groups from LGM to pre-industrial with a broader geographic distribution in the LGM and the increasing relative abundance of the symbiont-obligate group in the modern ocean compared to the LGM. Source Data

In response to the environmental change from the LGM to the pre-industrial era, both the model and the data for foraminifera ecogroups show diverse responses. The symbiont-barren (that is, no symbiont) non-spinose foraminifera retained their optimal temperature at 8/10 °C (model/observation) during the deglacial transition (Fig. 1a,d). This niche stability caused a contraction of geographical range into the high latitudes from the glacial to the pre-industrial (Extended Data Fig. 2). The symbiont-barren spinose foraminifera are an opportunistic (high-food) group dominated by the species *Globigerina bulloides*. The model suggests a wider optimal habitat than the data, with a mean value shifting from 15 °C to 17 °C in comparison with the retained 14 °C in the data (Fig. 1b,e). The model–data mismatch highlights the difficulty of identifying the optimal conditions for cryptic species with multiple genotypes in *G. bulloides* and their specific thermal sensitivities^22^ with one ecological setting in the model. Specifically, the model cannot differentiate between the open ocean types found in temperate waters and the upwelling types associated with the coastal high-nutrient settings. Finally, for symbiont-obligate spinose foraminifera that occupy the shallow and warm open oceans in the mid-to-low latitudes, the modelled and observed optimal temperature both increased from 20/21 °C (LGM model/observation) to 24/25 °C (pre-industrial model/observation) (Fig. 1c,f and Extended Data Fig. 2).

**Extended Data Fig. 3:**
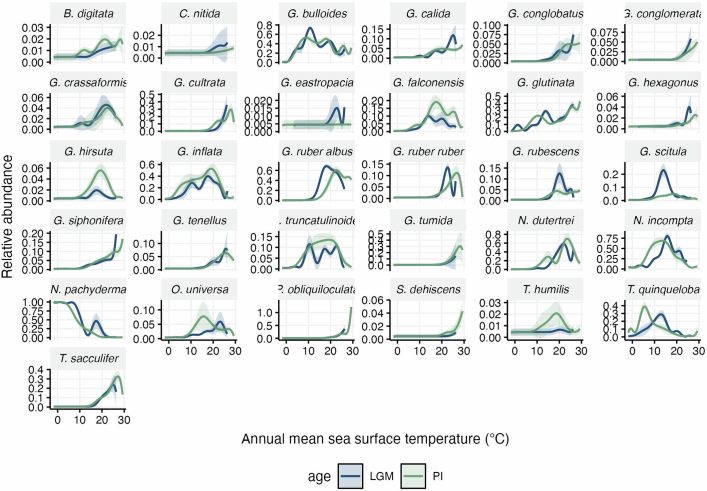
Species-level thermal performance curves for the LGM and the pre-industrial age. Data as in Fig. 1 (LGM (19–21 ka), blue; pre-industrial age (0 ka), green) but here disaggregated at species level. The full species name and related ecogroup can be found in Supplementary Table 1. Notable species showing changes in the thermal performance interpreted as acclimatization include the tropical symbiont-bearing taxa *T. sacculifer*, *G. ruber ruber*, *G. ruber albus*, and *N. dutertrei* and *N. incompta*. Note: the y scale is set different for each species to highlight patterns. The shaded area represents the standard deviation of the 90–99th quantile regression model results.

The fossil data allow us to further investigate species-leveratiol responses. We reconstructed the TPCs of 31 foraminifera species on the basis of LGM and pre-industrial fossil observations, extending our analysis to species that do not belong to the above ecogroups (Extended Data Fig. 3 and Extended Data Table 1). Our species-based results agree with and expand previous findings about species-dependent thermal niches^13^. Although some species exhibited niche stability (*G. bulloides* and *Neogloboquadrina pachyderma*), others shifted their optimal niche towards colder (*Orbulina universa*, *Neogloboquadrina incompta* and *Turborotalita quinqueloba*) or warmer (*Globigerinoides ruber albus*, *Globigerinoides ruber ruber*, *Trilobatus sacculifer*, *Neogloboquadrina dutertrei* and *Pulleniatina obliquiloculata*) environments. However, in this species-level analysis, changes in thermal optima cannot be explained by ecological traits such as symbiosis or spines (two-way ANOVA, symbiosis: *F*_4,26_ = 1.248, *P* = 0.321; spine: *F*_1,26_ = 0.228, *P* = 0.638). The discrepancy between species- and ecogroup-level analysis suggests that our ecogroup-level model captures the response of dominant foraminifera but ignores the interspecies ecological differences of rare taxa.

**Extended Data Fig. 4:**
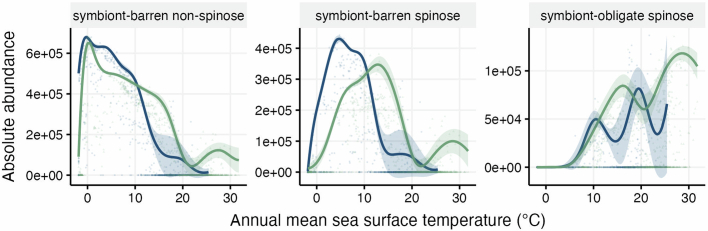
Modelled foraminiferal thermal performance curve based on the absolute abundance of cGENIE output. The consistency between absolute abundance and relative abundance-based results (Fig. 1) indicates that niche shift was not caused by community compositional change only.

**Extended Data Fig. 5:**
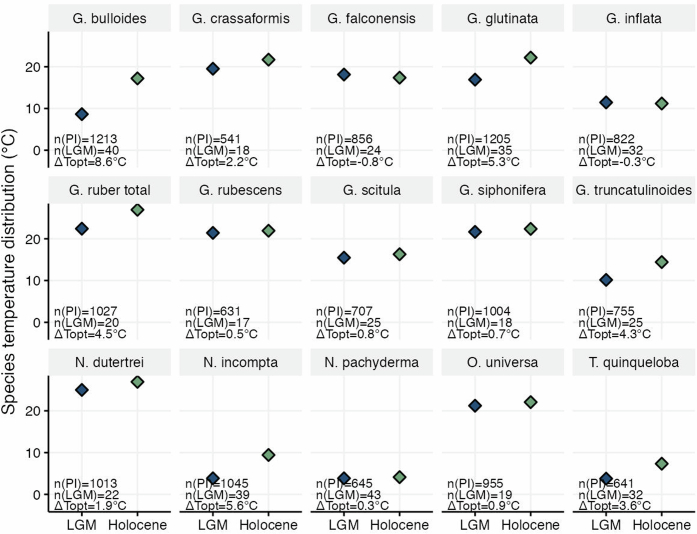
Foraminiferal thermal optimal niche in the LGM and Holocene using presence- and absence-based foraminifera occurrences and vertically resolved temperature data. The optimal temperature of the species, defined as the highest occurrence probability, is marked by diamond for both LGM (20 ka bin, left) and Holocene (4 ka bin, right). The annotated text shows the optimal temperature change (Holocene- LGM) for each species and the number of presence data to reconstruct the optimal niche. The vertically resolved temperature data are from a previous study^12^.

The shift in the TPC of symbiotic foraminifera provides evidence of their adaptive abilities to warming. Although the TPCs are based on relative abundance data, our results cannot be explained by species replacement alone, because modelled (Extended Data Fig. 4) and observed absolute abundance (foraminifera accumulation rates) have increased since the LGM^23^, reflecting their unambiguously increased fitness under warming. Similarly, these results are not caused by dispersal limitation or by a lack of warm habitats in the LGM, because these processes modify the boundary rather than the shape of the TPC (Extended Data Fig. 1). Finally, we have reanalysed niche reconstruction data from a previous study^12^, which shows a similarly increasing optimal temperature from the LGM to the pre-industrial era (−0.3 °C to 8.6 °C) (Extended Data Fig. 5 and Supplementary Methods). This confirms the robustness of our results, because the same response is detected independent of evidence used: relative abundance, presence–absence, accumulation rate or model simulation.

We interpret the adaptive response of symbiont-obligate foraminifera shown in the data and our model during the deglacial warming as acclimatization, which we define as the non-evolutionary reversible response of a species to maintain or increase performance^5,24,25^. This interpretation of acclimatization is based on the knowledge that no novel species or trait emerged across the planktonic foraminifera taxa since the LGM (that is, no evolutionary adaptation). Previous studies^26,27^ show that the most recent origination amongst modern morphologically defined foraminifera species occurred diachronously from 2.2–32.2 million years ago, hence much earlier than the LGM (19–21 ka). Although there is evidence for genetic changes within the foraminiferal morphotypes (termed as ‘cryptic species’), the most recent genetic split occurred during marine isotope stage 5.5 (roughly 120 ka)^28^—again, earlier than the LGM. Moreover, the symbiont-obligate foraminifera ecogroup, which presents the optimal niche change during the deglaciation, has the least cryptic species in the community^29^. Furthermore, the dominant species (*T. sacculifer*) in this ecogroup has no cryptic species. This reinforces that no genetic divergence occurred since the LGM to support a possible interpretation of evolutionary change of foraminifera species.

## Projection of foraminifera by 2100

**Fig. 2:**
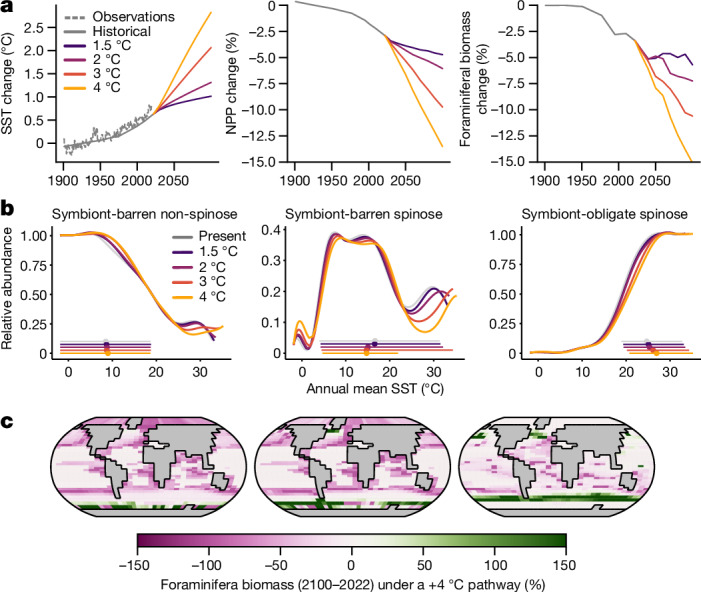
Response of plankton ecosystems to future warming in cGENIE. **a**, Modelled change in SST, NPP and globally integrated foraminifera carbon biomass when global mean surface temperature increases by 1.5 °C, 2 °C, 3 °C and 4 °C in 2100 relative to the 1900–1950 average. We used historical CO_2_ to force the model (with comparison with ERSST v.5 (ref. ^48^) SST observations), and linear CO_2_ forcings to mimic future warming. **b**, Thermal performance curves of the three foraminifera ecogroups in 2100 as estimated in Fig. 1. The grey curves show the present niches. **c**, Carbon biomass future trend for each foraminifera group in response to a 4 °C warming by 2100 relative to the present. All biomass in this figure refers to biomass standing stock, but the trend is the same for biomass production rate (Supplementary Fig. 7).

Given the thermal response identified in the past, it is possible to consider whether acclimatization to future warming will allow foraminifera to maintain their biomass and ecosystem functions. To answer this question, we conducted a series of transient simulations from a pre-industrial climate to 2100 using the same model used for the last deglacial warming experiment. We used historical CO_2_ concentrations to force the model from pre-industrial to the present day (2022), and four idealized linear CO_2_ forcings to simulate future warming scenarios (1.5 °C, 2 °C, 3 °C and 4 °C by 2100 relative to the 1900–1950 average; Fig. 2a and Supplementary Fig. 2). By reproducing the observation that the global mean sea surface temperature (SST) in the present day (2022) is around 0.6 °C warmer than the 1900–1950 average (Fig. 2a), the model demonstrates its ability to simulate future scenarios in terms of the rate of warming. By 2100, the global SST will increase by 1.0 °C, 1.3 °C, 2.1 °C and 2.8 °C in response to the different warming forcings. The resulting ocean net primary productivity (NPP) drops by 4.7%, 6.1%, 9.7% and 13.5%, respectively (Fig. 2a), in good agreement with the Coupled Model Intercomparison Project (CMIP5–CMIP6) range (Supplementary Fig. 3).

The modelled foraminifera show limited thermal adaptive potentials in the future (Fig. 2b). The mean thermal optima of symbiont-barren non-spinose foraminifera only shift by 0.0 °C, 0.2 °C, 0.2 °C and 0.5 °C compared to the present day (2022) under our four warming scenarios. The mean optimal temperature for symbiont-barren spinose foraminifera decreases by 1.7 °C, 1.5 °C, 1.2 °C and 0.6 °C as their tropical habitats vanish by 2100. Symbiont-obligate foraminifera are projected to have a greater acclimatization, with 0.4 °C, 0.7 °C, 1.5 °C and 2.3 °C shifts in optima temperature, comparable with the response to deglacial warming (Fig. 1). However, the reduction in the absolute abundance of symbiont-obligate foraminifera indicates that optimal temperature changes are more limited than it seems (0.4 °C, 0.7 °C, 1.2 °C and 1.8 °C on the basis of absolute abundance). Our simulations indicate that, even when the possibility of acclimatization is accounted for in the model, planktonic foraminifera will not be able to track the pace of future warming.

As observed since the pre-industrial age^30,31^, planktic foraminifera in the mid-to-high latitudes will migrate polewards in the future. Symbiont-barren spinose foraminifera, such as *G. bulloides*, will increase their biomass standing stocks (hereafter, biomass) in the Southern Ocean and the North Atlantic, benefitting from niche expansion (Fig. 2c) into a habitat in which symbiont-barren non-spinose species dominate today^32^. The biomass of warm-adapted symbiont-obligate spinose will increase in the North subpolar regions and subantarctic zones (Fig. 2c), which agrees with the observations in the Arctic^33^ and Southern Ocean^34^.

**Fig. 3:**
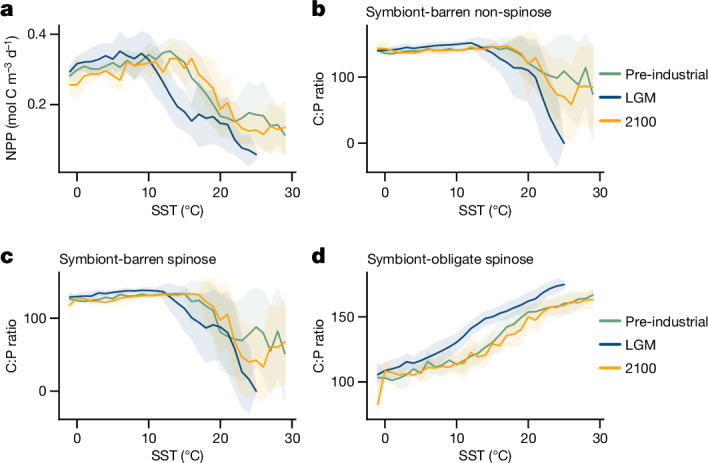
Ecological and physiological drivers of change in foraminiferal thermal performance. **a**, NPP (line, mean; shaded area, s.d.) for each temperature bin (1 °C). **b**–**d**, The cellular C:P biomass ratio of foraminifera ecogroups (symbiont-barren non-spinose (**b**), symbiont-barren spinose (**c**) and symbiont-obligate spinose (**d**)) determines the assimilation efficiency of prey. A clear change in the NPP–temperature relationship occurred from the LGM to the pre-industrial era but is not seen in the future under a 4 °C warming scenario, explaining the different responses between the two warming events. The distinct C:P shift indicates the physiological adjustment of symbiont-obligate spinose foraminifera under different trophic conditions. Source Data

**Extended Data Fig. 6:**
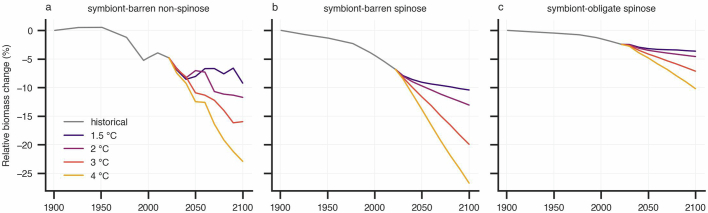
Projected globally integrated carbon biomass (standing stock) of each foraminifera ecogroup from 2022 to 2100 in response to four different warming scenarios. **a**,**b**, The biomasses of symbiont-barren non-spinose (**a**) and symbiont-barren spinose (**b**) foraminifera show a reduction of 10–27% relative to the 1900–1950 average. **c**, By contrast, symbiont-obligate foraminifera are the most resilient to warming with 4–10% reduction depending on the warming pathways. Note that symbiont-barren foraminifera drive 77% of the total foraminifera change.

Overall, the global biomass of foraminifera is predicted to decline in the future (Fig. 2a,c and Extended Data Fig. 6). The model estimates that global foraminifera biomass has already decreased by 3.4% at present (2022) relative to the 1900–1950 average (Fig. 3a). With a warming of 1.5, 2, 3, and 4 °C by 2100, foraminifera biomass is projected to reduce further by 5.7, 7.2, 10.6 and 15.1%, respectively (Fig. 2a). This biomass loss is widespread across the ocean, except in the Southern Ocean and to a lower extent in the North subpolar regions, where species replacement occurs (Fig. 2c). The loss of biomass is uneven across ecogroups and is driven mainly by the two symbiont-barren groups (9–23% and 10–27% for non-spinose and spinose, respectively), which account for around 77% of the total change (Extended Data Fig. 6). We suggest that this preferential loss is caused by the reliance of these groups on phytoplankton as prey, which are also decreasing in biomass (Fig. 2a). By contrast, symbiotic foraminifera show lower losses (4–10% biomass loss; Extended Data Fig. 6), because they can draw on multiple energy pathways, highlighting the importance of ecological redundancy to reduce overall losses in biomass. Our model results are well supported by census counts of planktic foraminifers spanning the past century^31^. It should be noted that our model does not include the risk of symbiont bleaching; this has been suggested to affect foraminiferal physiology in past warm events^35^, is evident today in coral symbionts^36^ and would increase the vulnerability of the group.

## Role of food in acclimatization

The foraminiferal response in the past and future allows us to examine the mechanism of acclimatization. To understand the details, we disentangled the ecophysiological processes that account for the plankton performance. The modelled plankton growth rate monotonically increases with temperature and is modulated by nutrient availability^37^. The top-down control (grazing pressure) is typically negligible for foraminifera, owing to their small biomass^38^. The modelled biomass loss, including the mortality and respiration rate, has the same thermal sensitivity across different climates and seems not to contribute to the observed change in thermal niche (Supplementary Methods). Therefore, the modelled thermal acclimatization is likely to be driven by changes in prey availability (grazing source) and prey quality (assimilation efficiency), and by autotrophic input from symbionts that support the metabolic needs for living at higher temperatures.

We investigate the role of food by comparing the ocean’s primary production and temperature for the LGM, pre-industrial era and future in response to the +4 °C scenario. The NPP–SST relationship is similar across the time slices at high latitudes (lower than 10 °C), but different at low latitudes (higher than 10 °C) (Fig. 3a). This difference explains the stable niche of the symbiont-barren non-spinose ecogroup, which is heterotrophic and tracks the footprint of the primary producers (Figs. 1 and 3a–c). For a more detailed understanding, we analysed the foraminiferal cellular physiology (variable stoichiometry)^39^ to determine the prey assimilation efficiency. We found a nearly linear increase in the carbon to phosphate (C:P) biomass ratio of symbiont-obligate foraminifera with temperature (Fig. 3d), indicating a more efficient use of nutrients in warm oligotrophic environments. The C:P ratio decreases because more prey (indicated by NPP) can be found in warmer environments during the pre-industrial era, compared with the LGM (Fig. 3a). This supports the hypothesis that symbiont-obligate foraminifera benefit from symbiosis to supplement metabolic needs, whereas symbiont-barren foraminifera depend on food availability, as observed in culture studies in which algal symbionts transfer carbon-enriched polysaccharide (for example, starch) through lipid droplets to the host cytoplasm^40^. This energy transfer allows the host with a high affinity to nutrients to counteract nutrient scarcity. Our interpretation is further supported by the observation that the foraminiferal nutrient content (size-normalized protein) is determined by NPP and chlorophyll *a* concentrations^41^.

**Extended Data Fig. 7:**
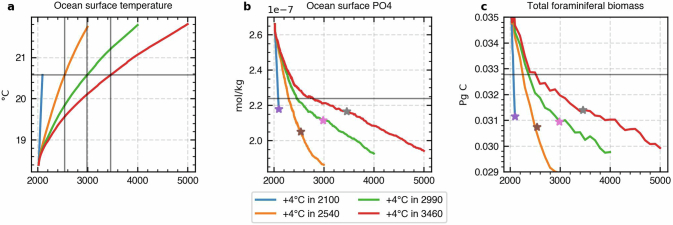
Model responses of surface mean nutrient (shown here as PO_4_) and total foraminifera biomass to a 4 °C warming under different warming rates. **a**, The ocean surface temperature responses to CO_2_ emission at different rates. The grey lines mark the model years with the same warming magnitudes (4 °C warming relative to the 1900–1950 average) for different model runs. **b**, The surface PO_4_ concentration changes in response to warmings. **c**, The total foraminiferal biomass in each model experiments. The stars in **b**,**c** represent model years when the ocean surface reaches the same warming magnitude at different rates. The horizontal lines in subplots **b**,**c** show the state in surface nutrient and foraminifera biomass under fully equilibrium state. This figure shows that decreasing the warming rate damps any resulting ocean stratification and associated reduction of surface nutrient supply.

**Extended Data Fig. 8:**
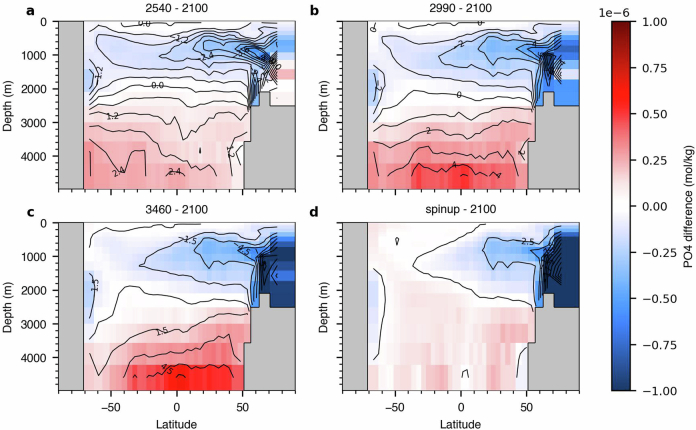
The global zonal mean PO_4_ anomaly in the slow-warming scenarios relative to the fastest warming scenario. **a**–**d**, As in Extended Data Fig. 7, each panel shows the nutrient distribution difference at different warming rate despite the same warming magnitude. Compared to the fastest warming scenario (reached in 2100), the slower warming rates (reached in 2540, 2990, 3460, and in a steady-state model) allow more nutrients to be delivered to the surface.

**Extended Data Fig. 9:**
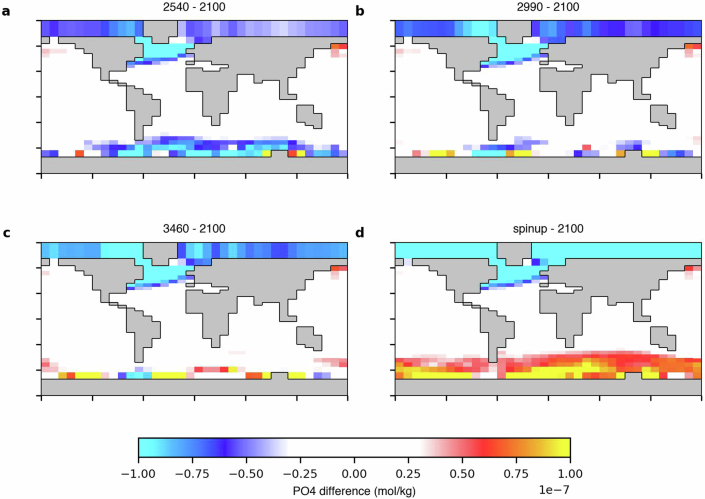
The spatial distribution of surface PO_4_ concentration difference between slow and fast warming experiments. As shown in the Extended Data Fig. 8, with a gradually slower emission rate (from **a** to **d**), there is more nutrient availability in the Southern Ocean under the same surface ocean warming level. The North Atlantic, however, keeps being isolated and nutrient-depleted.

The different pattern of NPP–SST for future scenarios compared with the LGM corroborates the distinction between past and ongoing warming (Fig. 3a). The deglacial warming was associated with active ocean mixing and a higher nutrient supply^42,43^, whereas the modern warming is characterized by increasing stratification. The abrupt current warming determines the climatic impacts on the ocean circulation, ice sheets, wind stress and nutrient supply, which are distinctly different from the LGM changes. To validate the importance of this, we forced the model to reach the same warming magnitude but at different rates. All experiments show ocean stratification and reduced nutrient delivery to the surface (Extended Data Fig. 7). However, in response to slower warming scenarios, ocean circulation can mitigate the stratification and allow a greater amount of nutrients to be supplied to the upper layers, especially in the Southern Ocean (Extended Data Figs. 8 and 9).

## Implications

Marine plankton support the flux of energy and material through the marine food web and the storage of carbon in the ocean. Consequently, their adaptive capacity directly influences fishery production and the global carbon cycle. Our study provides several lines of evidence for acclimatization in marine calcifying zooplankton on various geological timescales, and suggests that this acclimatization depends on ecology and food supply. The difference in response to past, present and future warming highlights the unprecedented risks for the marine plankton ecosystem, which could be further exacerbated by ocean acidification^44^, symbiont bleaching^35^, deoxygenation^45^ and other potentially synergistic stressors. The importance of the rate of change in determining the capacity of foraminifera acclimatization agrees with a previous modelling study^46^, which came to similar conclusions about the phytoplankton’s adaptive responses. However, to fundamentally understand these risks, assessments of plankton life cycles are needed to work out how changing environments select phenotypes in the offspring population^47^ and influence their ontogeny. Overall, the acceleration of present climate change is challenging the adaptive capacity of marine plankton and their ecosystem functioning.

## Methods

### cGENIE Earth system model

cGENIE is an Earth system model of intermediate complexity, with a 36 × 36 equal-area grid (10° longitude and uniform in the sine of latitude) and 16 vertical ocean layers, that resolves multiple biogeochemical cycles. It facilitates the exploration of long-term climate, marine ecology and carbon cycling, particularly in palaeoceanography studies^49^. It couples several components, including a two-dimensional energy–moisture balance (EMBM) atmosphere and a thermodynamic sea-ice model^50^, a three-dimensional ocean circulation (C-GOLDSTEIN)^51^ combined with ocean biogeochemistry (BIOGEM)^52^ and a trait-based plankton community model (EcoGENIE)^16^. The EcoGENIE model includes a full spectrum of planktic foraminifera ecogroups^17,32^ on the basis of the implementation of their functional traits (body size, calcification, symbionts, spines and feeding behaviour). The foraminifera biomass is determined by environmental temperature, prey availability and biotic interaction with other plankton groups. The foraminifera parameterizations have been improved in this study (Supplementary Fig. 5 and Supplementary Table 2). Concrete details can be found in the Supplementary Methods.

#### LGM, pre-industrial and future model simulations

We derive the palaeogeographical configuration, zonal albedo profile and ice-sheet data from the HadCM3 model^53^ to configure the cGENIE LGM model. We apply the LGM climate boundary conditions including lower atmospheric CO_2_ (193 ppm), a new dust deposition field^54^, enhanced wind stress^55^ and orbital parameters following the PMIP4 protocol^56^. In addition, we apply a brine rejection relocation in the Southern Ocean, which redistributes brine (salt expelled during sea-ice production) from the surface to the deep ocean, following a previous report^57^ and based on another study^58^ The parameterizations were constrained by a global compilation of marine carbon stable isotope (δ^13^C) data^59^. The simulated LGM ocean shows a weaker and shallower Atlantic Meridional Overturning Circulation (AMOC) than the modern one^57^, agreeing with previous modelling results^60^.

On the basis of these glacial boundary conditions, we spin up the model for 10,000 years to reach a steady state. The model predicts a regionally enhanced carbon export in the LGM compared to the pre-industrial era, as suggested by a multiple-proxy compilation (Supplementary Fig. 1). The LGM SST in cGENIE is around 5 °C cooler than the pre-industrial one, which is overall higher than the data for the LGM, but in agreement with some PMIP4 models^61^. We note that the LGM SST reconstruction uncertainty is still an unsolved question^62^, which can be attributed both to models^61^ and proxies^63^.

The pre-industrial experiment adopts a similar grid and carbon-cycle configuration to that described previously^64^. The model is spun up under the pre-industrial state (1765) for 10,000 years, with an atmospheric CO_2_ concentration of 278 ppm and a dust field from a previous report^65^. We next use the historical CO_2_ data as input to force the model to run from the pre-industrial age to the present day (2022)^48^, and impose a series of idealized future CO_2_ forcings causing 1.5 °C, 2 °C, 3 °C and 4 °C global air warming, with all the other parameterization the same as for the pre-industrial simulation (Supplementary Fig. 2). For simplicity, we do not include any other greenhouse gases in this study.

The historical global mean surface temperature aligns well with the HadCRUT5 dataset^66^, and the global mean SST agrees with the ERSST v.5 dataset^48^ (Supplementary Fig. 2). The future experiment results are comparable with fully coupled CMIP Earth system models. The model predicts the same subtropical and tropical zooplankton biomass loss and polar biomass increment as CMIP6 models^67^. The NPP is projected to decline between 1% (1 °C) and 10% (4 °C) by 2100 relative to the present day, like the CMIP5 average and the lower bound of CMIP6 (ref. ^67^) (Supplementary Fig. 3).

#### Foraminiferal biomass to abundance

All of the modelled foraminifera carbon biomass is converted to absolute abundance to determine relative abundance. The conversion follows the equation below, in which biomass and cell volume are taken from the model, and the carbon biomass density uses a foraminifera-average value (0.089 pg C μm^−3^) derived from refs. ^68,69^.

$${\rm{Abundance}}={\rm{Biomass}}/({\rm{Cell}}\;{\rm{volume}}\times {\rm{Density}})$$

### LGM and pre-industrial observational data

#### LGM and pre-industrial planktic foraminifera abundance data

We use the curated sediment foraminifera assemblage datasets ForCenS^70^ and MARGO^71^ to represent the pre-industrial and the LGM abundance. The MARGO samples have undergone a quality level assessment based on the age control and have no bias caused by calcite dissolution^71^. Both datasets have global coverage, with a bias towards the low latitudes (Supplementary Fig. 4). We only use relative abundance data and convert absolute count to relative abundance if necessary. We keep the different sample depths in the same sediment core (that is, no averaging) to include the uncertainty within each time interval. After the data standardization (see below), there are 4,205 data points for the pre-industrial age (41 species) and 1,433 data points for the LGM (35 species).

For consistency, we use the latest taxonomic standardization^72^ in both datasets. Specifically, we merged *Globorotalia truncatulinoides* sinistral and *G. truncatulinoides* dextral into *G. truncatulinoides*, and *T. sacculifer* with sac and without sac chamber (*Trilobatus trilobus*) into *T. sacculifer*. We separate *G. ruber* into *G. ruber albus* (white) and *G. ruber ruber* (pink) and use *N. pachyderma* and *N. incompta* to replace the *N. pachyderma* sinistral and *N. pachyderma* dextral. The commonly used ‘P/D intergrades’ is merged into *N. incompta* following the ForCenS dataset^70^. We adopt the use of *Globorotalia cultrata* and *Globorotalia eastropacia to* replace *Globorotalia menardii* and *Globorotalia theyeri*, respectively.

These species-based data are aggregated into functional groups according to their trait of spines and algal symbionts (Supplementary Table 1). The algal symbiont information follows a previous report^73^: ‘symbiont-barren’ (no symbiont), ‘symbiont-obligate’ (must live with symbiont), ‘symbiont-facultative’ (has been found with and without symbionts), ‘symbiont-bearing’ (newly detected relationship in ref. ^73^) and ‘undetermined’. The spine information is based on a previous report^74^ and mikrotax^75^ (https://www.mikrotax.org), with the classification of ‘spinose’, ‘non-spinose’ and ‘undetermined’. We report only three groups: ‘symbiont-barren non-spinose’, ‘symbiont-barren spinose’ and ‘symbiont-obligate spinose’, owing to limited biological understanding of the drivers of symbiont-facultative behaviour and its benefits or trade-offs. However, the species-level data are reported as completely as possible for the readers’ interest.

#### LGM and pre-industrial SST data

We use the geographical information in the abundance dataset to look up the SST in the nearest grid location within data products. We use the HadISST1 dataset^76^ (1 × 1°, latitude × longitude, 1870–1900 annual mean climatology) as our pre-industrial temperature reference and a previous data assimilation (Tierney et al.^77^) (1.9 × 2.5°, latitude × longitude) as our LGM reference. Tierney et al.^77^ used an Ensemble Kalman filter to incorporate the information of geochemical proxy data compilation (19–23 ka) with the constraints of a climate model (iCESM). The proxy compilation includes organic chemistry-based proxies (${U}_{37}^{{{\rm{K}}}^{{\prime} }}$, TEX_86_), and foraminifera-shell-based δ^18^O and Mg/Ca. Each type of proxy was calibrated using a Bayesian model to propagate proxy uncertainties and seasonal bias. We do not use assemblage-based temperature reconstruction from the MARGO to avoid circular reasoning.

It is worth noting that the temperature data used here only represent the surface layer (0.5 m for LGM data and 0.2 m for HadISST) and its long-term climatology, therefore not indicating the in situ temperature of the precise habitat. The common and accepted use of annual mean SST averages over the seasonal variability does not reflect the dynamic vertical distribution of foraminifera. These limitations do not affect our inference of acclimatization because a shallow thermocline in the high latitudes restricts most species to the surface layer, whereas the symbiont-bearing species in the low latitudes need to live in the mixed layer to obtain sufficient solar irradiance^78^. This gives us faith that surface ocean temperature is the right approach for the dominant groups and regions. The seasonal range of SST in low latitudes is low (less than 0.5 °C), with only minor differences between the LGM and the pre-industrial, which cannot therefore explain our observation (Supplementary Fig. 6). However, the LGM climate reconstruction is still an active developing topic^62^. Although we include multiple realizations of the modelled LGM climate (cGENIE and HadCM3 as below) and proxy-based temperature^77^, the thermal optima in our study are conditional to the fidelity of these reconstructed climate states.

### LGM and pre-industrial foraminiferal thermal performance curves

#### Quantile regression model

We fit thermal performance curves (norm reaction) using a nonlinear quantile regression model from the R package quantregGrowth^79^. This approach has been used to estimate upper limit functions such as the Eppley curve^80^, which describes the exponential increase in maximum phytoplankton growth rates with temperature. However, the fitted maximum abundances of foraminifera have higher uncertainties in those undersampled regions. To quantify such uncertainty, we apply ten different models with quantile levels from the 90th to the 99th and calculate their mean and s.d. The resulting s.d. that measures the sensitivities of the models to the outlier values could provide an assessment of the sampling effort in this region (Fig. 1).

#### Rendering the optimal temperature

The fitted quantile models are then used to estimate the optimal temperature range at each age. We set half of the maximum abundance (in both the LGM and the pre-industrial pool) as the threshold. The thermal optima of species in the LGM and in the pre-industrial era are provided in Extended Data Table 1.

#### Validating the relative abundance-based optimal niche

To validate the thermal optimum based on relative abundance, we compare the species’ optimal temperatures with previous estimations based on the largest body size^21^ and the highest growth rate^20^. The result shows that the relative abundance-based optimal temperatures are very consistent with those estimations based on biological traits (Extended Data Table 1). Our method also provides the s.d. of the optimal temperature range, which measures the breadth of the optimal niche.

However, we caution our readers about the potential bias when using relative abundance-based optimal niches for rare species. In an extreme scenario, a rare species could have its lowest relative abundance even when it is at its highest absolute abundance. This can occur when a dominant species exists in a similar optimal niche. Nevertheless, this bias is not a substantial concern for dominant species, because their relative abundance is hardly influenced by rare species. Overall, our estimations of optimal niche for dominant species and ecogroup are robust, whereas results for species with a low abundance need to be processed with caution.

#### ANOVA

We conducted two-way ANOVAs in R (v.4.3.1)^81^ to explain the species difference of thermal optimum from the LGM to the pre-industrial era using their symbiosis and spine trait. The full species list and their related trait attribution are provided in Supplementary Table 1.

### Reporting summary

Further information on research design is available in the Nature Portfolio Reporting Summary linked to this article.

## Online content

Any methods, additional references, Nature Portfolio reporting summaries, source data, extended data, supplementary information, acknowledgements, peer review information; details of author contributions and competing interests; and statements of data and code availability are available at 10.1038/s41586-024-08029-0.

## Supplementary information

Supplementary Information This file contains Supplementary Methods, Supplementary Figures 1–7 and Supplementary Tables 1 and 2.

Reporting Summary

Peer Review File

## Source data

Source Data Fig. 1

Source Data Fig. 3

Source Data Extended Data Fig. 2

## Figures and Tables

**Extended Data Table 1:**
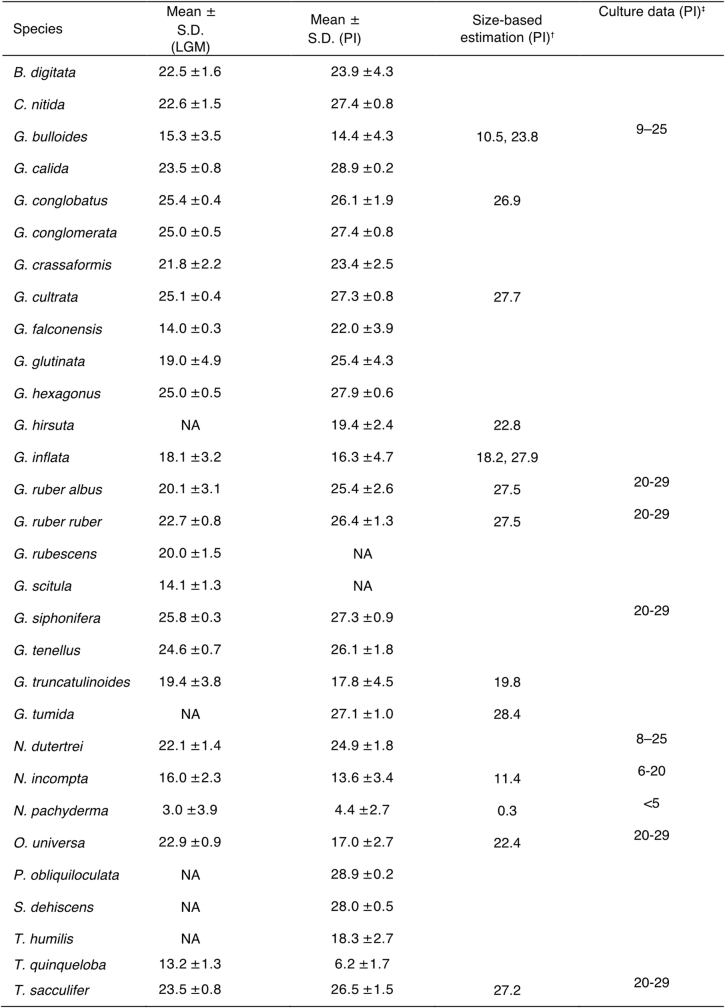
The mean thermal optimum^*^ values and standard deviation for foraminifera species (in degrees Celsius) for the LGM and pre-industrial age

^*^The thermal optimum is defined as the temperature range with more than 50% maximum relative abundance of both LGM and pre-industrial (PI) age. The NA in this table suggests that some taxa satisfy the optimal condition.

^**†**^The optimal temperatures defined as the maximum body size where available in Schmidt et al.^21^. The *G. ruber* in this study include two subspecies, which are separated in our study.

^**‡**^The optimal temperatures defined as the maximum growth rate where available in Lombard et al.^20^.

## Data Availability

The MARGO and ForCenS fossil data, in addition to the previously described^77^ LGM temperature assimilation product, can be retrieved from https://www.pangaea.de. The HadISST data product is publicly available at https://www.metoffice.gov.uk/hadobs/hadisst/. All the CMIP6 data can be downloaded from https://esgf-node.llnl.gov/projects/cmip6/. The cleaned foraminifera fossil abundance and temperature data in this study are available at https://zenodo.org/doi/10.5281/zenodo.8189768 (ref. ^82^). The existing pre-industrial, LGM and future cGENIE model outputs are archived in https://zenodo.org/doi/10.5281/zenodo.8189647 (ref. ^83^). The reanalysed data from a previous study^12^ are available at https://zenodo.org/doi/10.5281/zenodo.8189772 (ref. ^84^). Source data are provided with this paper.

## Code availability

The cGENIE model code used in this study is available at https://github.com/ruiying-ocean/cgenie.muffin/tree/DEV_Foram/ (https://zenodo.org/doi/10.5281/zenodo.12658600). Instructions including commands to run the models can be found in the genie-userconfig/FORAMECOGEM/readme.txt. Model data analyses were performed using cgeniepy (v.0.7.5)^85^. All the scripts for analyses and visualizations are stored at https://github.com/ruiying-ocean/lgm_foram_niche.
