# Supplementary material for: Past foraminiferal acclimatization capacity is limited during future warming
Source: Nature. 2024 Nov 13;636(8042):385–9. doi: 10.1038/s41586-024-08029-0 (PMC11634774; doi:10.1038/s41586-024-08029-0)
Supplement: Supplementary file 1 — This file contains Supplementary Methods, Supplementary Figures 1–7 and Supplementary Tables 1 and 2. [file 41586_2024_8029_MOESM1_ESM.pdf]

nature portfolio

https://doi.org/10.1038/s41586-024-08029-0

Supplementary information

Past foraminiferal acclimatization capacity is limited during future warming

In the format provided by the authors and unedited

Supplementary Methods

1. Mechanistic and trait-based plankton model

1.1 General description

In the modelled surface ocean (0-80.8 m), all plankton have unlimited theoretical niches and can grow anywhere without dispersal limits. The modelled distribution however is constrained by the local environmental conditions including biotic interaction and nutrient/temperature/light conditions driven by other climate components. Such models are referred to as "process-based" or "mechanistic niche" models[86] as the organismal thermal preference is mechanistically determined by the dynamic interaction with the environment, which distinguish themselves from other models that directly parameterise the optimal temperature[69,87]. Each plankton group has different physiological parameters and prey preferences that depend on traits such as body size, spine, and symbiosis, referred to a trait-based model. Our model does not include all traits that could be impacted by temperature; for example, we do not include the differential traits during ontogenetic development. Furthermore, the foraminifera growth in this model is not impacted by carbon chemistry and oxygen environment.

1.2 Model implementation and improvement

We use a foraminifera-centred ecosystem configuration in the intermediate complexity model cGENIE, based on the previously published version[17]. The modelled plankton community includes eight generic phytoplankton, seven zooplankton and four planktic foraminifer types (ecogroups) imposed to grow in the euphotic zone (0-80.8 m). The phytoplankton and zooplankton types differ by their size classes (0.6, 1.9, 6.0, 19.0, 60.0, 190.0, 600.0, and 1900.0 μm). Besides, four foraminifera ecogroups are included: symbiont-barren non-spinose foraminifera, symbiont-barren spinose foraminifera, symbiont-facultative non-spinose foraminifera, and symbiont-obligate spinose foraminifera. The symbiotic foraminifera are modelled as mixotrophs with reduced autotrophic and heterotrophic efficiency. However, because of the poor biological understanding of symbiont-facultative foraminifera, we only use their model output in calculating relative abundance. It is also worth noting that the model allows all the plankton to live under sea ice, which is a key trait for some foraminifera species in the polar regions and thrives in the glacial condition[88].

In this study, we have made several improvements based on our previous published configuration[17] (Supplementary Table 2). The previous model configuration agreed well with the geographic distribution of modern relative abundance and presented realistically low biomass in agreement with the observations (global mean foraminiferal biomass: 0.002-0.005 mmol C m^{-3} varied by ecogroup). However, plotting the modelled relative abundance against temperature showed an unrealistic increase in symbiont-obligate foraminifera's abundance at low temperatures (10 °C; Supplementary Fig. 5). Therefore, the model needed improvement of foraminiferal ecology to be more suitable for the niche exploration in this paper.

To achieve a more realistic ecology of different ecogroups under modern and LGM conditions, our modifications fully leverage the established knowledge. We now use body size for different ecogroups which more closely reflects the size differences in the modern ocean (200.0 μm for symbiont-barren foraminifera and 300 μm for symbiont-obligate and symbiont-facultative foraminifera), instead of using a uniform value (190 μm)[21]. A similar adjustment has been done for the foraminiferal symbiont (now 3 μm compared to the previous 0.285 μm) according to the culture experiment[89]. We also improved the spine trait representation compared to the previously implemented enhanced foraminiferal grazing

efficiency by reducing half-saturation concentration in the grazing function. The spine now allows foraminifera to be virtually several times larger than its size in the process of prey selection and therefore can capture larger zooplankton (double or triple the foraminiferal size)[90]. To achieve the opportunistic behaviour of spinose foraminifera such as the *G. bulloides* and *T. quiqueloba*[74], we also increased the prey-switching exponent so that spinose foraminifera preferentially eat abundant prey in eutrophic conditions. Finally, the modelled symbiont-obligate spinose foraminifera now feed on zooplankton, while the symbiont-barren spinose foraminifera feed on phytoplankton according to ref[90].

We also used the opportunity to fine-tune several model parameters. This includes the maximal grazing rate of non-spinose foraminifera, the mortality protection percentage caused by calcification, and the respiration cost of building calcification and spine (Supplementary Table 2). We did this because the high energetic cost of building a shell and spine relative to the growth rate in the previous model fails to reproduce a viable population in the Last Glacial Maximum due to very low standing stocks. The new parameter values are chosen from the previously tested range in a 0D model[39]. Our new parameterisation (Supplementary Table 2) now achieves a viable population in the Last Glacial Maximum while resulting in an overestimate of foraminifera biomass in the modern ocean (0.017-0.04 mmol C m^{-3}). This overestimated modern biomass however still compares better than other foraminifera models in the literature such as the PLAFOM[69]. Combined with our improved foraminifera spine, feeding behaviour and size, the new parameterisation enables our model to compare better with the modern estimate of the thermal niche (Supplementary Fig. 5), which gives us higher confidence in applying the model in the future and the LGM.

1.3 Differential equation of biomass and nutrient dynamic

Based on the above parameterisations, the biomass dynamic of each plankton is calculated based on a general differential equation:

$$\frac{\partial B_{j,i_b}}{\partial t} = \underbrace{P_{j,C} \cdot B_{j,C}}_{carbon\ fixation} + \underbrace{\mu_{j,i_b} \cdot B_{j,C}}_{nutrient\ uptake} + \underbrace{B_{j,C} \cdot \lambda_{i_b} \sum_{j_{prey}=1}^{J} G_{j,j_{prey},i_b}}_{grazing\ gains}$$

$$- \underbrace{B_{j_{pred},C} \cdot \sum_{j_{pred}=1}^{J} G_{j_{pred},j,i_b}}_{grazing\ losses} - \underbrace{m_j \cdot B_{j,i_b}}_{mortality\ loss} - \underbrace{r_{j,C} \cdot B_{j,i_b}}_{respiration\ loss}$$

B_{j,i_b} stands for the carbon and nutrient biomass (P, Fe, Chl denoted by i_b) of each plankton functional type (denoted by j). The carbon fixation from photosynthesis ($P_{j,C}$), nutrient uptake (μ_{j,i_b}), grazing activities (G_{j_{pred},j,i_b}), mortality loss (m_j) and respiration loss ($r_{j,C}$) in each model time step is scaled to the plankton carbon biomass ($B_{j,C}$).

The inorganic nutrient state variables (P, Fe denoted by i_r) are influenced by the nutrient uptake of phytoplankton and foraminiferal symbionts:

$$\frac{\partial R_{i_r}}{\partial t} = \sum_{j=1}^{J} \mu_{j,i_r} \cdot B_{j,C}$$

The other biogeochemical processes including remineralisation of organic matter are handled in BIOGEM[52]. We refer our readers to find full descriptions in Ying et al.[17] and Ward et al.[16].

1.4 The causes of thermal niche change

The thermal performance curve describes the organism's fitness as a function of temperature (i.e., $B_j = f(T)$). Similarly, the rate of all ecological processes can also be written as a function of temperature: photosynthesis $f_p(T)$, nutrient uptake $f_n(T)$, net grazing input $f_g(T)$, mortality $f_m(T)$, and respiration $f_r(T)$. However, many processes here are not only limited by temperature but also other environmental factors. This means that the corresponding thermal function is variable, and our goal is to find such variability in this system.

We focus on the carbon biomass and therefore ignore the nutrient uptake part. We also ignore the grazing loss of foraminifera as described in section 1.4.3. Substituting the $f(T)$ and its components into the differential equation of biomass describes how the thermal niche $f(T)$ changes through model time steps. The model has the same initial condition of plankton biomass in different experiments. Therefore, the final thermal niche is eventually determined by the rate of each subprocess.

$$\frac{d}{dt}f(T) = [f_p(T) + f_g(T) + f_m(T) + f_r(T)]\, B_C$$

1.4.1 Respiration rate

The respiration (r_j) is modulated by the basal respiration rate ($r_{j,b}$) and ambient temperature (γ_T). In the model, all the temperature dependencies are governed by the Arrhenius-like function.

$$r_j = r_{j,b} \cdot \gamma_T$$
$$\gamma_T = e^{A(T-T_{ref})}$$

The parameter A determines temperature sensitivity and reference temperature (T_{ref}) is the temperature allowing $\gamma_T = 1$. Therefore, if a process like respiration is only determined by environmental temperature, its contribution to thermal niche changing rate $f_r(T)$ does not change across different climate states. Following this logic, we explore how other different physiological processes contribute to the observed thermal niche shift.

1.4.2 Mortality loss rate

Mortality loss rate (m_j) is a constant subject to calcification protection coefficient ($P_{m,j}$) but independent on temperature. Therefore, the mortality contribution to the thermal niche changing rate $f_m(T)$ is a constant and should not cause different thermal niche responses.

$$m_j = P_{m,j} \cdot m_{b,j}$$

1.4.3 Grazing activities

The grazing activities include three parts: prey selection, grazing, and assimilation. The prey selection and grazing rate depends on the predator and prey body size (optimal size ratio is greater for spinose foraminifera; greater size leads to lower maximum ingestion rate), and the assimilation rate depends on the nutrient content of the predator. For foraminifera, there is no specialist predator[74] and their modelled optimal (based on size) predators are mesozooplankton with 10 times larger body size and the lowest ingestion rate[39]. The model also assumes a reduced palatability of foraminifera as a benefit of calcification, which further reduces their grazing pressure. Finally, foraminifera have a low abundance in the water, which could help their survival from predation. Overall, the grazing pressure for foraminifera is negligible as the previous study suggested[39], and we focus on the impacts of their grazing gains on the thermal niche.

The grazing function follows a Holling II equation, in which G_{max} is the maximum grazing rate (a constant estimated by body size), F is the prey concentration, k is the half-saturation concentration, λ is the assimilation efficiency.

$$g = G_{max} \cdot \frac{F}{F + k} \cdot \lambda \cdot \gamma_T$$

The assimilation rate λ is a constant scaled by the current nutrient quota (γ_{quota}). It is based on the variable stoichiometry and the more nutrient foraminifera currently have, the lower the assimilation rate it will be, and *vice versa*.

$$\lambda = \lambda_m \cdot \gamma_{\text{quota}}$$

Therefore, the grazing contribution to thermal niche changing rate $f_g(T)$ can be driven by two variables: the prey concentration (food availability F) and the assimilation efficiency (food quality λ).

1.4.4 Photosynthesis rate

The autotrophic and mixotrophic plankton, including symbiotic foraminifera, can take up nutrients and perform photosynthesis. In the model, the photosynthesis rate is a function of temperature (γ_T), irradiance level (γ_I), nutrient availability and current nutrient quota ($\min[\gamma_P, \gamma_{Fe}]$), and a size-based maximum rate (P_C^m).

$$P_C = P_C^m \cdot \min[\gamma_P, \gamma_{Fe}] \cdot \gamma_T \cdot \gamma_I$$

Therefore, the symbiosis contribution to the thermal niche changing rate $f_p(T)$ is determined by the environmental light intensity γ_I, nutrient availability and current nutrient quota (γ_P, γ_{Fe}). However, it is worth noting that this process is less influential for foraminifera with discounted autotrophic efficiency as the cost of being symbiotic.

1.4.5 Net primary productivity

Based on the descriptions above, we can summarise that the thermal niche shift is associated with the current nutrient quota and food availability, which also indirectly reflects the nutrient availability and light intensity. In the main text, we use the carbon-to-phosphate ratio (the stoichiometry) to reflect the nutrient quota of foraminifera.

We then use the net primary productivity as an indicator of the rest of the variables: nutrient availability, light intensity, and the consequent total food supply. This is because the modelled zooplankton can capture any phytoplankton (or zooplankton if carnivorous) group despite different preferences. The net primary productivity can be calculated by summing all plankton groups' net carbon fixation. It is influenced by temperature, nutrient availability, light intensity, and the dynamic interaction of the marine plankton food web.

$$NPP = \sum_{j=1}^{J} \left(P_C^j - r_C^j \right)$$

2. Antell et al. (2021) niche data reanalysis

The evidence of thermal acclimation from LGM to PI appears to contradict previous studies that postulated foraminifera-stable niches[12,13]. The latest of these studies[12] used foraminiferal presence data of 24 species and model-derived temperature to estimate the probability distribution function of foraminifera temperature over the Quaternary glacial-interglacial cycles (0-700 ka). They found that foraminifera niches were overall stable (less than 20% dissimilarities) over time, without significant correlation with the change in the mean annual temperature. However, the total realised niche can respond differently from the optimal niche, and, therefore, this difference could mask the adaptive capacities of foraminifera in the late Quaternary glacial-interglacial cycles.

To demonstrate the difference between optimal niche and overall niche, we reanalysed presence-based optimal temperatures (Antell et al. 2021). Here, we only reanalysed the LGM and Holocene parts of this dataset because (1) longer-term datasets are characterised by fewer samples and more uncertainties in estimated optimal temperatures; (2) the thermal optimum in Antell et al. (2021) was quantified as the temperature with the highest likelihood of occurrence, thus less accurate than of estimates based on abundance data; and (3) unlike the LGM, it is more challenging to distinguish adaptation from acclimation in longer-term records.

In the presence-based data, most foraminifera species in the Holocene bin (0-8 ka) exhibit similarly higher optimal temperatures than the LGM bin (16-24 ka) (-0.3 to 8.6 °C; Extended Data Fig. 5). For example, the optimal temperature of *G. ruber* (white and pink merged) increased by 4.5 °C (5.3 °C for *G. ruber albus* and 3.7 °C for *G. ruber ruber* in our data). This value is 1.9 °C for *N. dutertrei* (2.8 °C in our results) and 0.7 °C for *G. siphonifera* (1.8 °C in our results). The *G. bulloides* in this dataset has a striking difference between the LGM and PI optimal temperatures (8.6 °C) likely because it does not sufficiently capture the tropical genotypes in the LGM. Overall, notwithstanding the uncertainty of niche estimation, the Antell et al. (2021) data indicates the same optimal niche changes as our results, although they found that the overall foraminiferal niche was stable.

3. Model sensitivity experiments on the impact of different warming rates

To assess the impact of warming time scale on ocean circulation and nutrient distribution, we ran a series of model experiments at different warming rates. We forced the model to linearly reach the 1000 ppm CO_2 over long timescales (by 3000, 4000, 5000) and picked out the years (2540, 2990, 3460) when the ocean surface warming levels are the same as the 2100 year in the +4 °C pathway (22.57 °C) (Extended Data Fig. 8). We also performed a completely spun-up simulation with the same warming level as the 2100 +4 °C transient experiment (global mean SST: 22.58 °C) after running for 10,000 years with a 2.5×PI CO_2 setting (695 ppm). These experiments thereby constitute a gradient of warming rate. We then assessed, the impact of warming timescale comparing the surface nutrient and foraminiferal biomass in those "slower warming" experiments with the fastest experiment (+4 °C in 2100). This is an ideal test case as our model does not simulate many timescale-sensitive climatic dynamics because of its intermediate physical complexity. For instance, the model uses prescribed 2D wind stress and dust emission field, which will not evolve in any model years. Therefore, the simulated future warming at a millennium scale here is highly simplified and focuses on the changes of overturning circulation.

The simulation results show that the increasing surface warming causes ocean stratification. The nutrients therefore are not delivered to the surface as efficiently and accumulate in the deep (Extended Data Figs. 7-8). However, in a scenario of slower warming where stratification is limited and in a fully equilibrium state, the surface nutrient will exceed the fastest emission pathway (Extended Data Fig. 8). Therefore, the foraminifera biomass will be higher if the warming is slower despite the same warming magnitude. This difference is mostly evident in the Southern Hemisphere but absent in the North Atlantic (Extended Data Fig. 9), where the AMOC slows down and continues to exacerbate nutrient depletion for a longer time.

Supplementary Tables

Supplementary Table 1. The planktic foraminifer species list and their ecogroup division

No	Species name[a]	Author	Symbiosis[b]	Spine[c]
1	*Beella digitata*	Brady, 1879	symbiont-barren	spinose
2	*Berggrenia pumilio*	Parker, 1962	undetermined	non-spinose
3	*Bolivina variabilis*	Williamson, 1858	undetermined	undetermined
4	*Candeina nitida*	d’Orbigny, 1839	symbiont-facultative	non-spinose
5	*Dentigloborotalia anfracta*	Parker, 1967	undetermined	non-spinose
6	*Globigerina bulloides*	d’Orbigny, 1826	symbiont-barren	spinose
7	*Globigerina falconensis*	Blow, 1959	symbiont-obligate	spinose
8	*Globigerinella adamsi*	Banner and Blow, 1959	symbiont-barren	spinose
9	*Globigerinella calida*	Parker, 1962	symbiont-obligate	spinose
10	*Globigerinella radians*	Egger, 1893	undetermined	spinose
11	*Globigerinella siphonifera*	d’Orbigny, 1839	symbiont-obligate	spinose
12	*Globigerinita glutinata*	Egger, 1893	symbiont-facultative	non-spinose
13	*Globigerinita minuta*	Natland, 1938	undetermined	non-spinose
14	*Globigerinita uvula*	Ehrenberg, 1862	symbiont-bearing	non-spinose
15	*Globigerinoides conglobatus*	Brady, 1879	symbiont-obligate	spinose
16	*Globigerinoides elongatus*	d’Orbigny, 1826	undetermined	spinose
17	*Globigerinoides ruber albus*	Morard et al., 2019	symbiont-obligate	spinose
18	*Globigerinoides ruber ruber*	d’Orbigny, 1839	symbiont-obligate	spinose
19	*Globigerinoides tenellus*	Parker, 1958	symbiont-bearing	spinose
20	*Globoquadrina conglomerata*	Schwager, 1866	undetermined	non-spinose
21	*Globorotalia cavernula*	Bé, 1967	undetermined	non-spinose
22	*Globorotalia crassaformis*	Galloway and Wissler, 1927	symbiont-barren	non-spinose
23	*Globorotalia cultrata*	d’Orbigny, 1839	symbiont-facultative	non-spinose
24	*Globorotalia eastropacia*	Boltovskoy, 1974	undetermined	non-spinose
25	*Globorotalia hirsuta*	d’Orbigny, 1839	symbiont-barren	non-spinose
26	*Globorotalia inflata*	d’Orbigny, 1839	symbiont-facultative	non-spinose
27	*Globorotalia scitula*	Brady, 1882	symbiont-barren	non-spinose
28	*Globorotalia truncatulinoides*	d’Orbigny, 1839	symbiont-barren	non-spinose
29	*Globorotalia tumida*	Brady, 1877	symbiont-barren	non-spinose
30	*Globorotalia ungulata*	Bermúdez, 1961	undetermined	non-spinose
31	*Globorotaloides hexagonus*	Natland, 1938	symbiont-barren	non-spinose
32	*Globorotaloides oveyi*	Buckley, 1973	undetermined	undetermined
33	*Globoturborotalita rubescens*	Hofker, 1956	symbiont-bearing	spinose
34	*Hastigerina pelagica*	d'Orbigny, 1839	symbiont-barren	spinose

35	*Hastigerinella digitata*	Rhumbler, 1911	symbiont-barren	spinose
36	*Neogallitellia vivans*	Cushman, 1934	undetermined	undetermined
37	*Neogloboquadrina dutertrei*	d'Orbigny, 1839	symbiont-facultative	non-spinose
38	*Neogloboquadrina incompta*	Cifelli, 1961	symbiont-barren	non-spinose
39	*Neogloboquadrina pachyderma*	Ehrenberg, 1862	symbiont-barren	non-spinose
40	*Orbulina universa*	d'Orbigny, 1839	symbiont-obligate	spinose
41	*Orcadia riedeli*	Rögl and Bolli, 1973	undetermined	spinose
42	*Pulleniatina obliquiloculata*	Parker and Jones, 1865	symbiont-facultative	non-spinose
43	*Sphaeroidinella dehiscens*	Parker and Jones, 1865	symbiont-bearing	spinose
44	*Tenuitellita fleisheri*	Li, 1987	symbiont-barren	non-spinose
45	*Tenuitellita iota*	Parker, 1962	undetermined	non-spinose
46	*Tenuitellita parkerae*	Brönnimann and Resig, 1971	undetermined	non-spinose
47	*Trilobatus sacculifer*	Brady, 1877	symbiont-obligate	spinose
48	*Turborotalita clarkei*	Rögl and Bolli, 1973	symbiont-barren	spinose
49	*Turborotalita humilis*	Brady, 1884	symbiont-facultative	spinose
50	*Turborotalita quinqueloba*	Natland, 1938	symbiont-barren	spinose

[a] The taxonomy is based on Brummer and Kucera (2022)
[b] The symbiotic ecology is based on Takagi et al. (2019)
[c] The spine information is based on Schiebel and Hemleben (2017) and mikrotax (https://www.mikrotax.org).

Supplementary Table 2. A summary of changes in the foraminifera model parameters compared to the Ying et al. (2023) configuration

		Symbiont-barren non-spinose	Symbiont-barren spinose	Symbiont-facultative non-spinose	Symbiont-obligate spinose
Equivalent spherical diameter (µm)	Ying2023	190	190	190	190
	This study	200	200	300	300
Symbiont/host size ratio	Ying2023			0.0015	0.0015
	This study			0.01	0.01
Feeding prey	Ying2023	phytoplankton	phytoplankton	phytoplankton	phytoplankton
	This study	phytoplankton	phytoplankton	phytoplankton	zooplankton
Size amplification by spine (unitless)[a]	Ying2023	-	-	-	-
	This study	-	3.5	-	3.5
Sensitivity to food availability (unitless)	Ying2023	1	1	1	1
	This study	1	5	1	5
Maximal grazing rate (unitless)	Ying2023	-	-	-	-
	This study	+30%[b]	-	+30%	-
Mortality reduction by calcification (unitless)	Ying2023	+40%[c]	+40%	+40%	40%
	This study	+30%	+30%	+30%	+30%
Respiration cost by calcification (d^{-1})	Ying2023	0.01	0.01	0.01	0.01
	This study	0.02	0.02	0.02	0.02
Respiration cost by spine (d^{-1})	Ying2023	0.02	0.02	0.02	0.02
	This study	0.025	0.025	0.025	0.025

[a] Spinose foraminifera have virtually large body sizes (here 3.5 times larger) to feed larger prey.
[b, c] This percentage is relative to the background value determined by allometric scaling.

Supplementary Figures

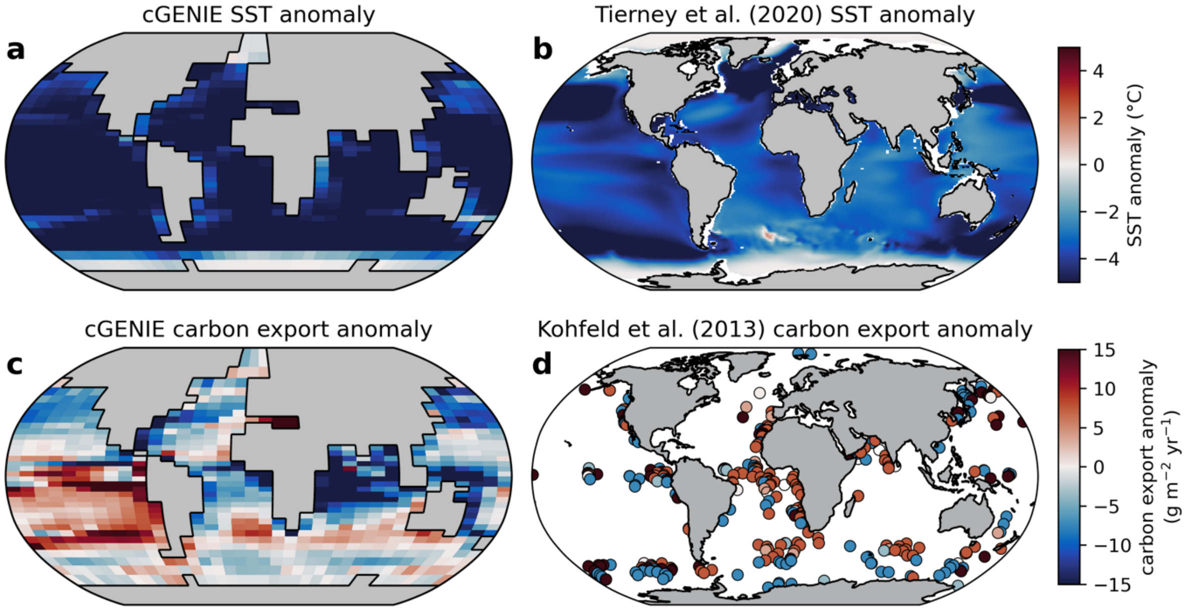

Supplementary Figure 1. Model-data comparison of the sea surface temperature and carbon export anomaly between the Last Glacial Maximum and the pre-industrial age. (a) The cGENIE model shows a 5 ± 1.5 °C (global mean ± s.d.) cooling of sea surface temperature (LGM minus PI), which overlaps with (b) the 3.2 ± 1.8 °C cooling estimated in the Tierney et al. (2020) data assimilation. (c) The modelled carbon export productivity at 80.8 m is regionally higher in the LGM compared to the PI, in agreement with (d) the proxy data compilation. Note that the export productivity anomaly in Kohfeld et al. (2013) is qualitative.

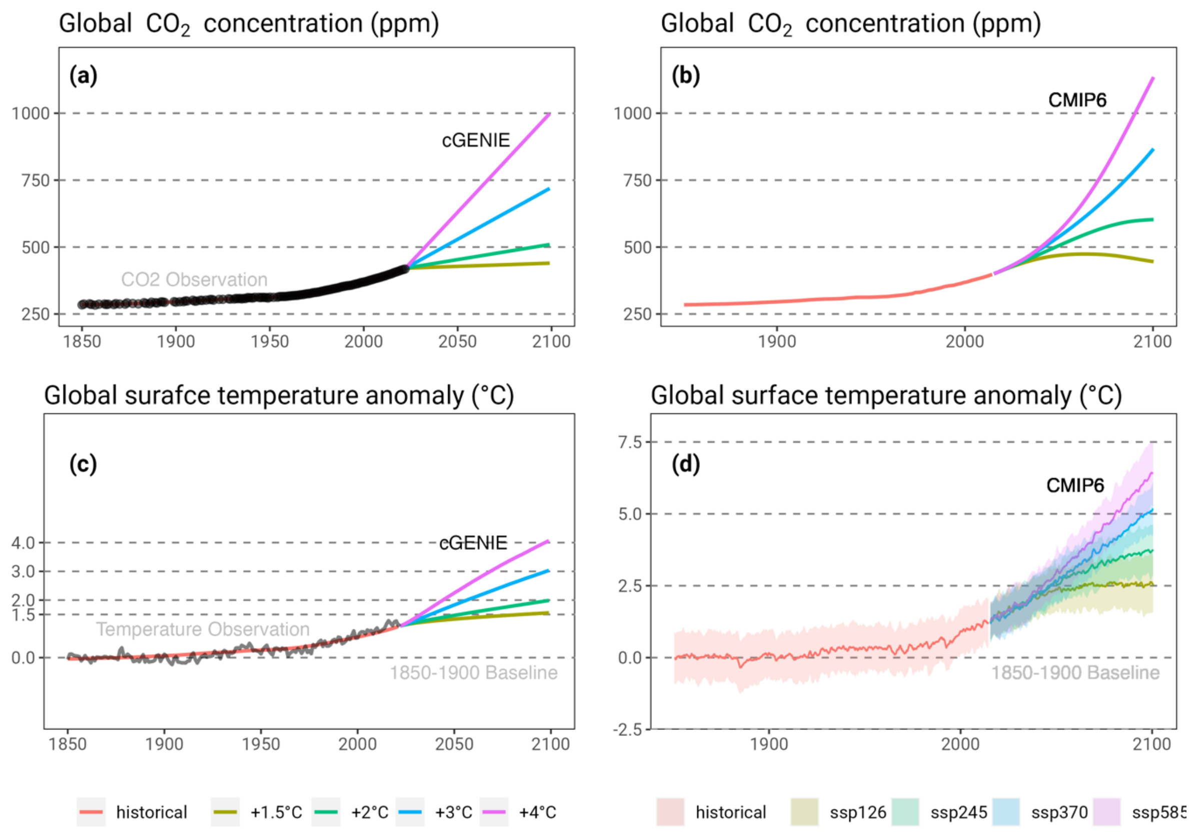

Supplementary Figure 2. CO_2 forcing (a, b) and consequent global mean surface temperature changes (c, d) in our future experiments using cGENIE in the context of CMIP6 scenarios. Other greenhouse gases are not included in our idealised experiments. The historical observation of temperature comes from HadCRUT5 (https://www.metoffice.gov.uk/hadobs/hadcrut5/) and the historical observation of CO_2 from National Oceanic and Atmospheric Administration (NOAA).

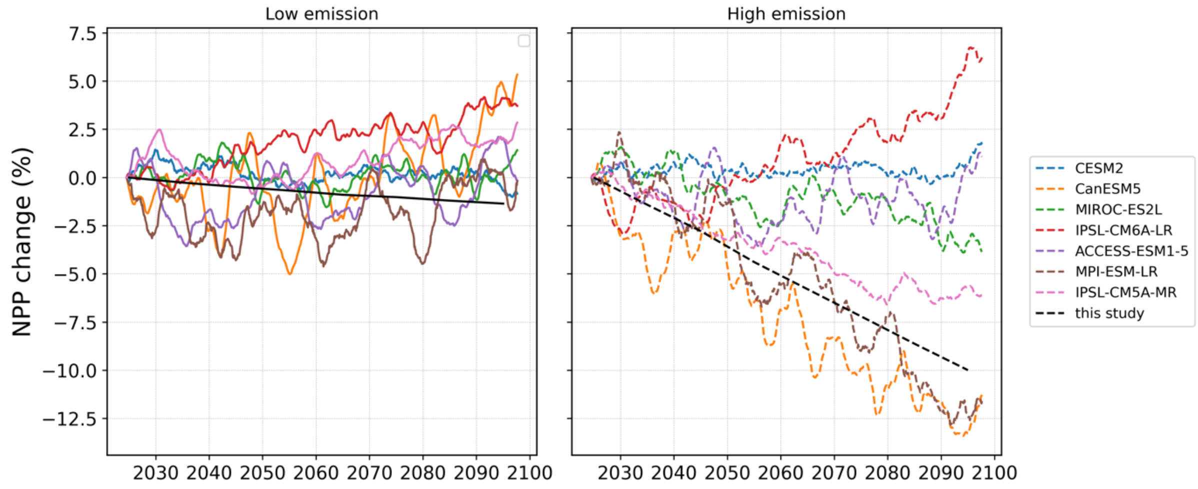

Supplementary Figure 3. The projected net primary production (NPP) change of cGENIE (black) is consistent with CMIP5 and CMIP6 model predictions (coloured). Left: low emission and high mitigation pathway (RCP 2.6 and SSP 1-2.6 for CMIP models and 1.5 °C for cGENIE). Right: high emission and low mitigation pathway (RCP 8.5 and SSP 5-8.5 for CMIP models and 4 °C for cGENIE). cGENIE in high emission pathway is more consistent with the model version with lower resolution (e.g., MPI-ESM-LR with 1.5°, CanESM5 with 2.8°).

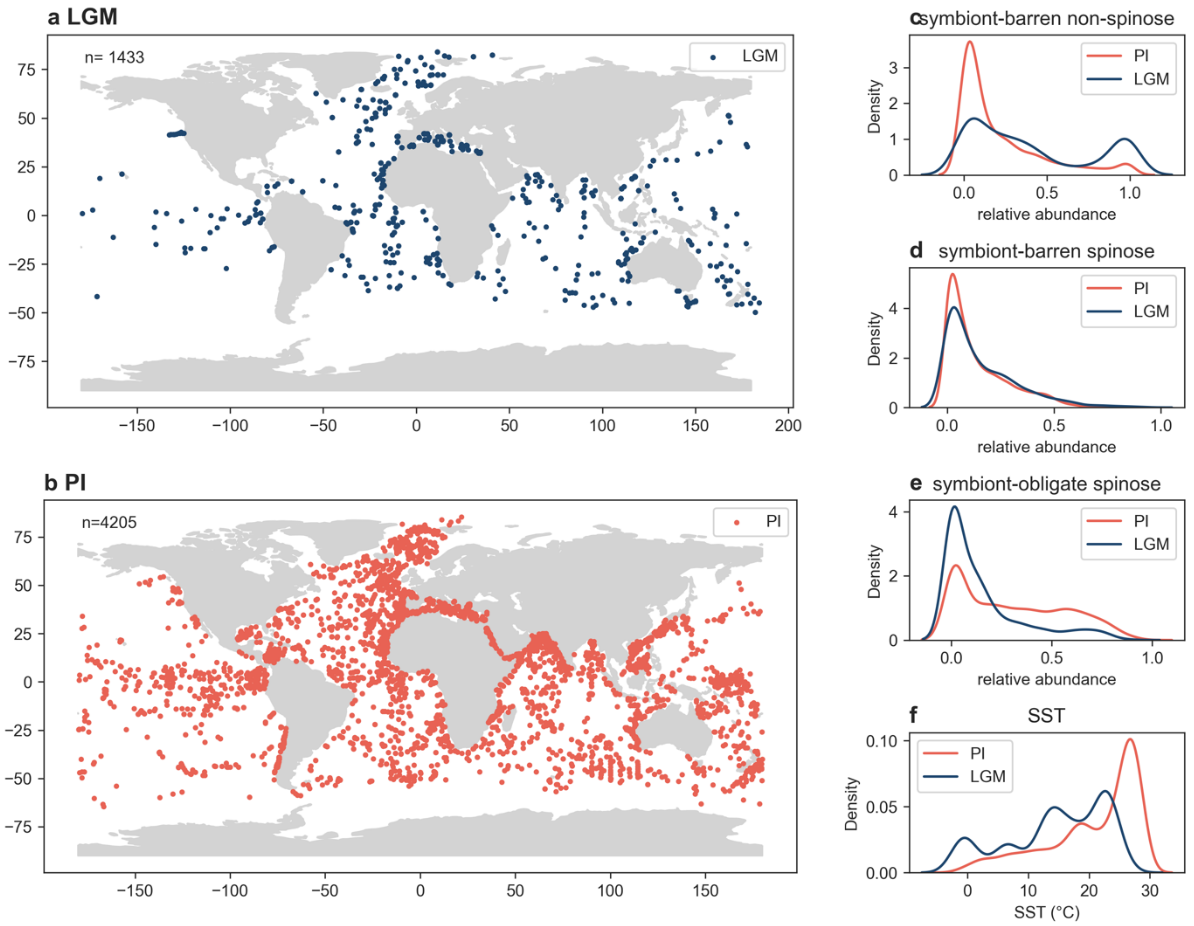

Supplementary Figure 4. The sampling efforts of LGM and PI samples used to determine foraminifera relative abundance. (a-b) There are 4205 available data points in the pre-industrial (41 species) and 1433 data points for the LGM (39 species). (c-e) The distribution of each ecogroup's relative abundance in the LGM (blue) and PI (red) reflects the decreased relative abundance of symbiont-barren non-spinose and increased relative abundance of symbiont-obligate spinose during the deglacial warming. (f) the sampling efforts over temperature show denser sampling in low latitudes in both LGM and PI.

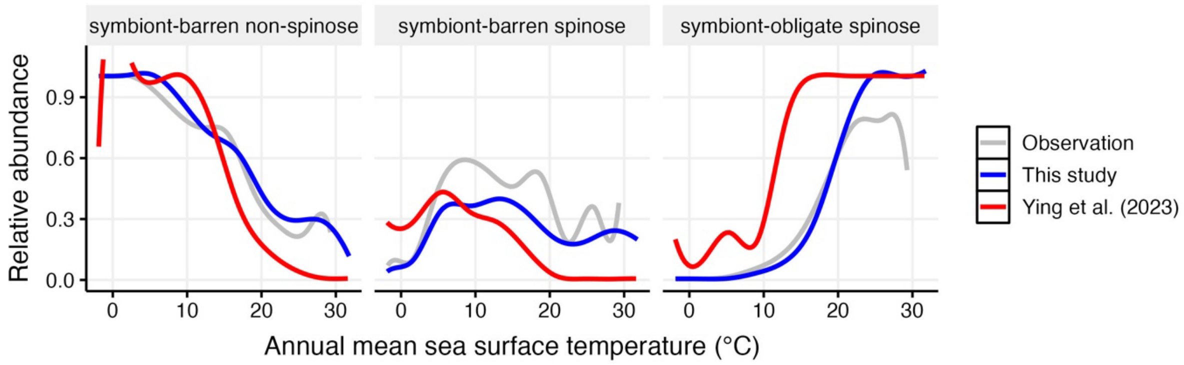

Supplementary Figure 5. Model-data comparison of the modern foraminiferal thermal performance curve. Our previous model configuration (Ying et al., 2023) tends to overestimate the relative abundance of symbiont-obligate spinose foraminifera in the temperate region (10-20 °C) and underestimate the symbiont-barren spinose group from 10-30 °C. The model configuration used in this study improves the foraminifera ecology and compares better with the modern observational data.

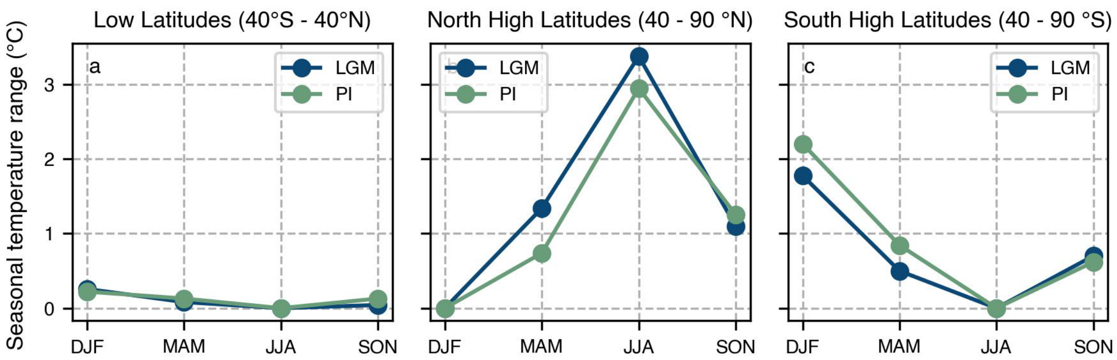

Supplementary Figure 6. Modelled seasonally averaged cycles of LGM and PI sea surface temperature for three main geographic areas. We average the temperature for each latitudinal band and calculate the mean temperature every three months. The seasonal temperature range is weakest in (a) low latitudes (40 °S - 40 °N) and strongest in (b) high latitudes in the North Hemisphere (40 - 90 °N), with (c) high latitudes in the Southern Hemisphere (40 - 90 °S) in the middle. The comparison between LGM and PI shows that the impact of seasonality change on thermal optima shift is negligible.

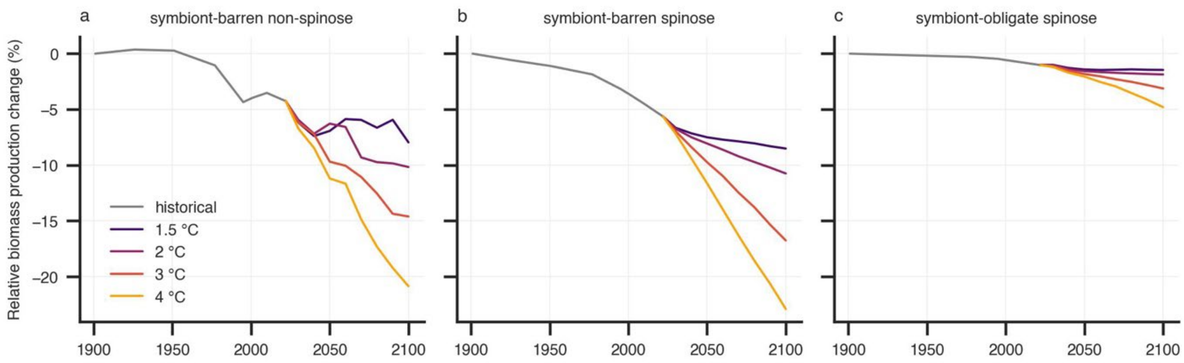

Supplementary Figure 7. Projected globally integrated carbon biomass production of each foraminifer ecogroup from 2022 to 2100 in response to 4 different warming scenarios. The biomass production is calculated as the source term of biomass differential equations (i.e., the biomass input). For symbiont-obligate foraminifera, this includes both grazing source term and symbionts' photosynthesis source term. This figure shows that foraminiferal biomass production has similar trend with biomass standing stock (Extended Data Fig. 6).

Supplementary References

86. Litchman, E., Edwards, K., Klausmeier, C. & Thomas, M. Phytoplankton niches, traits and eco-evolutionary responses to global environmental change. *Mar. Ecol. Prog. Ser.* **470**, 235–248 (2012).
87. Lombard, F. *et al.* Modelling planktic foraminifer growth and distribution using an ecophysiological multi-species approach. *Biogeosciences* **8**, 853–873 (2011).
88. Greco, M., Jonkers, L., Kretschmer, K., Bijma, J. & Kucera, M. Depth habitat of the planktonic foraminifera Neogloboquadrina pachyderma in the northern high latitudes explained by sea-ice and chlorophyll concentrations. *Biogeosciences* **16**, 3425–3437 (2019).
89. Spero, H. J. & Parker, S. L. Photosynthesis in the symbiotic planktonic foraminifer Orbulina universa, and its potential contribution to oceanic primary productivity. *Journal of Foraminiferal Research* **15**, 273–281 (1985).
90. Anderson, O. R., Spindler, M., Bé, A. W. H. & Hemleben, Ch. Trophic activity of planktonic foraminifera. *J. Mar. Biol. Ass.* **59**, 791–799 (1979).
