# Supplementary material for: Past foraminiferal acclimatization capacity is limited during future warming
Source: Nature. 2024 Nov 13;636(8042):385–9. doi: 10.1038/s41586-024-08029-0 (PMC11634774; doi:10.1038/s41586-024-08029-0)
Supplement: Supplementary file 3 — Peer Review File [file 41586_2024_8029_MOESM3_ESM.pdf]

Peer Review File

Manuscript Title: Past foraminiferal acclimation capacity is limited during future warming

Reviewer Comments & Author Rebuttals

Reviewer Reports on the Initial Version:

Referees' comments:

Referee #1 (Remarks to the Author):

The manuscript by Yin and colleagues combines simulations using a trait-based ecological model with observations from the past to assess the temporal stability of the thermal performance of marine plankton. The authors find that the thermal optima of two ecogroups of the zooplankton they analysed were different (lower) during the last ice age. However, when simulating future warming they observe no change in the thermal optima, indicating that this group of zooplankton will migrate to accommodate projected changes in the environment. This migration is projected to be associated with a global reduction in biomass of the group.
The manuscript is well written and elegant in its simplicity. The insights are potentially relevant for both the interpretation of the fossil record as well as for the modelling of the response of marine plankton to anthropogenic (climate) change. The results are also novel and original and as far as I can judge the statistical analyses are appropriate. As such, I think the study is potentially of interest to a wide audience and should be considered for publication in Nature provided some concerns are adequately addressed in a revised version. My main concerns centre on i) the attribution of the changes in the thermal optima to acclimatisation, ii) the mechanism that could explain why/how the thermal optima changed, iii) the comparison with the fossil record, and iv) the comparison with previous work that showed opposite results. I have detailed these concerns below and hope that they help to improve the study.

Lukas Jonkers

- Acclimatisation
My first comment relates to the concept of acclimatisation and whether or not the results unambiguously point to plasticity in the environmental niche, or rather to different parts of the fundamental niche being occupied. This distinction between the fundamental niche (the environmental space that can theoretically be occupied) and the realised niche (sometimes called habitat: the part of the fundamental niche that is occupied) is important. Changes in the fundamental niche would point to changes in the physiology of the organism (perhaps true acclimatisation), whereas changes in the realised niche could also result from other processes, for instance biological interactions or environmental change. I think the authors can only infer the realised niche from their simulations and they should make this clearer in the text. I mention this because it has important implications. If the changes in the thermal optima are really reflected in the fossil data (see below), then the authors need to discuss these in the light of this distinction between fundamental and realised niche and argue why

the observations point to acclimatisation. To address this issue the authors need to provide more careful and precise wording.
The clearest case where I have my doubts is in the warm-water species (symbiont-obligate spinose): temperatures above 25 degC are simply absent from the LGM simulation/reconstruction. The emergence of new (warm-water) habitats during the Holocene ia associated with a shift in the thermal optimum, but does not require physiological changes in the organisms as it could simply reflect the colonisation of these new habitats. Similarly, changes in the symbiont-barren species could also simply be due to a different portion of the fundamental niche being occupied as a result of changing (a)biotic interactions.
In addition, it remains unclear what temperature (at which depth and season) is used to characterise the niche (in both the simulation and the reconstruction) and the authors should comment on the possibility that the temperature plotted on the x-axis in figure 1 was not exactly the temperature at which the foraminifera lived. Could, for instance, a different seasonal temperature range during the LGM explain part of the change in the optima? The authors should be able to obtain the relevant information from their model. It's harder for the fossil data, but the possibility should be assessed. Again, this comes down to the question whether the purported change in the thermal optima really reflects acclimatisation, and if alternative explanations can be ruled out.

- Mechanism and relevance
My second comment is related to the first, but focuses on the mechanism that could be responsible for the change in the optima. Compared to other planktonic foraminifera models, a key advantage of the model set up used in this study is that the thermal performance emerges from the simulations. This means that the authors should be able to dig deeper into the model output and point at the mechanism that drives the changes in the thermal performance. As it stands we learn little new about the ecology of planktonic foraminifera, even though such insights may help to better anticipate future changes and better understand the fossil record. I think this really is a missed opportunity and encourage the authors to explore the model output.
The suggestion the authors make is that the difference between the simulated response for the LGM and the future is in part related to the pace of environmental change: slow during the deglaciation and fast into the future ("Our study highlights the importance of the timescale of warming on determining plankton acclimation and migration.", lines 29-31). This may be true, but they lack the data to conclude this. They ran time slice (equilibrium) simulations for the LGM and the PI and thus, by definition, cannot make any statements about the speed (or the timing, for that matter, e.g. "during the last deglacial warming" L24-25) of the apparent acclimatisation. What would be needed to test this hypothesis are transient simulations where the temperature changes from LGM to PI conditions at rates comparable to the observations (over millennia) and artificially increased rates that are comparable to the projected warming. I am not saying that the authors should perform such analyses, but that they should investigate alternative explanations for their observations, because there must be other reasons since they show up in the equilibrium simulations.

In addition, the authors hint at implications of their study for other plankton groups, even marine plankton at large. It is however unclear to me how representative the results are for other plankton and

I am surprised that this is not explored in the simulations. The authors could assess in their simulations how representative planktonic foraminifera are for phyto/zooplankton and/or connect better with the literature to demonstrate that the plasticity implied for foraminifera also characterises other plankton. In short, the study needs to provide more convincing arguments that the findings for planktonic foraminifera have bearings beyond this group.

- Comparison with observations

Whilst my first two comments concerned the interpretation of the results, I am also not yet convinced that the fossil record really supports the conclusions from the modelling. This is partly due to methodological issues that render it difficult to characterise the thermal optima (see below) and the fact that all species including the symbiont-barren non-spinose show apparent changes in the niche (Fig. S3) even though the simulations suggest otherwise (Fig. 1). Moreover, the conclusion that the changes are real ultimately depends on how well the reconstructed temperatures represent those where and when the foraminifera lived. Since the reconstruction that the authors used is based on a single climate model and has a spatial correlation structure that is essentially the same as in that model, it remains controversial (e.g. (Annan, Hargreaves, and Mauritsen 2022)). This means that the support for the conclusion on the stability of the thermal optima that this comparison aims to provide, remains conditional on the reconstructed temperature accurately reflecting the temperature that the foraminifera experienced. This is an important caveat that should be mentioned.

Importantly, I think there is another method to assess if the thermal response curves have changed. Shifts in the thermal optima of the magnitude like those suggested by the simulations (5-10 degC) would almost certainly lead to LGM assemblages that are compositionally different from those today, as the overlaps among the niches of the different species (groups) change. I have done some idealised simulations where I generated species counts based on thermal niches estimated from the same core top dataset as used in this study (ForCenS). When I shift the niches of the spinose species by 5-10 degC colder temperatures, I obtain assemblages of which 20-40 % lack good compositional analogues (depending on the magnitude of the shift), suggesting that the effect should be observable. Importantly, no-analogue assemblages in the datasets analysed by the authors are much rarer (~1% (Kucera et al. 2005)), seemingly arguing against large changes in the thermal niches (especially when keeping in mind that no-analogue assemblage could also result from other factors e.g. bioturbation, analytical uncertainty, environmental change). Thus, the authors need to reconcile their inferences with the lack of no-analogue assemblages during the LGM.

- Comparison with previous work

Third, the comparison with the work by Antell et al raises quite a few questions. The section seems a bit of an add-on, rather than an integral part of the study. For instance, why is it only included in the discussion section and why is it not analysed in a way that is comparable to the results of this study? Or why are these data not used to investigate the effect of the rate of change on the stability of the thermal optimum of the species? At the moment the reader is left with a poorly described analysis that yields a correlation that explains about 3% of the variance in the changes in the thermal optima. In my view, that does not amount to convincing support for the central conclusion of acclimatisation.

Some points that would need to be addressed are:
+ Why are the analyses not made comparable? Why are these data not split into ecogroups? Not all groups/species show changes in the thermal optimum after all (at least in the simulations). Alternatively, where do the data from the ForamEcoGENIE experiments plot in the crossplot (somewhere way off I presume because the simulated change in temperature is much larger)?
+ What do the data from Antell et al show for the LGM?
+ To the best of my knowledge Antell et al characterised the niche based on presence/absence data, whereas the approach by the authors relies on the abundance. That is a fundamental difference that at least needs to be mentioned and ideally needs to be explored.

- Methodological issues
I have also identified a few methodological issues that require clarification.

Ecogroups : The assignment of species to ecogroups is unclear and contains errors. Table S1 lists four ecogroups, but it is not clear how the so-called symbiont-facultative non-spinose group is treated in e.g. Fig 1. Is it excluded from the analysis (like with the modelling), or have the authors revised their grouping for the analysis? I think they are included, but assigned to other groups than indicated in the table, otherwise I cannot explain the presence of foraminifera at low temperatures (essentially N. pachyderma). L101-104 also seems to indicate that the grouping used in the analysis is different from what's shown in the table, but please explain.
I also quickly looked at some of the abundant species and was surprised to see N. pachyderma and N. incompta listed as symbiont facultative. To the best of my knowledge these two species are symbiont-barren (e.g. (Takagi et al. 2019)). In addition, T. quinqueloba is spinose, not non-spinose (as per the table and L102). So, please carefully check the table, clarify the discrepancies and update the analysis and table accordingly.

LGM compilation: I am confused as to why the authors merged these data products (L491). First of all, CLIMAP used a different age (18 ka) for the LGM than MARGO and EPILOG (19-23 ka). GLAMAP used both. Thus it needs to be explained why the data were merged, especially because of the climatically different conditions during Heinrich Stadial 1 immediately following the LGM (as defined by EPILOG/MARGO).
Second, MARGO postdates GLAMAP and EPILOG and the synthesis includes data from GLAMAP and EPILOG if they meet the selection criteria (Kucera et al. 2005). Please explain why the most recent (MARGO) compilation for the LGM was not simply used. Please also explain how datasets for which only relative abundances were available were treated. And finally, even though knit-picking, the taxonomy doesn't seem to be fully compliant with Brummer and Kucera (e.g. cultrata and eastropacia).

Characterisation of the thermal performance curves: the authors need to explain how they dealt with varying sampling effort in their characterisation of the thermal optima. Unequal sampling and analytical noise - as is the case for the sedimentary data - render it harder to accurately characterise the optima. The issue mostly applies to the sedimentary assemblages where the 95th percentile curves are wiggly

(and the definition of the optimum hard/uncertain), likely as a result of unequal sampling on the x-axis, but also varying number of specimens counted (y-axis). The authors should assess the influence of these effects (and hence to some degree the uncertainty of the optima), for instance by normalising the counts and bootstrapping.

Last, but not least, I missed where I can find the code used for the analysis. The authors have indicated where the model can be found and where the results/data are stored, but it's unclear where the code for the actual analysis can be found.

- Minor comments

L21: symbiont, add somewhere that this refers to photo-symbionts.
L31-34: all true, but what is the relationship to this study?
L52: "For instance, …" I don't understand the example here. That is not a reason why transfer functions are used and I think the statement applies to calcite only, not to all calcium carbonate.
L78: "two out of three", but the authors omit the results for the fourth ecogroup they have in their model.
L82: "retraction to" rather than "displacement"?
L98: if these changes in the optima are real, then the simulated absence of change must reflect compositional change because all species living at low temperatures seem to change their optima (but please clarify the ecogroups).
L119: "more than one optimal temperature" holds for every ecogroup, reword.
L146: is "unexpectedly" needed? I'm not sure what the (my) expectation would be. Same for "despite", not sure I follow.
L166-168: here is some hint at a potential mechanism, but details are absent.
L185: has fig 3a already been called?
L188: "The reanalysed optimal temperature ranges from -5 to +5 degC (Fig. 3b)" What is plotted in Fig 3b, the thermal optimum, or the change in the thermal optimum? I think the latter, but that is not consistent with the text. Also, the values are not consistent with the figure, which shows y-axis values that range from approximately -12 to 7 degC.
L189: it is not entirely clear to me what the purpose of Fig 3a really is. In the sentence before the authors quote Antell et al to say that the observed niche dissimilarieties are <20%, so why show this highly idealised example?
L428: which year(s) is/was used to define the pre-industrial?
L433: I assume the reanalysis data were used. If so, please specify accordingly (and see comment below).
L433: "seasonal blooms" odd wording for zooplankton, but more importantly, the proxy system models used by Tierney consider the seasonal recording of all sensors, not just the foraminifera. This needs to be specified to avoid giving the impression that the reconstruction really provides the temperature at the time when the foraminifera were most abundant. More importantly, the reanalysis product provides annual mean temperatures, not the seasonal temperatures.
L466: "other environmental drivers are also controlling", why only environmental? What about analytical noise and the possibility that the simulated temperature is not an accurate reflection of the

temperature that the foraminifera actually experienced (large differences in spatial and temporal grain between the simulations and fossil assemblages)?
Table S1: For quite a few species the presence or absence of symbionts is poorly constrained (probably part of the reason why the symbiont-facultative ecogroup is excluded from the analysis). So why set this trait in stone in this table? I think that sets a dangerous precedent and does not reflect our current (lack of) knowledge. I suggest that the authors clearly indicate which species have photo-symbionts, which don't and for which we don't know (and include a source where this information comes from). The assignment to the groups is then more interpretation than fact for the species where we lack knowledge on their symbiont ecology, which is fine as long as the authors explain their approach.

- References
Annan, James D., Julia C. Hargreaves, and Thorsten Mauritsen. 2022. "A New Global Surface Temperature Reconstruction for the Last Glacial Maximum." Climate of the Past 18 (8): 1883–96.
Kucera, Michal, Mara Weinelt, Thorsten Kiefer, Uwe Pflaumann, Angela Hayes, Martin Weinelt, Min-Te Chen, et al. 2005. "Reconstruction of Sea-Surface Temperatures from Assemblages of Planktonic Foraminifera: Multi-Technique Approach Based on Geographically Constrained Calibration Data Sets and Its Application to Glacial Atlantic and Pacific Oceans." Quaternary Science Reviews 24 (7-9): 951–98.
Takagi, Haruka, Katsunori Kimoto, Tetsuichi Fujiki, Hiroaki Saito, Christiane Schmidt, Michal Kucera, and Kazuyoshi Moriya. 2019. "Characterizing Photosymbiosis in Modern Planktonic Foraminifera." Biogeosciences 16 (17): 3377–96.

Referee #2 (Remarks to the Author):

This is a thought-provoking manuscript on zooplankton abundance change through time from past to the future. It's timely topic and I enjoyed my reading a lot. Manuscript is generally well written and model-fossil comparison is impressive. I have a few rather major issues that I like to see more explanation, disciussion, and/or analyses. But otherwise, I think it's a great job and I enthusiastically recommend to publish in Nature. Below are my comments. I hope they are useful to improve the manuscript.

Main points:
1. One issue is there are numerous studies using foram fauna for past SST reconstruction and they are basically well consistent with SST from climatic modeling and geochemical proxies (eg PRISM Pliocene Dowsett et al among others). This is kind of strong evidence for the niche conservatism of planktic forams. Also geochemical proxies and climatic models also have theoir own problems and uncertainties. I think you need more nuanced discussion. I tend to think your tone is a bit too strong. I think "niche conservatism of planktic forams" is not merely assumption.

Your results are exciting and very important ecologically and to think about past and future climatic impacts. So, I tend to think you may not need to say too strong against the usefulness of the transfer function.

Indeed, if you like to test usability of transfer function, it's better to use the same methods/units/etc as they use. I think they use %abundance for transfer function calibration. It can be different from your abundance, and better robust from such "acclimation". This is not really criticism to your discovery that is true exciting, but more to the framing of this story. I think it's better to tone down bit about transfer function critisism and more emphasize global change biology side. I really like this line of research as I wrote some review/opinion paper to advertise this line of paleobiological study in microfossils as Time Machine Biology (such as Yasuhara et al 2020, 2022, 2023). You may like to cite in the introduction to better emphasize "(paleo)biological story/context".

Dowsett, H. J., Robinson, M. M., Haywood, A. M., Hill, D. J., Dolan, A. M., Stoll, D. K., Chan, W., Abe-Ouchi, A., Chandler, M. A., Rosenbloom, N. A., Otto-Bliesner, B. L., Bragg, F. J., Lunt, D. J., Foley,K.M., Riesselman, C. R. (2012). Assessing confidence in Pliocene sea surface temperatures to evaluate predictive models. Nature Climate Change 2, 365–371.

Yasuhara, M., Huang, H.-H.M., Hull, P., Rillo, M.C., Condamine, F.L., Tittensor, D.P., Kučera, M., Costello, M.J., Finnegan, S., O'Dea, A., Hong, Y., Bonebrake, T.C., McKenzie, N.R., Doi, H., Wei, C.-L., Kubota, Y., Saupe, E.E., 2020. Time machine biology: Cross-timescale integration of ecology, evolution, and oceanography. Oceanography: 33(2), 16–28.

Yasuhara, M., Deutsch, C. A., 2022. Paleobiology provides glimpses of future ocean: Fossil records from tropical oceans predict biodiversity loss in a warmer world. Science: 375 (6576), 25–26.

Yasuhara, M., Deutsch, C. A.* 2023. Tropical biodiversity linked to polar climate. Nature: 614, 626–628.

2. It's good to explain what is "acclimation" exactly. It's different from wide niche or temperature range of a species? If there is a definition in ecology, good to cite such paper.

For example, we can say they acclimate to warmer temperature and so stayed in the same place/
But also we can say, they have wide thermal niche and so stayed in the same place.
"Optimum" may be shifted by acclimation but also by some other constraints of that particular latitudinal zone to prevent their move (such as ocean current system).
Would be good to add a bit of this kind of discussion.

3. The model considers surface production (food for them)?
Abundance could be related to surface production, and high productivity area can be different eg between LGM and pre-industrial.

4. You mention adaptation. You may like to refer to recent longer term foram studies in regard with this?

Fenton, I. S., Aze, T., Farnsworth, A., Valdes, P. & Saupe, E. E. Nature https://doi.org/10.1038/s41586-023-05712-6 (2023).

Woodhouse, A., Swain, A., Fagan, W. F., Fraass, A. J. & Lowery, C. M. Nature https://doi.org/10.1038/s41586-023-05694-5 (2023).

5. Finally and probably most importantly, a major issue is about fossil abundance. It's notoriously difficult to reconstruct.

Fig 1 says just "## for the panel b of fossil plots. What is it actually? Number of specimens per sample? Number of species per something? or some flux (specimens per year)?
If "per sample", it can change depending on how much sediment was processed.
So, we need more details on this kind (I think it's not detailed in the methods).

More importantly, forams dissolve during sinking after their death, so foram abundance in surface sediment (or LGM sediment section) are not same as your modelled one or real living foram abundance in water column. Then, dissolution is stronger in deeper water depths, especially below lysocline or CCD.
So, I suspect your fossil performance curves are largely affected by depth differences among samples.
Also, if a site was near to shore (coastal, continental margin etc), you will have more lithic grains and so foram abundance is lower by such dilution by more sediment grains.
Diversity, %abundance etc are more robust for these variations, but absolute abundance could be directly affected by these.
I think it's good to address these issues somehow.
A possibility is to plot Fig1b-ish by using only pelagic sites shallower than lysocline or CCD?
Or, some calibration may be possible with depth like the opposite of estimating particulate organic carbon flux from surface production. and/or at least some discussion on this issue.

Specific comments:
(would be great to have line and page numbers)

1. "To avoid extinction, marine taxa have shifted their habitat to more
suitable environments3–6"

These are some of the latest relevant papers.
Will be good to cite.

Chaudhary, C., Richardson, A.J., Schoeman, D.S., Costello, M.J. (2021). Global warming is causing a more pronounced dip in marine species richness around the equator. Proceedings of the National Academy of Sciences, 118(15), e2015094118.

Yasuhara, M., Deutsch, C. A., 2022. Paleobiology provides glimpses of future ocean: Fossil records from tropical oceans predict biodiversity loss in a warmer world. Science: 375 (6576), 25–26.

Yasuhara, M., Wei, C.-L., Kucera, M., Costello, M.J., Tittensor, D.P., Kiessling, W., Bonebrake, T.C., Tabor, C.R., Feng, R., Baselga, A., Kretschmer, K., Kusumoto, B., Kubota, Y., 2020. Past and future decline of tropical pelagic biodiversity. Proceedings of the National Academy of Sciences of the United States of America: 117, 12891–12896.

2. Phenotypic plasticity = acclimation? It can also be broad niche?

3. “estimating plankton extinction risk11”
May like to add Yasuhara et al 2012:
Yasuhara, M., Hunt, G., Dowsett, H. J., Robinson, M. M., Stoll, D. K., 2012. Latitudinal species diversity gradient of marine zooplankton for the last three million years. Ecology Letters, 15: 1174–1179.

4. “distributional shifts12,13”
May like to add Yasuhara et al 2020:
Yasuhara, M., Wei, C.-L., Kucera, M., Costello, M.J., Tittensor, D.P., Kiessling, W., Bonebrake, T.C., Tabor, C.R., Feng, R., Baselga, A., Kretschmer, K., Kusumoto, B., Kubota, Y., 2020. Past and future decline of tropical pelagic biodiversity. Proceedings of the National Academy of Sciences of the United States of America: 117, 12891–12896.

5. “based on the assumption that fossil assemblages have the same thermal
preference as modern assemblages”
You may like to use a term for this in ecology, niche conservatism.
Also this paper discussed relevant issue in details and so you may like to cite:
Yasuhara, M., Tittensor, D. P., Hillebrand, H. and Worm, B., 2017. Combining marine macroecology and palaeoecology in understanding biodiversity: microfossils as a model. Biological Reviews: 92, 199–215.

6. “Each ecogroup's thermal performance is flexible and depends on the interaction between the ecogoup’s set of functional traits (size, spine, symbiont) and abiotic (temperature, nutrient, light) and biotic environmental conditions (resource competition and grazing pressure from higher trophic levels)”
“Thermal performance depends on temperature” sounds bit circular?
Also this sentence is bit complicated and not sure if “the interaction between” is for all of traits, abiotic factors and biotic factors or only one/some of them.
“biotic environmental conditions” is better to be biotic factors?
So, good to rephrase.

7. “These results agree with previous studies that warm-water species' optimal niche (i.e., a subset of niche where they exhibit the highest fitness) has greater variability19”
Indeed, Yasuhara et al 2012 showed higher variation in temperature-diversity relationship in the tropics than higher latitude. This is interestingly consistent and you may like to cite.

Yasuhara, M., Hunt, G., Dowsett, H. J., Robinson, M. M., Stoll, D. K., 2012. Latitudinal species diversity gradient of marine zooplankton for the last three million years. Ecology Letters, 15: 1174–1179.

8. “Unexpectedly, the modelled future foraminifera do not acclimate as much as in the deglacial warming (Fig. 2) despite experiencing a lower warming (1-4 °C compared to 6 °C).”
Why “Unexpectedly”? For me it rather makes sense. On-going warming is much more rapid and so no time for acclimation?

9. “Discussion” section. Better to rename? Because, you already “discussed” a lot before this section.

10. ”In contrast to previous studies which suggest a conservative planktic foraminifera niche18,19,34, our results reveal that foraminifera can shift their thermal niche and acclimate to geological warming."
I don’t disagree but I think it’s better to be cautious, because previous studies discussed with species range or relative% abundance for transfer function, instead of absolute abundance (and their peak). Thermal niche may not be very shifted in the sense of species temperature range?
I think you may like to add a bit more nuanced discussion.

11. “However, by reanalysing the species optimal temperature in Antell et al.18, we opposingly find a significant relationship (p =0.0086; Fig. 3b)”
Why? Their miscalculation?

12. “However, such acclimation capacity gives
rise to uncertainty in several fixed niche-based approaches. The modern assemblage of foraminifera has been used to reconstruct glacial surface ocean temperature36.”
I think you need a bit cautious discussion, because such approaches use NOT abundance peaks like this study but use more range, % abundance, etc that could be more robust and stable?

13. “This disparity might come from the fact that the increasing ocean temperature is approaching the maximum tolerable temperature for the current foraminifera trait set in the model.”
It makes sense because for the late Quaternary after the MPT glacial periods are much longer than interglacials, and so they could be more adapted cold-ish environment as discussed in Yasuhara et al 2020 PNAS (and warm-adapted species were gone by post-Pliocene cooling, as seen in Yasuhara et al 2012 Ecology letters. Also see Rillo et al 2022).

Rillo, M. C., S. Woolley, & H. Hillebrand, (2022) Drivers of global pre-industrial patterns of species turnover in planktonic foraminifera, Ecography 2022(1)

14 “in abrupt events such as the Heinrich cooling event at 16.8 ka, the onset of Holocene at 11 ka40, the modern warming27, and our future projections”

The onset of Holocene is an abrupt event?

Sincerely yours,
Moriaki Yasuhara (this is signed review)

Referee #3 (Remarks to the Author):

The manuscript by Ying et al. applies fossil data and modeling to assess the potential of foraminifera to acclimate to temperature change. The study contrasts the thermal preference of foraminifera between the Last Glacial Maximum and pre-industrial era to the projected response under future warming scenarios. The manuscript is also a response to previous work that had a seemingly opposite conclusion from geological data - namely that planktonic foraminifera did not change their thermal tolerances in response to global temperature change. Here, the authors reevaluate the same data to suggest that there is evidence that particular groups of foraminifera (particularly the spinose taxa) did change their thermal preferences between the LGM and pre-industrial. This re-evaluation of fossil data is consistent with the authors' new modeling results. The authors apply an intermediate complexity Earth system model (cGENIE) that includes a mechanistic plankton model (ForamEcoGENIE). The LGM and pre-industrial model experiments are run as two equilibrium climate states and the thermal preferences of three different foraminiferal ecogroups are assessed. The model indicates no change between the LGM and pre-industrial thermal tolerances of the symbiont-barren non-spinose taxa, but the other two groups (symbiont-barren spinose and symbiont-oligate spinose) both show different thermal preferences between the LGM and preindustrial. This result suggests that foraminifera may be able to acclimate to changing climate. However, the authors then use the same model to simulate a range of warming scenarios over the next century and show that the modeled foram ecogroups do not acclimate - thermal preferences are virtually indistinguishable from the pre-industrial simulation. Thus foraminifera migrate poleward, but at the same time, the total modeled foraminiferal biomass declines.

These are interesting and important results and I think the paper is appropriate for Nature. I think that there are aspects of the methods that can be clarified for greater accessibility (below), but my most substantive suggestion regards the treatment of equilibrium versus transient simulations. Specifically, Line 226-230: "This disparity might come from the fact that the increasing ocean temperature is approaching the maximum tolerance temperature for the current foraminifera trait set in the model. Alternatively, it could arise from the difference between the LGM equilibrium experiment and a future abrupt warming where environmental changes are different and still developing." I think that this distinction is very important. In other words, is there some maximum amount of warming where acclimation cannot occur regardless of the rate of temperature change? Or conversely, would the modeled thermal preferences ultimately shift in response to equilibrium warming with the shift proportional to the total warming? From a modeling framework, it should be straightforward to test these two possibilities. If the authors would run equilibrium climate scenarios (10,000 year duration comparable to the LGM and PI states) with 1.5-2-3-4°C warming imposed (using the pre-industrial configuration) then they would have a like-for-like comparison to the LGM equilibrium experiment and can disentangle the relative role of the temperature change itself as opposed to the rate of change.

The description of the ForamEcoGENIE model in the Methods section could be improved for clarify. For instance, differences between EcoGENIE and ForamEcoGENIE are not fully described. There are minor issues: the cGENIE grid is not uniformly 10° latitude resolution (uniform in the sine of latitude), only temperature, salinity, Fe and P mentioned as tracers (also, it's a bit odd to include the conservative tracers (T,S) as biogeochemical tracers, which are more typically understood as passive tracers, in the sense that they do not impact the modeled physical circulation), physical and chemical conditions are described as 'used by EcoGENIE' but how/if the modeled plankton influence biogeochemical tracers is not described. What are the controls on modeled NPP? Is there a way to separate the relative impact of temperature and NPP change on the decline in foraminiferal biomass? Finally, the Methods describes improvements made to ForamEcoGENIE from a previous publication, but it is not clear how these improvements were calibrated against observations (what observations?) or what impact these improvements have to the particular experiments detailed here.

The figures are very nice, but I think it would help to add vertical lines in Figure 2b to show the lack of a shift in the thermal preference in the future warming scenarios (as in Fig 1).

Author Rebuttals to Initial Comments:

Response to the Review Comments

Overview

We sincerely thank our reviewers' constructive comments that help improve our study, and the editor for giving us time to conduct these changes. The main changes are summarised in the following table. We next provide a one-by-one response, with our response in blue.

Major points of concerns	Related changes
The LGM/PI observational data analysis (Reviewers #1/2)	1.To address the concerns about the impact of foraminiferal samples on determining thermal performance curves, we have replaced absolute abundance with relative abundance in all our LGM/PI data analyses. This change has allowed us to rely on more available data, improving our estimate robustness but not changing the results. To address the questions of age, dissolution etc, we now only rely on the curated MARGO data that has undergone quality control, strict age control, and sedimentation rates that are high enough to avoid potential mixing effects (Lines 409).<br>2.To better characterise optimal temperature for species with broad environmental preferences, we now identify the temperature range that exceeds 50% maximum relative abundance and provide its mean/max/min/std.<br>3.To quantify the importance of sampling efforts, we have improved our assessment of sampling uncertainty by using a range of regression models (90-99th quantile) instead of only relying on the 95th percentile (Lines 468).<br>4. The updated optimal temperatures agree well with previous estimates in the literature based on maximum body size (provided in the Extended data table 1) (Schmidt et al. 2004 Marine Micropaleontology)
The interpretation of acclimation (Reviewers #1/2)	We have clarified the definition of niche and acclimation, and discussed other potential drivers, such as dispersal, evolution, vertical migration, and replacement of species in the assemblage [Lines 118-133].
The mechanistic insights (Reviewers #1/3)	We have added a new section (and a new Figure 4) to determine the potential drivers for the observed thermal acclimation. We conclude that the change in the emergent niche is primarily facilitated by food availability.
The conflict with previous study (Reviewers #1/2)	1. We have expanded on this aspect. We now introduce the Antell et al. (2021)'s method and results and argue that optimal and total niche respond differently. We also show that the optimal temperature in Antell et al. (2021) present the same trend as our abundance-based data for both the LGM/Holocene and longer time.<br>2. As suggestion by reviewer#2, we removed the introduction/discussion on the transfer functions as they are based on relative abundances across the total niche, while our approach focuses on the optimal niche.<br>3. Finally, we have followed the reviewer #1's recommendation and employed a sensitivity analysis (Line 529) and a bootstrapping method (Line 526) to determine the sampling uncertainty.
Others	1. All codes of analysis and visualisation are now provided in https://github.com/ruiying-ocean/lgm_foram_niche.<br>2. A new author (Malin Ödalen) has been added to the manuscript for her contribution to the LGM cGENIE configuration, on which our foraminifera model is based. She reviewed our final draft.<br>3. The manuscript has been formatted following Nature's instructions; the figure numbering has been modified from the original manuscript.<br>4. The title now is "Marine zooplankton acclimated to warming in the geological record face limits by the next century".

Referee #1 (Remarks to the Author):

- Acclimatisation

My first comment relates to the concept of acclimatisation and whether or not the results unambiguously point to plasticity in the environmental niche or rather to different parts of the fundamental niche being occupied. This distinction between the fundamental niche (the environmental space that can theoretically be occupied) and the realised niche (sometimes called habitat: the part of the fundamental niche that is occupied) is important. ... I think the authors can only infer the realised niche from their simulations and they should make this clearer in the text…

[Response] The reviewer's comment highlights the issues related to our lack of definition for the niche, acclimation, and the relationship between them. We now **clearly define these key aspects in the revised manuscript:**
Line 76: " The model outputs represent the realised niche of foraminifera ..."
Line 120-123: "We define thermal acclimation as the non-evolutionary reversible responses of some foraminifera to maintain performance across a range of environmental conditions as acclimation, following the Sixth Assessment Report of the United Nations Intergovernmental Panel on Climate Change (IPCC)[31]"
Line54-57: "... because adaptation and acclimation generate new genotype and phenotype with novel fundamental niche (the theoretical liveable condition) and realised niche (the observed living condition), respectively"

If the changes in the thermal optima are really reflected in the fossil data (see below), then the authors need to discuss these in the light of this distinction between fundamental and realised niche and argue why the observations point to acclimatisation. To address this issue the authors need to provide more careful and precise wording. The clearest case where I have my doubts is in the warm-water species (symbiont-obligate spinose): temperatures above 25 degC are simply absent from the LGM simulation/reconstruction. The emergence of new (warm-water) habitats during the Holocene ia associated with a shift in the thermal optimum, but does not require physiological changes in the organisms as it could simply reflect the colonisation of these new habitats. Similarly, changes in the symbiont-barren species could also simply be due to a different portion of the fundamental niche being occupied as a result of changing (a)biotic interactions.

[Response] We now **discuss the different causes for the observed realised niche shift** and justify why we attribute the result as thermal acclimation (Lines 118-133):
"We interpret the realistic niche shift of some foraminifera towards warmer environments, coupled with their maintained/increased abundance during the deglaciation, as thermal acclimation. ... The realised niche shift can have other multiple causes, including adaptation, dispersal limitation, species replacement, and a lack of climate niche in the LGM (Extended Data Fig. 4). However, these hypothetical causes are not supported by current evidence. Neither morphological nor genetic data suggests the emergence of a new species since the LGM[20]. A global gene flows of foraminifera[21] suggests the lack of dispersal limitation. Besides, the species replacement is greatest in the transitional regions (40 °N/S) but minimal in the low latitudes[22] (Extended Data Fig. 5), contrasting with our niche shift finding. Finally, while the deglacial warming introduces a new warm temperature range for symbiont-obligate foraminifera, it cannot explain their rising abundance in the 25-30 °C and dropping abundance in the 15-20 °C. This pattern, however, supports our interpretation of thermal acclimation

In addition, it remains unclear what temperature (at which depth and season) is used to characterise the niche (in both the simulation and the reconstruction)

[Response] We now **detail the used temperature products** including the Tierney et al. (2020)'s data assimilation and the HadISST data (Lines 440-449):
"We use the geographical information in the abundance dataset to look up the sea surface temperature in the nearest grid of data product. We use HadISST1 dataset[63] (1×1°, latitude × longitude, 1870-1900 annual mean climatology) as our pre-industrial temperature reference and

the Tierney et al.[64] data assimilation (1.9 × 2.5°, latitude × longitude) as our LGM reference. Tierney et al.[64] used an Ensemble Kalman filter to incorporate the information of geochemical proxy data compilation (19-23 ka) with the constraints of a climate model (iCESM). The proxy compilation includes organic chemistry-based proxies ($U_{37}^{K'}$, TEX_{86}), and foraminifera shell-based $\delta^{18}O$ and Mg/Ca. Each type of proxy was calibrated used a Bayesian model to propagate proxy uncertainties and seasonal bias. We do not use assemblage-based temperature reconstruction in the MARGO to avoid circular reasoning."

... and the authors should comment on the possibility that the temperature plotted on the x-axis in figure 1 was not exactly the temperature at which the foraminifera live
[Response] We address this now in Lines 80-83 and Lines 451-453:
"This reconstructed thermal performance curve represents the global long-term steady state and does not reflect the in situ living environments of foraminifera due to model resolution, uncertainty in proxy temperature reconstruction data, and dynamic changes in species vertical habitats"

It is worth noting that the temperature data used here only represents the surface layer (0.5 m for LGM data and 0.2 m for HadISST) and its long-term climatology, therefore not indicating the in situ temperature of the precise habitat. ..."

... could, for instance, a different seasonal temperature range during the LGM explain part of the change in the optima?
[Response] As suggested, we have **assessed the seasonal temperature range during the LGM/PI in the cGENIE model** (Fig. R1). The model shows that the seasonal temperature change is strongest in the high latitudes and weakest in the low latitudes. Comparing the LGM and PI results shows that the seasonality barely changed in the low latitudes where our acclimation result is the most significant. Therefore, the impact of changed seasonal temperature range should be negligible for our thermal optima shift (Line 460).

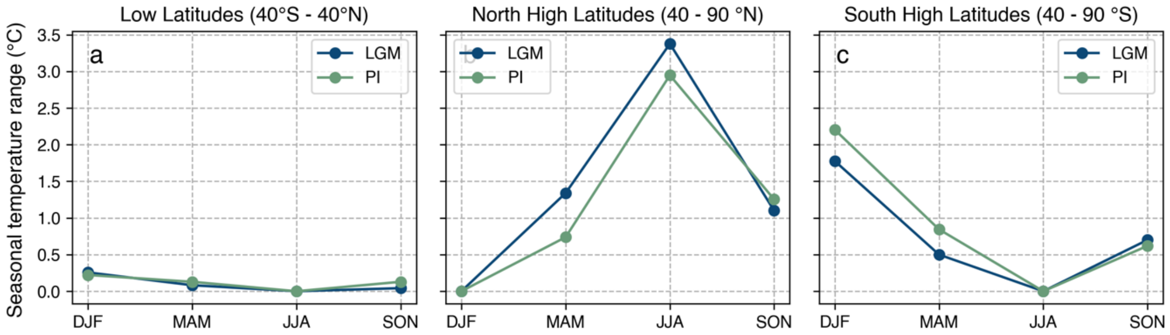

Fig. R1. cGENIE-modelled seasonal cycles of LGM and PI mean sea surface temperature. We average the temperature for each latitudinal band and calculate the seasonal mean temperature. For comparison, we normalise the absolute temperature to the seasonal temperature range. The seasonal variability is weakest in (a) low latitudes (40 °S - 40 °N) and strongest in (b) high latitudes in the North Hemisphere (40 - 90 °N), with (c) high latitudes in the Southern Hemisphere (40 - 90 °S) in the middle.

- Mechanism and relevance
My second comment is related to the first, but focuses on the mechanism that could be responsible for the change in the optima. … This means that the authors should be able to dig deeper into the model output and point at the mechanism that drives the changes in the thermal performance. …
[Response] We followed the suggestion and **investigated the foraminifera ecology in the model. This results in a new section** (starting from Line 244). We analysed how different processes can cause the thermal niche change (Lines 245-255 and supplementary method L111). Our analysis suggests that the changing food availability and foraminiferal assimilation efficiency are the main contributor, while the grazing pressure of foram is low and the respiration/morality has same/zero thermal dependency in different climate states. Because the food availability can be represented by the net primary productivity (NPP), we plotted the NPP

value along SST gradient (Fig. 4a). We found that the NPP trend along SST gradient was more stable in high latitudes from the LGM to PI and future, which explains the niche conservatism of symbiont-barren group living in these regions. We also plotted the carbon to phosphate biomass ratio of each foram group (Fig. 4b-d) because the modelled assimilation efficiency is determined by nutrient quota. This shows that symbiont-obligate foraminifera can flexibly acclimate to local trophic condition based on variable stoichiometry, aligning with some observations (L264-275).

The suggestion the authors make is that the difference between the simulated response for the LGM and the future is in part related to the pace of environmental change: slow during the deglaciation and fast into the future … This may be true, but they lack the data to conclude this. They ran time slice (equilibrium) simulations for the LGM and the PI and thus, by definition, cannot make any statements about the speed (or the timing, for that matter, e.g. "during the last deglacial warming" L24-25) of the apparent acclimatisation. …. they should investigate alternative explanations for their observations, because there must be other reasons since they show up in the equilibrium simulations.
[Response] Our investigation of model results (Fig. 4) now provides an alternative explanation (food supply) of different responses from past to future and can support our previous argument that timescale is important for acclimation. The key driver NPP involves the nutrient distribution, ocean circulation and dust deposition. Although these climatic changes are directly parameterised in the equilibrium model, in reality they require long time to form and establish (L277-287) (related discussion can also be found in the responses to reviewer#3).

"The NPP-SST relationship also explains the difference in response between LGM and future conditions.... While the global temperature rise is similar from the LGM to pre-industrial and from today to 2100, the former experiences more pronounced forcings including changes in ocean circulation, the iron cycle, and wind stress pattern[37]. According to the Milankovitch theory, these changes are triggered by external astronomical forcings and require longer time to establish and stabilise, allowing the observed acclimation. In contrast, for abrupt events such as the Heinrich cooling event at 16.8 ka, the end of Young Dryas[38], historical warming[25,26], the foraminiferal assemblages in the observation mostly migrated instead of acclimated (Fig. 3)."

.. It is however unclear to me how representative the results are for other plankton and I am surprised that this is not explored in the simulations. The authors could assess in their simulations how representative planktonic foraminifera are for phyto/zooplankton and/or connect better with the literature to demonstrate that the plasticity implied for foraminifera also characterises other plankton. .. In short, the study needs to provide more convincing arguments that the findings for planktonic foraminifera have bearings beyond this group.
[Response] We appreciate the reviewer's suggestion to extend our foram results to other plankton groups. However, the strength of this study is to use the fossil record to support the modelling. Whilst we could infer results about other plankton types from our model results, we would not be able to corroborate them in the same way because few other groups have such an excellent fossil record as foraminifers do. In addition, many of these fossil groups (diatom, dinoflagellates) have strong taxonomic biases resulting in geographic areas with little or no data or less clear understanding of biomass. Therefore, we have **removed the implication of our results on other plankton** but reviewed previous literatures that lead to the similar conclusion based on a standalone adaptive phytoplankton model (Line 313).

Comparison with observations
Whilst my first two comments concerned the interpretation of the results, I am also not yet convinced that the fossil record really supports the conclusions from the modelling. This is partly due to methodological issues that render it difficult to characterise the thermal optima (see below) and the fact that all species including the symbiont-barren non-spinose show apparent changes in the niche (Fig. S3) even though the simulations suggest otherwise (Fig. 1).
[Response] To **improve the observational results**, we have made the following changes some of which are summarised on page one but briefly repeated here. Firstly, we now use only relative abundance to determine the thermal optima (in both observations and the model; Line 412, Line

400-404). Secondly, we have constrained our data to the MARGO database to ensure better data quality control (Line 408). Additionally, we replace the original single point optimal temperature with a range of optimal temperatures where species are present with half or more of their maximum abundance and provide the mean/min/max/std of this optimal range (Line 78 and Line 479). All these changes have resulted in a smaller magnitude of thermal optima changes (averagely 2 degrees), and not all species experience niche change (Fig. 1; Extended Data Figure 3), thus addressing the reviewer's concern. The updated results still compare well with the model and previous body size-based observations (Schmidt et al. 2004).

Moreover, the conclusion that the changes are real ultimately depends on how well the reconstructed temperatures represent those where and when the foraminifera lived. Since the reconstruction that the authors used is based on a single climate model and has a spatial correlation structure that is essentially the same as in that model, it remains controversial (e.g. (Annan, Hargreaves, and Mauritsen 2022)). This means that the support for the conclusion on the stability of the thermal optima that this comparison aims to provide, remains conditional on the reconstructed temperature accurately reflecting the temperature that the foraminifera experienced. This is an important caveat that should be mentioned.
[Response] We now **highlight the influence of the precision of the underlying temperature reconstruction** on our thermal optima (Lines 461-465):

"However, the LGM climate reconstruction is still an actively developing topic. Although we include multiple realisations of the modelled LGM climate (cGENIE and HadCM3 as below) and proxy-based temperature (Tierney et al.[64]), the thermal optima in our study are ultimately conditional to the fidelity of these reconstructed climate states"

.. Shifts in the thermal optima of the magnitude like those suggested by the simulations (5-10 degC) would almost certainly lead to LGM assemblages that are compositionally different from those today, as the overlaps among the niches of the different species (groups) change. ..Importantly, no-analogue assemblages in the datasets analysed by the authors are much rarer (~1% (Kucera et al. 2005)), seemingly arguing against large changes in the thermal niches (especially when keeping in mind that no-analogue assemblage could also result from other factors e.g. bioturbation, analytical uncertainty, environmental change). Thus, the authors need to reconcile their inferences with the lack of no-analogue assemblages during the LGM.
[Response] We thank the reviewer for providing another method to improve our assessment of the thermal niche. Firstly, **we have updated our analysis to address this issue of sampling uncertainty** (see above). The updated average niche shift of all species is around 2 degrees (Extend Data Table 1) when based on the larger sample set and relative abundance. Secondly, we have **emphasised the difference between optimal and overall niche** (see the major comment below). While we report the on average 2°C change of the optimal niche, the shift of overall niche might be less and therefore causing fewer novel assemblages.

Finally, reviewer #1 suggests that the acclimation will cause non-analogue assemblage. However, a small number of symbiont-obligate species (*G. ruber, T. sacculifer*) show the largest niche changes and dominate the (sub)tropics in both the LGM and modern. This finding is highlighting that the overall composition barely changed (at least not detectable as non-analogue assemblage; Jonkers et al. 2023, Nature Geosciences) but the temperatures leading to highest abundance did. Therefore, the acclimation does not necessarily lead to a non-analogue assemblage. It is also not clear which percentage changes within an assemblages would be needed to generate a non-analogue and be outside the error of the methodology (or noise).

- Comparison with previous work
Third, the comparison with the work by Antell et al raises quite a few questions. The section seems a bit of an add-on, rather than an integral part of the study. … explains about 3% of the variance in the changes in the thermal optima. In my view, that does not amount to convincing support for the central conclusion of acclimatisation.

Some points that would need to be addressed are:
+ Why are the analyses not made comparable? Why are these data not split into ecogroups? Not all groups/species show changes in the thermal optimum after all (at least in the simulations). Alternatively, where do the data from the ForamEcoGENIE experiments plot in the crossplot (somewhere way off I presume because the simulated change in temperature is much larger)?
+ What do the data from Antell et al show for the LGM?
+ To the best of my knowledge Antell et al characterised the niche based on presence/absence data, whereas the approach by the authors relies on the abundance. That is a fundamental difference that at least needs to be mentioned and ideally needs to be explored.
[Response] We have expanded this topic and **added a separate section dedicated to how our study compares with Antell et al. (2021)** (Lines 146-169). This should make the content clearer and more integral.

We have highlighted the challenges to do an in-depth comparison with Antell et al. (2021) (Line 160-162 and Line 505-510). Firstly, our foraminifera model/data is based on abundance rather than presence-absence. Additionally, our LGM proxy data are mostly SST and differ from the depth-resolved model used in Antell et al. (2021) as depth adaptation is dynamic and difficult to assess without species- and location-specific geochemical assessment of depth. The most important point is that the data presented in Antell et al. (2021) have a coarser spatial-temporal resolution and less data quality control than the MARGO dataset.

Below is the requested figure that visualises the presence-based LGM/Holocene foram niche (Fig. R2). It is consistent with our abundance-based results: during the Holocene (last 8 kyrs) temperatures for the optimal niche (in diamond mark) are higher than during the LGM. Note that the data also suggests that there were changes in ranges which suggest that non analogue composition may have existed, though this is not the focus of this study.

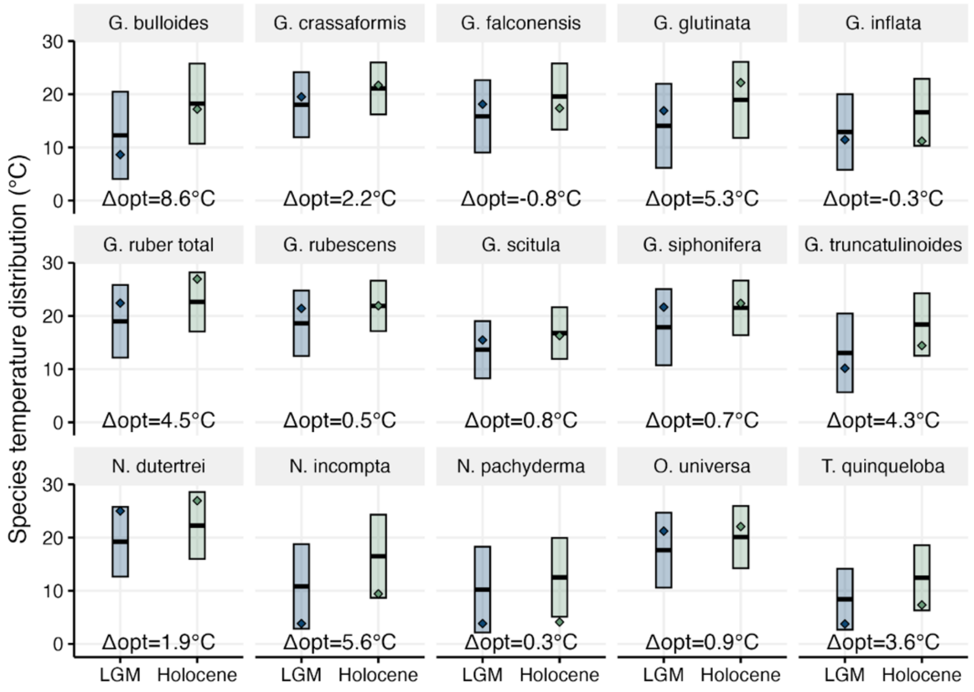

Fig. R2. The species thermal niche shown in the LGM and Holocene subset of Antell et al. (2021) data. The crossbar stands for the mean temperature (thick black line) and standard deviation. The optimal temperature is marked by diamond, with the annotation of change (Holocene-LGM). The original data: Antell et al. (2021).

As for the little explained variance in this data, it is independent from the significance. In Lines 530-540, we have tested for bigger minimal sample sizes to build the probability density function

to improve the estimate of thermal niche (which links to the sampling effort concern in next comment). The linear model kept the same significance ($P < 0.05$; Supplementary Figure 5).

- Methodological issues

I have also identified a few methodological issues that require clarification.

Ecogroups: The assignment of species to ecogroups is unclear and contains errors. Table S1 lists four ecogroups, but it is not clear how the so-called symbiont-facultative non-spinose group is treated in e.g. Fig 1. .. L101-104 also seems to indicate that the grouping used in the analysis is different from what's shown in the table, but please explain.

.. So, please carefully check the table, clarify the discrepancies and update the analysis and table accordingly.

[Response] We have improved the methodological description here, including how we group species and the underlying references (Line 427-436). We have also clarified that we only report three well-established ecogroups (symbiont-barren non-spinose, symbiont-barren spinose, symbiont-obligate spinose) as the biological understanding of what causes acquisition or loss of symbionts in facultative symbiont bearing foraminifera is limited, hindering us to define costs and benefits (Lines L435). In addition, we have introduced more examples of species for foram experts' interests (Line 104 and Line 437). Finally, we carefully reviewed and have updated the table and all the related analyses.

LGM compilation: I am confused as to why the authors merged these data products (L491). ..And finally, even though knit-picking, the taxonomy doesn't seem to be fully compliant with Brummer and Kucera (e.g. cultrata and eastropacia).

[Response] We have improved the method and its description here, including the use of relative abundance only in the MARGO (L408-416), and the standardisation of taxonomy (L418-425). The Brummer and Kucera (2022) did use *G. cultrata* and *G. eastropacia* to replace *G. menardii* and *G. theyeri.* Description in mikrotax:

- *G. eastropacia page: G. theyeri and G. eastropacia are widely accepted to be synonyms (e.g by Kennett & Srinivasan 1983), and the taxon has usually been named G. theyeri. However, Brummer & Kucera (2022) showed that G. eastropacia has priority (publishedin Jan 1974 vs April 1974) and so is the correct name. (last access: Nov 19, 2023).*
- *G. menardii page: Brummer & Kucera (2022) present a long, detailed and well-argued case that the correct name for this taxon is G. cultrata (last access: Nov 19, 2023).*

Characterisation of the thermal performance curves: the authors need to explain how they dealt with varying **sampling effort** in their characterisation of the thermal optima. Unequal sampling and analytical noise - as is the case for the sedimentary data - render it harder to accurately characterise the optima. The authors should assess the influence of these effects (and hence to some degree the uncertainty of the optima), for instance by normalising the counts and bootstrapping.

[Response]The rendering of the thermal performance curves has been updated and the temperature uncertainty has been emphasised (see above comments). In addition to the use of MARGO and relative abundance data, we additionally addressed the sampling uncertainty by

1. using ten different quantile levels, as explained in the L472-477

"the fitted maximum abundances of foraminifera have higher uncertainties in those under-sampled regions. To quantify such uncertainty, we apply ten different models with quantile levels from 90_{th} to 99_{th} and calculate their mean and standard deviation. The resulted standard deviation that measures the sensitivities of the models to the outlier values could provide the assess of sampling effort in this region"

2. showing the spatial sampling efforts (Supplementary Figure 4).
3. for the data reanalysis, bootstrapping the linear regression model as suggested, combined with a sensitivity analysis (Line 529; Supplementary Fig 5) to assess the sampling uncertainty in Antell et al. (2021) data.

Last, but not least, I missed where I can find the code used for the analysis. The authors have indicated where the model can be found and where the results/data are stored, but it's unclear where the code for the actual analysis can be found.

[Response] These codes and related instruction are uploaded to https://github.com/ruiying-ocean/lgm_foram_niche. This has been added to the Code Availability section of the revised manuscript.

- Minor comments
Minor comments have been implemented, some sentences removed, and only selected examples where explanation is warranted are in the below response.

L31-34: all true, but what is the relationship to this study?
We remove the sentence.
L428: which year(s) is/was used to define the pre-industrial?
We define this as 1870-1900.
L433: I assume the reanalysis data were used. If so, please specify accordingly (and see comment below).
Yes, we clarify this by using data assimilation.
L466: "other environmental drivers are also controlling", why only environmental? What about analytical noise and the possibility that the simulated temperature is not an accurate reflection of the temperature that the foraminifera actually experienced (large differences in spatial and temporal grain between the simulations and fossil assemblages)?
We remove these in the method and mention the sampling noises in Line 160.
Table S1: For quite a few species the presence or absence of symbionts is poorly constrained (probably part of the reason why the symbiont-facultative ecogroup is excluded from the analysis). So why set this trait in stone in this table?
We update this table to reflect the concerns and refer to published assignments.

Referee #2 (Remarks to the Author):
Main points:
1. One issue is there are numerous studies using foram fauna for past SST reconstruction and they are basically well consistent with SST from climatic modeling and geochemical proxies (eg PRISM Pliocene Dowsett et al among others). This is kind of strong evidence for the niche conservatism of planktic forams. Also geochemical proxies and climatic models also have theoir own problems and uncertainties. I think you need more nuanced discussion. … if you like to test usability of transfer function, it's better to use the same ethods/units/etc as they use. ... I think it's better to tone down bit about transfer function critisism and more emphasize global change biology side
[Response] We follow the suggestion and rebalance the discussion staying close to our main focuses in acclimation and remove the discussion on transfer function.

2. It's good to explain what "acclimation" is exactly. …. For example, we can say they acclimate to warmer temperature and so stayed in the same place/. But also we can say, they have wide thermal niche and so stayed in the same place. "Optimum" may be shifted by acclimation but also by some other constraints of that particular latitudinal zone to prevent their move (such as ocean current system).
[Response] We now define acclimation and address the niche shift in the context of acclimation (see the first major comment of reviewer #1)

3. The model considers surface production (food for them)? Abundance could be related to surface production, and high productivity area can be different eg between LGM and pre-industrial.
[Response] Yes, it does. We have clarified this point in a new section and a new plot (Fig. 4 see discussion in earlier response to reviewer 1).

4. You mention adaptation. You may like to refer to recent longer term foram studies in regard with this?
Fenton, et al. Nature (2023). Woodhouse, et al. Nature (2023).

[Response] Thank you for the suggestion. Unfortunately, due to limited space in the reference list, experimental evidence is preferred over macroevolutionary studies in the context of introducing the plankton adaptive capacity.

5. Finally and probably most importantly, a major issue is about fossil abundance. It's notoriously difficult to reconstruct.
Fig 1 says just "## for the panel b of fossil plots. What is it actually? Number of specimens per sample? Number of species per something? or some flux (specimens per year)?
If "per sample", it can change depending on how much sediment was processed…
More importantly, forams dissolve during sinking after their death, so foram abundance in surface sediment (or LGM sediment section) are not same as your modelled one or real living foram abundance in water column. ….o, I suspect your fossil performance curves are largely affected by depth differences among samples.
Also, if a site was near to shore (coastal, continental margin etc), you will have more lithic grains and so foram abundance is lower by such dilution by more sediment grains.
Diversity, %abundance etc are more robust for these variations, but absolute abundance could be directly affected by these.
A possibility is to plot Fig1b-ish by using only pelagic sites shallower than lysocline or CCD?
Or, some calibration may be possible with depth like the opposite of estimating particulate organic carbon flux from surface production. and/or at least some discussion on this issue.
[Response] We have updated this analysis related to this part (see response to the reviewer#1). We now use relative abundance data and use the quality-controlled Margo database to avoid dissolution impact (Line 408).

Specific comments:
(would be great to have line and page numbers)
[Response] Done.

1. "To avoid extinction, marine taxa have shifted their habitat to more suitable environments3–6"
These are some of the latest relevant papers. Will be good to cite.
Chaudhary, C., et al. 2021. PNAS, 118(15), e2015094118.
Yasuhara, M., Deutsch, C. A., 2022. Science: 375 (6576), 25–26.
Yasuhara, M., et al. 2020. PNAS: 117, 12891–12896.
[Response] Thank you for the suggestion. We have added two of the suggested papers but are limited by the journal formatting and do not want to generate a misbalance towards some groups of authors.

2. Phenotypic plasticity = acclimation? It can also be broad niche?
[Response] We have reworded this to "Additionally, some species maintained/increased performance in the local environment through evolutionary adaptation[9,10] or non-evolutionary acclimation.". The attribution of acclimation can be found in the first major comment of reviewer #1 (L118 in the main text).

3. "estimating plankton extinction risk11"
May like to add Yasuhara et al 2012: Yasuhara, M., eta l 2012. Ecology Letters, 15: 1174–1179.
4. "distributional shifts12,13"
May like to add Yasuhara et al 2020: Yasuhara, M., et al 2020. PNAS: 117, 12891–12896.
[Response] Included as suggested.

5. "based on the assumption that fossil assemblages have the same thermal preference as modern assemblages" You may like to use a term for this in ecology, niche conservatism.
Also this paper discussed relevant issue in details and so you may like to cite:
Yasuhara, M., et al. 2017. Biological Reviews: 92, 199–215.
[Response] As in the previous comment, we have removed our discussion about transfer function including this sentence.

6. "Each ecogroup's thermal performance is flexible and depends on the interaction between the

ecogoup's set of functional traits (size, spine, symbiont) and abiotic (temperature, nutrient, light) and biotic environmental conditions (resource competition and grazing pressure from higher trophic levels)"
"Thermal performance depends on temperature" sounds bit circular?
Also this sentence is bit complicated and not sure if "the interaction between" is for all of traits, abiotic factors and biotic factors or only one/some of them.
"biotic environmental conditions" is better to be biotic factors?
So, good to rephrase.
[Response] We rephrased it to "*Any plankton group in the model is allowed to grow in anywhere, but its emerging biogeography is constrained by the local abiotic (temperature, nutrient, irradiance) and biotic factors (prey concentration, resource competition and grazing pressure)*"

7. "These results agree with previous studies that warm-water species' optimal niche (i.e., a subset of niche where they exhibit the highest fitness) has greater variability19"
Indeed, Yasuhara et al 2012 showed higher variation in temperature-diversity relationship in the tropics than higher latitude. This is interestingly consistent and you may like to cite.
[Response] The Yasuhara et al 2012 was already cited.

8. "Unexpectedly, the modelled future foraminifera do not acclimate as much as in the deglacial warming (Fig. 2) despite experiencing a lower warming (1-4 °C compared to 6 °C)."
Why "Unexpectedly"? For me it rather makes sense. On-going warming is much more rapid and so no time for acclimation?
[Response] We removed the word "unexpectedly".

9. "Discussion" section. Better to rename? Because, you already "discussed" a lot before this section.
[Response**]** The data reanalysis and mechanism discussion now become a new section (Line 146, as suggested by reviewer#1). The rest part of discussion is modified and renamed to [Implications].

10. "In contrast to previous studies which suggest a conservative planktic foraminifera niche18,19,34, our results reveal that foraminifera can shift their thermal niche and acclimate to geological warming."
I don't disagree but I think it's better to be cautious, because previous studies discussed with species range or relative% abundance for transfer function, instead of absolute abundance (and their peak).
Thermal niche may not be very shifted in the sense of species temperature range?
I think you may like to add a bit more nuanced discussion.
[Response] We agree with the reviewer. We now detail the comparison with this study and attribute the difference to the optimal and total niche concept (see our reply to reviewer #1's comments).

11. "However, by reanalysing the species optimal temperature in Antell et al.18, we opposingly find a significant relationship (p =0.0086; Fig. 3b)"
Why? Their miscalculation?
[Response] We do not think this is a miscalculation, as it reflects the difference between optimal temperatures and the overall niche. We have clarified this point in the new section (Line153).

12. "However, such acclimation capacity gives rise to uncertainty in several fixed niche-based approaches. The modern assemblage of foraminifera has been used to reconstruct glacial surface ocean temperature36." I think you need a bit cautious discussion, because such approaches use NOT abundance peaks like this study but use more range, % abundance, etc that could be more robust and stable?
[Response] we now give more careful wording and removed our discussion about other techniques to focus on the ecological finding.

13. "This disparity might come from the fact that the increasing ocean temperature is

approaching the maximum tolerable temperature for the current foraminifera trait set in the model." It makes sense because for the late Quaternary after the MPT glacial periods are much longer than interglacials, and so they could be more adapted cold-ish environment as discussed in Yasuhara et al 2020 PNAS (and warm-adapted species were gone by post-Pliocene cooling, as seen in Yasuhara et al 2012 Ecology letters. Also see Rillo et al 2022).
[Response] We appreciate the reviewer to provide more evidence. However, we have removed this sentence based on reviewer #1's comment that our model does not constrain theoretical niche of any plankton group and cannot test this theory (although it is still a possible cause). Instead, we have provided more model analyses on the importance of food supply (see the new section and Fig. 4).

14 "in abrupt events such as the Heinrich cooling event at 16.8 ka, the onset of Holocene at 11 ka40, the modern warming27, and our future projections"
The onset of Holocene is an abrupt event?
[Response] We meant the end of Younger Dryas event and have changed the text accordingly.

Referee #3 (Remarks to the Author):

I think that there are aspects of the methods that can be clarified for greater accessibility (below), but my most substantive suggestion regards the treatment of equilibrium versus transient simulations. … In other words, is there some maximum amount of warming where acclimation cannot occur regardless of the rate of temperature change? Or conversely, would the modeled thermal preferences ultimately shift in response to equilibrium warming with the shift proportional to the total warming? From a modeling framework, it should be straightforward to test these two possibilities. If the authors would run equilibrium climate scenarios (10,000 year duration comparable to the LGM and PI states) with 1.5-2-3-4°C warming imposed (using the pre-industrial configuration) then they would have a like-for-like comparison to the LGM equilibrium experiment and can disentangle the relative role of the temperature change itself as opposed to the rate of change.
[Response] This is an important comment which we have addressed in the new section of acclimation mechanism (starting from L244). Firstly, the model does not limit the plankton niche, therefore not supporting the theoretical acclimation limits. We now clarify these in the supplementary method (Line 26): "In the modelled surface ocean, all plankton have an unlimited theoretical niche and can grow in anywhere without dispersal limits ").

To test the hypothesis that transient simulation has not reached equilibrium and consequently caused the limited niche change of foraminifera, we followed the suggestion of reviewer #3 and added a control experiment (thereby also touching on comments of reviewer #1). We added a modern spin up simulation with similar warming to the +4 °C transient experiment (global mean SST = 22.58 °C in the spin up simulation compared to 22.57 °C in the transient run) but has reached equilibrium after running for 10,000 years. This is achieved by setting a 2.5xPI CO_2 with other settings unchanged. Comparing the foram result in the equilibrium and transient model output therefore help test our hypothesis.

We plotted the foraminifera thermal niche (Fig. R3) but found no difference between equilibrium and non-equilibrium runs. This suggests that the niche change is not result of the transient/equilibrium run itself but comes from our direct LGM parameterisations which include calibrated iron cycles, wind stress pattern, orbital parameters, and ocean circulation patterns. These changes lead to a distinct net primary production-temperature relationship in the LGM (Fig.4 in the main text), and the consequently the thermal niche difference (see earlier responses to reviewer#1). The symbiont-obligate foraminifera also have a stoichiometry change which allows them to acclimate to different nutrient environment.

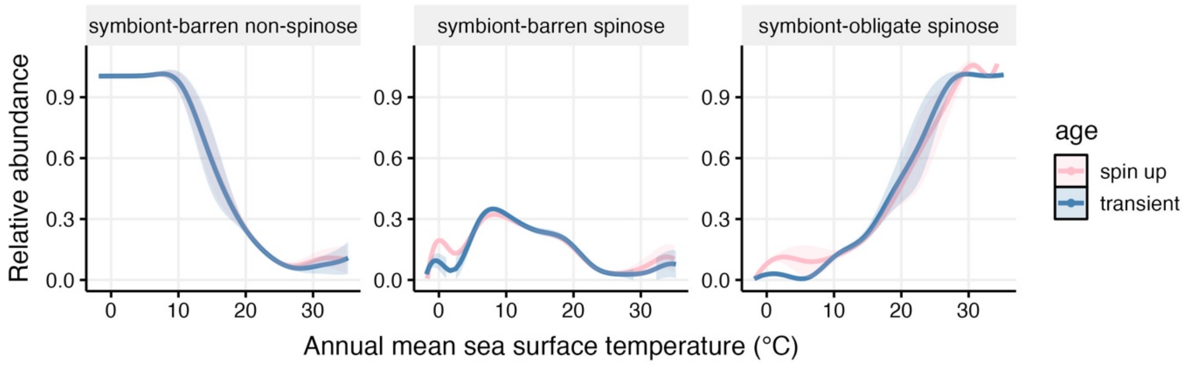

Fig. R3. The modelled foraminiferal thermal performance curve derived from spin up-based and transient-based warming experiments. We run the spin up experiment with modern configuration with 2.5 × pre-industrial CO_2 concertation (i.e., 695 ppm) for 10000 years to reach the similar warming level to the transient under equilibrium state. The figure shows the final year of spin up model output and the transient model is the 2100 under 4 degrees warming as in the main manuscript. However, the result suggests no difference between equilibrium and non-equilibrium state, which contrasts with our hypothesis.

The description of the ForamEcoGENIE model in the Methods section could be improved for clarify. … What are the controls on modeled NPP? Is there a way to separate the relative impact of temperature and NPP change on the decline in foraminiferal biomass? Finally, the Methods describes improvements made to ForamEcoGENIE from a previous publication, but it is not clear how these improvements were calibrated against observations (what observations?) or what impact these improvements have to the particular experiments detailed here.
[Response] We have improved our method description based on the reviewer's suggestion. We also added a more technical description in the supplementary material.

+ We now only use cGENIE in the paper to avoid unnecessary confusion
+ We corrected the grid resolution to "36 × 36 equal area grid (10° longitude and uniform in the sine of latitude)".
+ We added a new section of plankton biomass dynamic description in the supplementary information (Line 96). These should describe how plankton influence biogeochemical tracers.
+ We added how we calculate NPP in the main text (L278) and Supplementary Method 1.4.5 (L191)
+ Unfortunately, we cannot split the effects of temperature and NPP yet because these two drivers are closely coupled in the ocean. For example, temperature influences the photosynthetic rate, and ocean stratification which in turn influence the nutrient distribution and NPP.
+ We now clarify (a) our changes (b) the reasons and (c) the consequent impact in the Supplementary Method (Line 55-95, Supplementary table 2; Supplementary Figure 6). Briefly, our published configuration produces unrealistic relative abundance of some groups and fails to achieve viable population in the LGM. Our modifications are to solve these.

The figures are very nice, but I think it would help to add vertical lines in Figure 2b to show the lack of a shift in the thermal preference in the future warming scenarios (as in Fig 1).
[Response] We have made figures consistent and improved visualisation.

Reviewer Reports on the First Revision:

Referees' comments:

Referee #1 (Remarks to the Author):

The authors tackle in their manuscript a problem of considerable scientific interest and societal relevance: the question of whether or not species and communities that populate the Earth today will be able to respond to projected future warming. I note that the manuscript has been reviewed before and in view of the initial comments, the response by the authors and my own observations, I can offer the following comments:

Species can respond to changes in their habitat either by the presence of broader thermal tolerance than in the realised niche (acclimation) or by the evolutionary emergence of new traits (adaptation). The referees rightly pointed out that the concepts as used in the paper were not entirely consistent. The revised version attempts to rectify this, but in my view the point of the distinction is missed when interpreting the results of the study. The authors provide no evidence that could decisively discern whether the response of fossil foraminifera communities to warming in the geological past was due to acclimation or adaptation. At the same time, their model only considers acclimation, not adaptation. So how can one be sure that the case from the geological past demonstrated model skill sufficient to predict future change?

The way that the authors leverage data from the fossil record to define what they call optimum niche is problematic. It appears that in the first version of the manuscript, they started by using absolute abundance (shell flux). This is indeed the correct way to detect the position of optimum performance of a species, but data of this type are rare in geological records so the authors now reverted to relative abundances. These show how a species performs relative to other taxa of the studied group, providing information on relative optimum. Inverting the relative abundance data into information on absolute (and hence biologically relevant) optima is hard because of ambiguity: the same relative abundance pattern can emerge out of completely different and even completely opposing patterns of absolute abundance. Consider the case of two species with an asymmetric temperature response curve, one with warm water optimum but long tail towards cold temperatures, the other the opposite. When converted to relative abundances, each species would reach higher relative abundance in the opposite end of its actual optimum, simply because of its higher tolerance at that temperature end. I cannot see how the authors have dealt with this in their model and in particular, when considering fossil data and relative abundances, I cannot see how can one discern if a shift in the position of the relative-abundance based optimum is not due to shifts of other species without any change in the given species or by a change in another aspect of the niche, like tolerance, rather than the optimum.

Considering the above, one must ask if the data that the authors present provide unambiguous evidence for optimum niche shift or not. The arguments that the authors use on Line 126 and onwards only deal with alternative explanations for the origin of the postulated shift in optimum niches. But what is none

occurred? The authors try to address this by reanalysing the presence-absence data from ref 12 to show that whilst absolute temperature range remained unchanged through time (finding of ref 12) the shape of the temperature response within this range (optimum niche of the authors) did. First, I cannot grasp how the authors could have reconstructed the shape of the thermal response within a species thermal range from presence-absence data? Such data simply cannot inform on where a species is most abundant and the chosen approach of defining optimum by "highest species occurrence probability" is difficult to understand. If a species occupies a given thermal range, then the probability of it occurring within this thermal range is 100% throughout the thermal range? Second, the authors refer in their rebuttal to arguments in ref 22, which also claims that the niche remained conserved, but they provide no discussion on the main argument that ref 22 made: if any aspect of the niche would change, one should encounter in the geological past fossil assemblages which have no compositional analogs in the modern ocean. Finally, I find it difficult to understand what exactly is shown in Figure 2. The individual points appear to represent pairs of samples in the 700 kyr time series, each pair divided by 8 kyr, corresponding to differences in local temperature between +10 to -10°C, probably compared to overall mean. If this is correct then the interesting pattern in the diagram is not the significant correlation (explaining 3 % of the variance), but the existence of very many assemblages with small (close to 0) change in species thermal optimum (which I presume is the sum of the changes over the 24 species – but then how are negative values compensating for positive values per species treated?) but large change in habitat temperature (I find it difficult to understand why this always remain within about 2°C). In other words: there are many situations in the past where warming or cooling perturbed the habitat temperature, but no acclimation occurred. How come?

I concur with the authors that the implications of their finding would be profound and highly relevant. They would provide the first empirical evidence that plankton communities (in fact any communities) may not be able to cope with future warming by plasticity (acclimation). However, in view of the above comments, I am not convinced that the authors provided sufficiently decisive evidence in support of such a conclusion. If the fossil record does not show any evidence for shifts in optimum niche, but the model does, then the model is wrong in its assumptions. Even if one accepts that the fossil record shows shifts in optimum niche, there is no way to show that this was due to acclimation and not due to evolution. The latter would render the model results irrelevant, because the model fit to the fossil data was for the wrong reasons.

Referee #2 (Remarks to the Author):

I found the revision was nicely done. I appreciate your great effort. Figs are really neatly/carefully done, I like them very much. Congratulations!!

Referee #3 (Remarks to the Author):

This is my second review of Ying et al's study 'Marine zooplankton acclimated to warming in the geological record face limits by the next century.' My initial review was favorable, and the authors have done a considerable amount to address comments from the initial reviews.

Previously, my main comment centered around the difference between equilibrium simulations to compare the modeled LGM and pre-industrial states versus transient simulations of future warming. The author's rebuttal importantly described the addition of a new equilibrium warming experiment at 4°C warming relative to the PI and they state that they 'found no difference in the equilibrium and nonequilibrium runs' in the foraminiferal thermal niche. They refer to these results as Figure R3, but I can't find this figure (neither Supp. Fig 3 and Extended Data Fig. 3 show these results, so I'm not sure what R3 refers to)? Also, this question of
the equilibrium versus nonequilibrium result, and hence the importance of timescale, remains unaddressed in the main text and their rebuttal actually seems counter to the statement on Line 287 of the manuscript that "this highlights the importance of climate change timescale in plankton acclimation, which involves the environmental selection of successful phenotypes in offspring population and are susceptible to the high stress of abrupt change" after describing the result (not shown) that foraminifera mostly migrated in response to abrupt events like Heinrich cooling at 16.8 ka and the YD (alongside historical warming). It seems worth further considering and discussing the comparison between the transient and equilibrium warming scenarios – because the ocean circulation response and impact on nutrients must have been different? In the transient scenario, warming will have led to stratification by the end of the century, which will impact nutrient availability and consequently explain some of the NPP change. The equilibrium run will not show the same stratification response after 10,000 years at 2.5xCO_2. So, if these two results are similar in terms of the modeled thermal niche for foraminifera, then this implies that ocean structure and nutrient availability are not driving the inability of foraminifera to acclimate. I found this discussion absent from the new section (particularly from paragraph starting at Line 277), but I think this analysis would strengthen the manuscript by providing a clearer mechanistic explanation for the future lack of acclimation.

I include comments by line below, but I should note that I found many more instances where the text requires grammatical edits – especially the Methods and Supplement. I have not provided comprehensive line-by-line edits, so in addition to my suggestions below, I recommend more careful editing prior to resubmission.

Line 24: the results 'show' that foraminifera
Line 29: the model 'suggests' that the

Line 41: This sentence is vague - in response to what environmental changes? what marine taxa?

Line 43: are often observed 'in' marine microbes

Line 50: contribute roughly half 'of' modern pelagic

Line 55: the addition of definitions is useful for fundamental niche and realized niche, but it would be ideal to clarify optimal niche on Line 57 as well

Line 63: I would rephrase ‘long extensive geological record,’ given that the study is evaluating the recent past (LGM to present), which is short even compared to the reference above (Line 53) for the past 700 ka.

Line 78: define ‘thermal performance curves’ here as relative abundance as a function of temperature.

Line 78-79: is this definition of optimal niche consistent with the reference given on Line 57 or is your definition different? Also, please re-word so that it is clearer whether the definition of optimal niche is being applied to fossil data, model output, or both?

Line 80: wording is again unclear as to whether you are referring to reconstructed thermal performance curves from model, data, or both.

Line 94: the wording of the portion of this sentence that begins on Line 96 is unclear - do you mean ecological setting in the model? Is this saying that there are different genotypes of G. bulloides found in open ocean versus upwelling regimes that have different responses but that these responses are not captured by the model?

Line 99: for all groups, please give consistent descriptions of the change in optimal temperature between the LGM and PI - either as absolute values or changes or both.

Line 121: delete ‘as acclimation’

Line 124: can you clarify the distinction between thermal acclimation and adaptation (the latter meaning evolutionary adaptation)?

Line 127: global gene ‘flow’ of foraminifera

Fig. 1 - I like the addition of the foram cartoons to show spinose and non-spinose - can you add to the cartoon to indicate symbionts as well (perhaps small green dots)? The legend refers to (a) and (b) as cGENIE output and fossil data, respectively, but the figure has panels a-f. Edit this to ‘top row’ and ‘bottom row.’ I also suggest adding a header over each row indicating ‘model’ or ‘data’ rather than leaving this on the y-axis only.

Line 154: that ‘characterizes’

Line 188: explain the warming scenarios a bit more in the main text - I suggest using some of the text in the Fig. 3 legend. This is relevant for the next sentence, which suggests the scenarios are partly data-constrained in terms of the rate of warming, since the present-day temperature change is reproduced.

But why is there a gap in the figures between modern (2020) and the first results from the idealized warming scenarios? Also, on Fig. 3 - it's confusing for the warming scenarios to be identified based on the amount of warming by 2100 relative to the 1900-1950 average, but then the results plotted in Fig. 3 (and described in the text) to show temperature change relative to 1975. Perhaps change the y-axis to show temperature change relative to the 1900-1950 average? (This is what is done in Supp. Fig. 2).

Line 191: this is awkward because the temperature change between 1975 and 2100 are different than the values given to label the scenarios.

Line 213: Add something here about the modeled foraminiferal biomass change?

Line 221-223: Why not also mention the modeled increase in the Southern Ocean as well? (Fig. 3c - right)

Line 241: 'is' evident today in coral

Line 277: I'm surprised this paragraph doesn't discuss the difference in ocean circulation (ocean structure) responses to slow versus rapid warming and how this should impact nutrient availability and NPP. The future experiments must be showing increased vertical stratification and it would be useful to contrast the vertical stratification in the transient future scenarios against the difference in ocean density structure between the LGM and PI simulations run at steady state.

Line 287: the reference to Fig. 3 is misleading given the reference to Heinrich cooling and the YD - these are not shown anywhere.

Line 300: The 'Implications' section needs grammatical editing

Line 352: carbon 'cycling,' particularly

Line 357: functional 'traits'

Line 359: 'and biotic interaction'

Line 371: strange wording - the 'simulated' OGM ocean

Line 447: calibrated 'using' a Bayesian

Line 449: reconstruction 'from' the MARGO 'dataset'

Line 455: edit for grammar and clarity; a suggestion is 'These limitations do not affect our inference of acclimation because a shallow thermocline restricts most species to the shallow mixed layer in the high latitudes and symbiont bearing species in the low latitudes live in the mixed layer to obtain sufficient

solar irradiance’

Line 475: The ‘resulting’ standard deviation…could provide ‘an assessment’ of sampling effort

Overall: all of the Methods text needs grammatical editing

Referee #3 (Remarks on code availability):

While the cGENIE source code is available at https://github.com/derpycode/cgenie.muffin, I cannot find the directory genie-userconfig/FORAMECOGEM/readme.md with specific experiments files mentioned in the Code Availability section.

Author Rebuttals to First Revision:

Responses to reviewers

We want to thank our reviewers who provided constructive comments in this round of review. Below we are addressing the key comments with our one-by-one response. The reviewers' comments are coloured in black, and our responses are in blue.

Referee #1

Species can respond to changes in their habitat either by exploting a broader thermal tolerance than in the realised niche (acclimation) or by the evolutionary emergence of new traits (adaptation). The referees rightly pointed out that the concepts as used in the paper were not entirely consistent. The revised version attempts to rectify this, but in my view the point of the distinction is missed when interpreting the results of the study. The authors provide no evidence that could decisively discern whether the response of fossil foraminifera communities to warming in the geological past was due to acclimation or adaptation. At the same time, their model only considers acclimation, not adaptation. So how can one be sure that the case from the geological past demonstrated model skill sufficient to predict future change?
[Response] Previous studies have provided evidence that planktonic foraminifera could only experience acclimation and no adaptation since the LGM. The most recent origination amongst modern morphologically defined foraminifera species occurred diachronously between 2.2-32.2 Ma (Sexton & Norris, 2008; Kucera & Schonfeld, 2009), hence much earlier than the LGM (19-21 ka). The evolutionary history of the foraminifera morphospecies has been very well understood (Aze et al 2011).

Besides, while there is evidence for genetic changes within the foraminiferal morphotypes, termed cryptic species, they point to the most recent genetic split during MIS7 roughly 200 kyrs ago (Quillévéré et al. 2013), and therefore older than the LGM. Moreover, the foraminifera ecogroup presenting acclimation in our study has the least cryptic species in the community (Morard et al. 2024). Some dominant species (e.g., *T. sacculifer*) in this ecogroup is even characterised by no cryptic species. This reinforces that no genetic divergence occurred to support their evolutionary change after decades of studying LGM foraminifera samples. All these information is clarified for reader in the L113-115.

The way that the authors leverage data from the fossil record to define what they call optimum niche is problematic ... These show how a species performs relative to other taxa of the studied group, providing information on relative optimum. Inverting the relative abundance data into information on absolute (and hence biologically relevant) optima is hard because of ambiguity: the same relative abundance pattern can emerge out of completely different and even completely opposing patterns of absolute abundance. Consider the case of two species with an asymmetric temperature response curve, one with warm water optimum but long tail towards cold temperatures, the other the opposite. When converted to relative abundances, each species would reach higher relative abundance in the opposite end of its actual optimum, simply because of its higher tolerance at that temperature end. I cannot see how the authors have dealt with this in their model and in particular, when considering fossil data and relative abundances, I cannot see how can one discern if a shift in the position of the relative-abundance based optimum is not due to shifts of other species without any change in the given species or by a change in another aspect of the niche, like tolerance, rather than the optimum.

[Response] We fully agree with the reviewer that absolute abundance (i.e., foraminifera accumulation rate) would provide the best data, and the relative abundance is influenced by the abundance of other species. We now clearly state such limitations in the Lines 85-90.

However, the relative abundance is still a useful metric to quantify fitness as few foraminifer taxa often strongly dominate assemblages when they are under optimal conditions and these are the ones our arguments are based on such as *T. sacculifer, Gs. ruber, and N. pachyderma*. Furthermore,

we have added optimal temperature data based on maximal body size (Schmidt et al., 2004) in the Extended data Table 1, and now expand the table to include the culture measurement based on highest growth rate (Lombard et al., 2009). All these data show similar results and hence corroborate the ability to use relative abundance in a taxon which is not very diverse (50 modern morphotypes). Thus, we provide evidence that the relative optimum is representative enough to quantify species optimal condition in a paleo-study. (Line 87-90).

In response to the reviewer's example, the relative thermal performance could differ from the absolute thermal performance but cannot have opposing pattern (Fig. R1). If a species has greater aboslute abundance than another species (A > B), it always achieves greater relative abundance (A/total > B/total). This method therefore can infer the optimal condition in our understanding.

Furthermore, the relative abundance change is influenced but not caused by species replacement (Line 129-131). We have provided our modelled (Supp. Fig1) and observational evidence to make this point, as already presented in the initial manuscript. We use core sample data (MD01-2390 a tropical core in the western Pacific warm pool) here to demonstrate the trend of absolute abundance based on species accumulation rate (Steinke et al. 2008) in a region representing the warmest ocean (>28 degrees C in the modern ocean). In Fig. R2, we show that all dominant symbiont-obligate foraminifera species including *T. sacculifer, G. ruber, and G. siphonifera* in this core increased abundance by 3-6-fold. Therefore, the absolute abundance/optimum has the same trend as relative abundance. This core has also a non-analogue assemblage in the LGM (Steinke et al. 2008), which we will expand on in the below comment regarding the assemblage method.

Finally, the tolerance range and optimum range are two independent biological traits. The change of tolerance range does not have to influence the optimal condition.

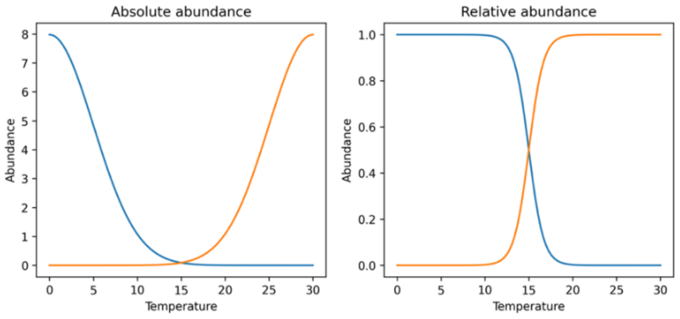

Fig. R1 The asymmetric thermal response curve of two species (blue and yellow) and its corresponding relative abundance distribution as described by the reviewer. Despite lower accuracy of relative abundance, it still shows the thermal preference of two species in cold and warm environments.

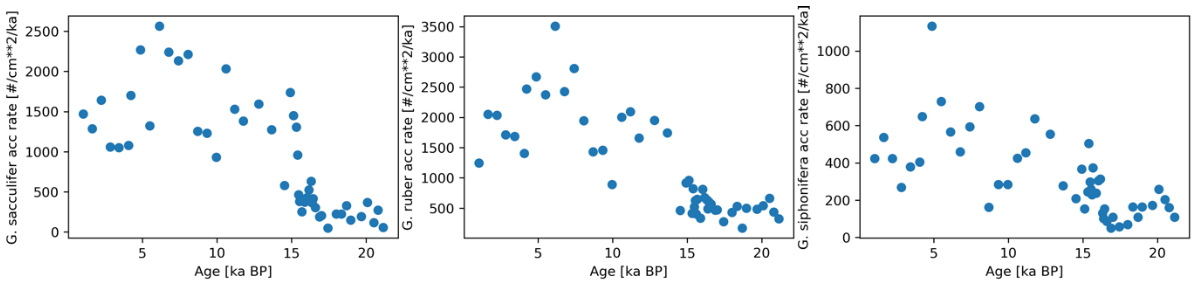

Fig. R2 The foraminifera accumulation rate in the MD01-2390 (South China Sea). The symbiont-obligate foraminifera abundance increased by 3 to 6 times in the last deglacial warming, in agreement with our model's estimate of a 3-fold increase.

Considering the above, one must ask if the data that the authors present provide unambiguous evidence for optimum niche shift or not. The arguments that the authors use on Line 126 and onwards only deal with alternative explanations for the origin of the postulated shift in optimum niches. But what if none occurred?

[Response]We did not claim acclimation by ruling out other alternatives as the reviewer implies. Instead, we interpret acclimation using thermal performance curves, which have been very widely used in thermal ecology studies (e.g., Padfield et al. 2016; Schaum et al. 2017). We have improved our introduction of the method (Lines 81-85) and given the related references to avoid such misunderstanding.

The authors try to address this by reanalysing the presence-absence data from ref 12 to show that whilst absolute temperature range remained unchanged through time (finding of ref 12) the shape of the temperature response within this range (optimum niche of the authors) did. First, I cannot grasp how the authors could have reconstructed the shape of the thermal response within a species thermal range from presence-absence data? Such data simply cannot inform on where a species is most abundant and the chosen approach of defining optimum by “highest species occurrence probability” is difficult to understand. If a species occupies a given thermal range, then the probability of it occurring within this thermal range is 100% throughout the thermal range?

[Response] We would like to emphasise that this section was expanded to address previous reviewer's concern and show the robustness of our result. Therefore, it is not directly linked to our LGM/PI results despite giving more evidence of acclimation. In contrast, we fully agree with the reviewer the presence-based method has its own limitation, and this is part of our motivation to build the abundance-based method and use a more robust subset of their dataset. We are happy to move this section into the supplementary information if necessary.

Regarding the reviewer's comment, the species fitness in Antell et al. (2021) is estimated by probability density (not probability as corrected by the reviewer) rather than abundance. We cautioned the limitation of this method in L160 and L169. However, the presence-based method still generates same conclusion as our abundance-based method, showing its ability to capture the optimal niche change (L162).

I find it difficult to understand what exactly is shown in Figure 2. The individual points appear to represent pairs of samples in the 700 kyr time series, each pair divided by 8 kyr, corresponding to differences in local temperature between +10 to -10°C, probably compared to overall mean. If this is correct then the interesting pattern in the diagram is not the significant correlation (explaining 3 % of the variance), but the existence of very many assemblages with small (close to 0) change in species thermal optimum (which I presume is the sum of the changes over the 24 species – but then how are negative values compensating for positive values per species treated?) but large change in habitat temperature (I find it difficult to understand why this always remain within about 2°C). In other words: there are many situations in the past where warming or cooling perturbed the habitat temperature, but no acclimation occurred. How come?

[Response] We put this comment earlier so that it follows the previous one. In Fig.2, each point represents one species not one core sample. Therefore, it does not show the temperature change in each core neither the assemblage averaged thermal optimum. Instead, it describes the temporal evolution of each species' optimal temperature between two closest time bins. The y axis is the species optimal temperature difference, and the x axis is the related abiotic temperature change.

As a result, Fig. 2 shows (1) there were optimal niche change for species; and (2) as the reviewer suggests, adaptive response occurred in the past where warming or cooling perturbed the habitat temperature. Both messages are in line with our LGM/PI data.

the authors refer in their rebuttal to arguments in ref 22, which also claims that the niche remained conserved, but they provide no discussion on the main argument that ref 22 made: if any aspect of the niche would change, one should encounter in the geological past fossil assemblages which have no compositional analogs in the modern ocean.

[Response] As we answered in the last rebuttal letter, acclimation does not necessarily lead to a non-analogue assemblage. It is fully possible for the several dominating species to acclimate the warming

and keep the species composition similar. Moreover, the detection of analogue assemblage has its own limitations. For example, Strack et al. (2022) found the non-analogue assemblage based on Morisita-Horn distance and 99th percentile, while Jonkers et al. (2023) found no non-analogue assemblage (use square chord distance and 95th percentile). Steinke et al. 2008 found non-analogue assemblages in the LGM. We reckon the methodological challenges in determining non analogues are beyond this study's scope. This paragraph has been rewritten to discuss the trend of relative abundance and absolute abundance. Therefore, the ref22 is not included now.

Referee #2:

I found the revision was nicely done. I appreciate your great effort. Figs are really neatly/carefully done, I like them very much. Congratulations!!

[Response] Thank you for reviewing and improving our manuscript!

Referee #3

This is my second review of Ying et al's study 'Marine zooplankton acclimated to warming in the geological record face limits by the next century.' My initial review was favourable, and the authors have done a considerable amount to address comments from the initial reviews. ... The author's rebuttal importantly described the addition of a new equilibrium warming experiment at 4°C warming relative to the PI and they state that they 'found no difference in the equilibrium and nonequilibrium runs' in the foraminiferal thermal niche. They refer to these results as Figure R3, but I can't find this figure (neither Supp. Fig 3 and Extended Data Fig. 3 show these results, so I'm not sure what R3 refers to)?

[Response] Thank you for reviewing our manuscript again! The missing figure is put below.

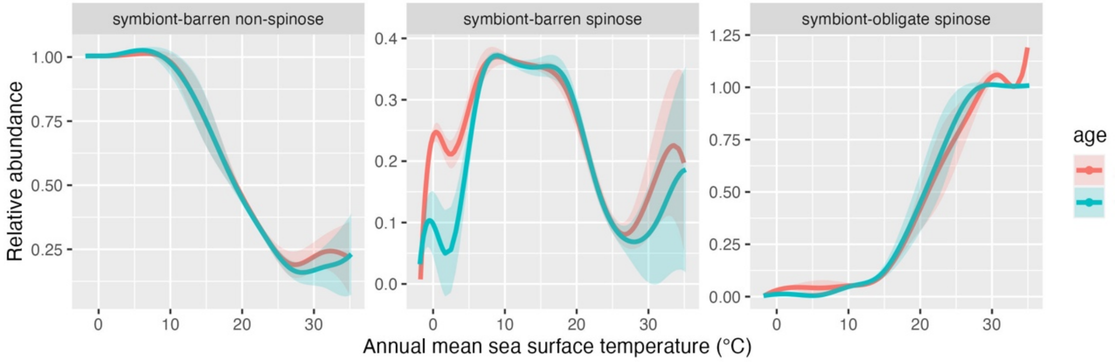

Fig. R3. The foraminifera thermal performance curves in the transient warming and the spin up warming.

Also, this question of the equilibrium versus nonequilibrium result, and hence the importance of timescale, remains unaddressed in the main text ... In the transient scenario, warming will have led to stratification by the end of the century, which will impact nutrient availability and consequently explain some of the NPP change. The equilibrium run will not show the same stratification response after 10,000 years at 2.5xCO2. So, if these two results are similar in terms of the modeled thermal niche for foraminifera, then this implies that ocean structure and nutrient availability are not driving the inability of foraminifera to acclimate. I found this discussion absent from the new section (particularly from paragraph starting at Line 277), but I think this analysis would strengthen the manuscript by providing a clearer mechanistic explanation for the future lack of acclimation.

[Response] In the last rebuttal, we added model runs where we controlled the surface warming level and compared a steady-state run against the transient run. The resulting foraminiferal thermal niche changed little (copied above as Fig. R3). The impact of time scale on nutrients shows mostly in the

high latitudes, where the niche conservative groups? dominate (Supp. Fig. 10). Therefore, we decided to focus our analysis on the biomass change and not on the thermal niche.

We performed additional transient runs to demonstrate further the impact of timescales. We forced the model with different emissions and warming rates for the model to reach a 4-degree warming (relative to the 1900-1950 average) by 2100, 2540, 2990, 3460. In combination with our steady state experiment, these analyses reveal how ocean circulation changes at different rates of warming (see Supplementary Information). Warming increases stratification and reduces nutrient supply and foraminifera biomass. However, after a sufficiently long time, ocean circulation mitigates such change (Fig. R4). This difference is mostly evident in the Southern Ocean, whereas the North Atlantic continues to be nutrient depleted because of a less effective AMOC (Supp. Fig 10). These changes are included in the supplementary information, and we have rewritten the paragraph accordingly (L303).

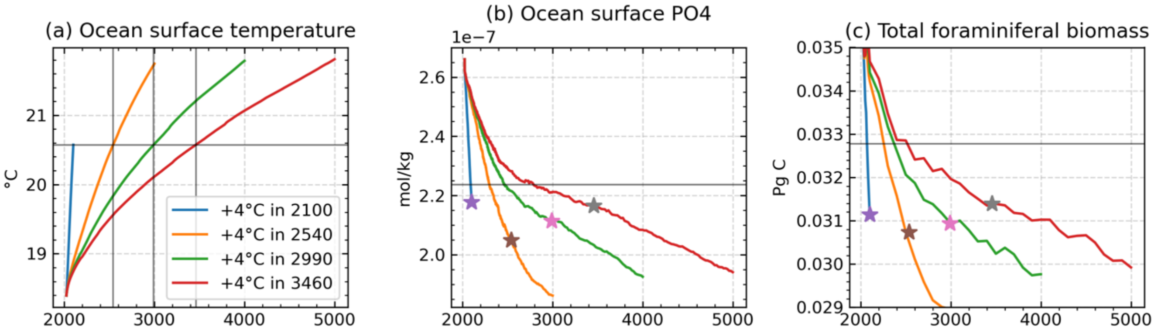

Fig. R4. Model responses of surface mean nutrient (shown here as PO4) and total foraminifera biomass to a 4-degree warming under different warming rates. The stars in (b-c) represent states when the ocean surface reaches a 4 °C warming relative to the 1900-1950 average. The horizontal lines in subplots (b,c) show the state in surface nutrient and foraminifera biomass under fully equilibrium state. Decreasing the warming rate damps any resulting ocean stratification and associated reduction of surface nutrient supply. The spatial and vertical distribution of nutrient difference can be found in Supplementary Figures 9-10.

…text requires grammatical edits – especially the Methods and Supplement.

[Response] Thank you for helping check this, we did a careful grammar check in the whole manuscript in this version and implemented all minor spelling and grammar suggestions by the reviewer in the following.

Line 41: This sentence is vague - in response to what environmental changes? what marine taxa?
[Response] We now change it to "*In response to warming, some marine plankton and animal species shifted their habitat to track suitable temperatures*".

Line 55: the addition of definitions is useful for fundamental niche and realized niche, but it would be ideal to clarify optimal niche on Line 57 as well
[Response] The definition of optimal niche is added in the Line 57. We remove the fundamental niche because it links to evolution and not so related to this study.

Line 63: I would rephrase 'long extensive geological record,' given that the study is evaluating the recent past (LGM to present), which is short even compared to the reference above (Line 53) for the past 700 ka.

[Response] We remove the word "long" and change it to "extensive foraminiferal microfossil record".

Line 78: define 'thermal performance curves' here as relative abundance as a function of temperature.
[Response] This definition is added.

Line 78-79: is this definition of optimal niche consistent with the reference given on Line 57 or is your definition different? Also, please re-word so that it is clearer whether the definition of optimal niche is being applied to fossil data, model output, or both?

[Response] It is consistent with the earlier mentioned text (high fitness).
We rephrase this to "*The optimal niches are quantified based on the TPCs as the temperatures leading to abundances exceeding half of the highest abundance.*

Line 80: wording is again unclear as to whether you are referring to reconstructed thermal performance curves from model, data, or both.

[Response] This sentence is removed in the revised paper because it is too wordy and unnecessary here.

Line 94: the wording of the portion of this sentence that begins on Line 96 is unclear - do you mean ecological setting in the model? Is this saying that there are different genotypes of G. bulloides found in open ocean versus upwelling regimes that have different responses but that these responses are not captured by the model?

[Response] Indeed, we change it to "*one ecological setting in the model*".

Line 99: for all groups, please give consistent descriptions of the change in optimal temperature between the LGM and PI - either as absolute values or changes or both.
[Response] All groups are reported as absolute values now.

Line 124: can you clarify the distinction between thermal acclimation and adaptation (the latter meaning evolutionary adaptation)?
[Response] This wording relates to our changes in response to the reviewer#1's comment. We clarify the difference in the L113-116

Line 127: global gene 'flow' of foraminifera
[Response] This paragraph is rewritten for replying our reivewer#1.

Fig. 1 - I like the addition of the foram cartoons to show spinose and non-spinose - can you add to the cartoon to indicate symbionts as well (perhaps small green dots)? The legend refers to (a) and (b) as cGENIE output and fossil data, respectively, but the figure has panels a-f. Edit this to 'top row' and 'bottom row.' I also suggest adding a header over each row indicating 'model' or 'data' rather than leaving this on the y-axis only.

[Response] Thank you for your appreciation! These suggestions are implemented in the Fig. 1

Line 188: explain the warming scenarios a bit more in the main text - I suggest using some of the text in the Fig. 3 legend. This is relevant for the next sentence, which suggests the scenarios are partly data-constrained in terms of the rate of warming, since the present-day temperature change is reproduced.

[Response] We change these sentences to "*We used observational CO2 to force the model from pre-industrial to the present day, and four idealised linear CO2 forcings to simulate future warming scenarios (+1.5, 2, 3, and 4°C by 2100 relative to the 1900-1950 average; Fig. 3a). By reproducing the observations that the global mean sea-surface temperature in the present-day (2022) is ~0.6 °C warmer than the 1900-1950 average (Fig 3a), the model demonstrates its ability to simulate future scenarios in terms of the rate of warming.*"

But why is there a gap in the figures between modern (2020) and the first results from the idealized warming scenarios?

[Response] This is now fixed.

Also, on Fig. 3 - it's confusing for the warming scenarios to be identified based on the amount of warming by 2100 relative to the 1900-1950 average, but then the results plotted in Fig. 3 (and described in the text) to show temperature change relative to 1975. Perhaps change the y-axis to show temperature change relative to the 1900-1950 average? (This is what is done in Supp. Fig. 2). Line 191: this is awkward because the temperature change between 1975 and 2100 are different than the values given to label the scenarios.

[Response] The figures (Fig. 3a, Extended Data Fig 7) and related description are changed to the comparison with the 1900-1950 average for consistency. Also, the Fig. 3b reference year is changed to present day for consistency with the Fig. 3c and the text.

Line 213: Add something here about the modeled foraminiferal biomass change?

[Response] We add "we conclude that *foraminifera will not track the pace of temperature change and their global distribution and biomass will be reduced in the future.*" here.

Line 221-223: Why not also mention the modeled increase in the Southern Ocean as well? (Fig. 3c - right)
[Response] This has been mentioned as 'subantarctic zone', we changed it to "Southern Ocean".

Line 277: I'm surprised this paragraph doesn't discuss the difference in ocean circulation (ocean structure) responses to slow versus rapid warming and how this should impact nutrient availability and NPP. The future experiments must be showing increased vertical stratification, and it would be useful to contrast the vertical stratification in the transient future scenarios against the difference in ocean density structure between the LGM and PI simulations run at steady state.

[Response] This has been addressed in the major comment.
Line 287: the reference to Fig. 3 is misleading given the reference to Heinrich cooling and the YD - these are not shown anywhere.

[Response] This sentence is removed to avoid potential confusion regarding the time we modelled.

While the cGENIE source code is available at https://github.com/derpycode/cgenie.muffin, I cannot find the directory genie-userconfig/FORAMECOGEM/readme.md with specific experiments files mentioned in the Code Availability section.

[Response] We provide the correct link and instruction now.

References

Aze, T. et al. A phylogeny of Cenozoic macroperforate planktonic foraminifera from fossil data. Biological Reviews 86, 900–927 (2011).

Sexton, P. F. & Norris, R. D. Dispersal and biogeography of marine plankton: Long-distance dispersal of the foraminifer Truncorotalia truncatulinoides. Geology 36, 899–902 (2008).

Siccha, M., Trommer, G., Schulz, H., Hemleben, C. & Kucera, M. Factors controlling the distribution of planktonic foraminifera in the Red Sea and implications for the development of transfer functions. Marine Micropaleontology 72, 146–156 (2009).

Morard, R. et al. The global genetic diversity of planktonic foraminifera reveals the structure of cryptic speciation in plankton. Biol. Rev. n/a, (2024).

Lombard, F., Labeyrie, L., Michel, E., Spero, H. J. & Lea, D. W. Modelling the temperature dependent growth rates of planktic foraminifera. Marine Micropaleontology 70, 1–7 (2009).

Schmidt, D. N., Renaud, S., Bollmann, J., Schiebel, R. & Thierstein, H. R. Size distribution of Holocene planktic foraminifer assemblages: biogeography, ecology and adaptation. Marine Micropaleontology 50, 319–338 (2004).

Steinke, S., Yu, P.-S., Kucera, M. & Chen, M.-T. No-analog planktonic foraminiferal faunas in the glacial southern south china sea: implications for the magnitude of glacial cooling in the western pacific warm pool. Mar. Micropaleontol. 66, 71–90 (2008).

Strack, A., Jonkers, L., C. Rillo, M., Hillebrand, H. & Kucera, M. Plankton response to global warming is characterized by non-uniform shifts in assemblage composition since the last ice age. Nat Ecol Evol 6, 1871–1880 (2022).

Jonkers, L. et al. Strong temperature gradients in the ice age North Atlantic Ocean revealed by plankton biogeography. Nat. Geosci. 16, 1114–1119 (2023).

Reviewer Reports on the Second Revision:

Referees' comments:

Referee #1 (Remarks to the Author):

I am pleased to comment on the second revised version of the manuscript, noting that the authors have gone some way towards resolving most of the issues I raised. As I noted in my previous review, there is no doubt that the authors tackle in their manuscript a problem of considerable scientific interest and societal relevance. Indeed, knowing the limits to acclimation of species is essential to predict whether or not species and communities that populate the Earth today will be able to respond to projected future warming without loss in their overall ecosystem function.
The authors address this issue by designing an ecosystem model that allows for acclimation (i.e. the shape of the performance against temperature curve of a species is not prescribed but emerges from the model). They compare the model with data for the last deglaciation and conclude that the model performance is consistent with data. This is important because the model can then be used, and this is the fundamental advance of the paper, to explore future scenarios and because of the nature of the model also to query the underlying mechanisms of the projected ecosystem response.
As every study dealing with the fossil record, a large part of the comparison between model and fossil data relies on assumptions, such as that adaptation (evolution) did not play an important role in the studied plankton at least since the last glacial maximum and that the climate model used generates an accurate temperature hindcast for the last ice age. On balance, the authors provided good examples of arguments of why it the assumptions then made are plausible and I thus believe that when these assumptions are made explicitly known to the reader and treated as in their response, the study as a whole may be presented to the public. With the assumptions mentioned, I tend to agree with the implications of the study as stated in the last paragraph, and because these are of considerable impact, I am now more inclined towards recommending the manuscript for publication.

Prior to that, I would like the authors to consider the following comments:

1) The response to my first two comments (lack of adaptation and relative vs absolute abundance) needs to be incorporated into the paper. The former is treated well especially in the second paragraph of the response where the authors point towards largest changes indicated for species that contain no cryptic diversity. It is still circumstantial, but for fossil data we will likely never have more conclusive evidence. For the latter, the inclusion of the additional analyses is a good idea (especially Figure R1), but the argument presented with Figure R1 is wrong. I have included below an example that illustrates this.

(figure in attached PDF)

Red and blue are hypothetical absolute abundance patterns of two species, grey is the relative abundance of the blue species. This reverses because the thermal niche of the red species is narrower.

2) I would indeed urge the authors to move the presence-absence data analysis into the supplement. There are too many problems associated with it. First of all, in their response to my first previous comment, the authors provide good evidence for a lack of adaptation since the LGM, but at the same time their concede in their response themselves that adaptation may have occurred prior to that certainly since 700 kyr. Including this analysis in the paper would require a different section reasoning why adaptation did not play a role on the longer time scale. Second, I still have difficulties with Figure 2. Its current caption remains completely impenetrable and even after reading the response by the authors, I do not understand what each of the points in the graph means. If each dot represent one species, then why is there more then 30 dots in the graph? The authors only write “each species”, without explaining how many they are and how many times the same species may appear. Does this include rare species and how is their niche calculated? If the graph shows for all species their shift in optimum temperature for each pair of samples at 8 kyr distance, then we should have in the graph 700 kyr / 8 kyr * 30 species = 2650 data points. I can see an order of magnitude less. Further, it remains a mystery, why so many species appear to have changed their thermal optimum by such large numbers (in excess of 10°C) over just 8 kyr? The authors must surely be aware of the fact that their own analysis of the last deglaciation (about 20 kyr) does not indicate a single change in optimum temperature of that magnitude. My only explanation for this pattern is the noisy nature of the 700 kyr data, especially against rare species combined with the indirect way of determining the optimum temperature for presence and absence. This is because the latter requires high and equal spatial sampling throughout the studied interval. If this is not given, the optimum temperature would be ascribed not to where it occurred but to where the species has been sampled most frequently. Finally, if the authors want to use the figure at all, they also must explain what exactly the shift in habitat temperature is. In their rebuttal they write it is change in abiotic temperature. Temperature where? At the inferred position of the optimum of one of the inferred habitat optima? Average throughout the entire niche? Global average?

3) A closer look at Figure 1 made me wonder how well the model actually reflects past temperatures. The figure indicates that there were no places in the glacial ocean with temperatures reaching 30°C like they do today. This seems even stronger in the cGENIE model compared to the LGM data. Is this realistic? Is there any evidence showing that no part of the glacial ocean was warmer than 25°C as suggested by the cGENIE run? To my knowledge, this is simply not true. The glacial ocean was on average cooler because of the contraction of the warmer water bodies, but not because glacial tropics were so much colder everywhere. Or maybe I am reading the figure wrongly? But then, why does the blue line always trail so much behind the green line?

Minor comments:
Usage of “threatens” in abstract and first paragraph is inappropriate. The plankton is affected, we are threatened by the consequences.

For the discussion on Biomass, please note that biomass is not the same as production. Production is the product of standing stock and turnover time. For ecosystem function, biomass is irrelevant, production is everything. Perhaps one can assign to each ecogroup an estimate of turnover time and see if it has any effect on the resulting production? Or, and this would be a leap of faith, the authors would have to

state that they consider turnover rates to be the same across all taxa.

Line 97: the contraction could not have been into the Arctic but towards the high latitudes (it must have occurred on both hemispheres but not reach polar waters where the quoted optimal temperatures are never realised).

Line 133: I believe that the authors should include more reasoning here on why they interpret the results as indicating acclimation – how is the conclusion on acclimation exactly derived from the observed pattern?

Line 191: write: in the past, it is possible to explore if acclimation to future warming will allow foraminifera to maintain their biomass (and thus their ecosystem function)

Line 225: better formulation: Our simulations indicate that even when the possibility of acclimation is accounted for in the model, planktonic foraminifera will not be able to track the pace of future warming …

Line 237: Begin by: Overall, the global biomass…

Author Rebuttals to Second Revision:

Response to referee

Referee #1 (Remarks to the Author):

I would like the authors to consider the following comments:

1) The response to my first two comments (lack of adaptation and relative vs absolute abundance) needs to be incorporated into the paper.

[Response] These two points are indeed highly relevant to our readers. In the revised manuscript, these two points are clearly demonstrated in two paragraphs (Lines 132-143 for the first point and Lines 119-122 for the second point) based on previous responses.

The argument presented with Figure R1 is wrong. I have included below an example that illustrates this. Red and blue are hypothetical absolute abundance patterns of two species, grey is the relative abundance of the blue species. This reverses because the thermal niche of the red species is narrower.

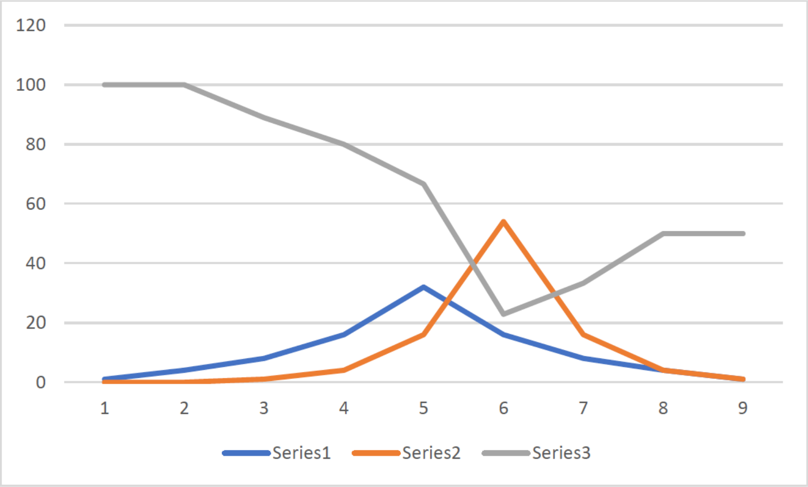

[Response] We appreciate the reviewer's example figure which leads to further in-depth thinking. The reviewer's figure contains two extreme scenarios where the bias of relative abundance method is largest:

1) a species has its lowest absolute abundance but the highest relative abundance (corresponding to the two tails in the figure)

This happens when a few species coexist and each of them has low absolute abundance (i.e., low species richness and total abundance). Such scenario is very rare for foraminifera study because foraminiferal diversity is lowest in high latitude where abundance is highest.

2) a species has its highest absolute abundance but the lowest relative abundance (corresponding to the blue species in 5-6 °C in the figure)

This happens for a rare species with a similar optimal niche to a dominant species. And such scenario only influences the rare species, not the dominant species. For example, in the reviewer's figure, the relative and absolute optimal condition of the orange species are always 6 °C. This therefore confirms that our estimation of dominant species and hence ecogroup results are robust.

Combing the two scenarios, we can conclude that the risk of estimation uncertainty is highest for rare species, whereas dominant species are hardly influenced. We add a paragraph in method section (Lines 575-582) to highlight such uneven uncertainties.

2) I would indeed urge the authors to move the presence-absence data analysis into the supplement. There are too many problems associated with it.

[Response] We agree that this section might distract from the main points of the paper. In the revised manuscript, we moved it into the supplementary information with a brief reference in the main text (Line 126).

Specifically, we removed the analysis of the 0-700 ka data (including Figure 2) as it inherently might cover both acclimation or adaptation given our knowledge of genetic changes in cryptic taxa. Additionally, the sampling noise in the long-term data result in unrealistic results as pointed out by the reviewer. This is now stated in the supplementary information (Lines 206-212).

We still kept the LGM/PI subset of this data analysis to support our result based on absolute abundance, as requested by one of our previous reviewers.

3) A closer look at Figure 1 made me wonder how well the model actually reflects past temperatures. The figure indicates that there were no places in the glacial ocean with temperatures reaching 30°C like they do today. This seems even stronger in the cGENIE model compared to the LGM data. Is this realistic? Is there any evidence showing that no part of the glacial ocean was warmer than 25°C as suggested by the cGENIE run? To my knowledge, this is simply not true. The glacial ocean was on average cooler because of the contraction of the warmer water bodies, but not because glacial tropics were so much colder everywhere. Or maybe I am reading the figure wrongly? But then, why does the blue line always trail so much behind the green line?

[Response] This cGENIE model configuration indeed overestimates the cooling in the LGM tropics, and it is our long-term goal to develop the model and better represent paleoclimate details. However, a tropical temperature bias here is not critical to our results, as it does not to impact the general agreement between model and data in both climate and foraminifera niche.

Minor comments:
Usage of "threatens" in abstract and first paragraph is inappropriate. The plankton is affected, we are threatened by the consequences.

[Response] This is changed to "impacts".

For the discussion on Biomass, please note that biomass is not the same as production. Production is the product of standing stock and turnover time. For ecosystem function, biomass is irrelevant, production is everything. Perhaps one can assign to each ecogroup an estimate of turnover time and see if it has any effect on the resulting production? Or, and this would be a leap of faith, the authors would have to state that they consider turnover rates to be the same across all taxa.

[Response] The biomass in the manuscript exclusively means biomass stock (or standing stock) rather than biomass production. However, we can directly calculate the biomass source term in the model (carbon uptake flux) to represent biomass production rate (Fig. R1). We find this trend is consistent with standing stock result (extended data fig. 6). We now clarified this in Line 179 and Fig. 2 caption (Line 415).

Using this biomass standing stock and production rate, we calculate the turnover time for each foram group. This result is 25 d for symbiont-barren non-spinose foram, 17 d for symbiont-barren spinose foram, 15/50 d for symbiont-obligate group depending on whether including the carbon flux from symbionts. Therefore, they are not same across groups. The estimated turnover times are generally consistent with foraminifera's reproduction period which mostly follows synodic lunar and semi-lunar cycle.

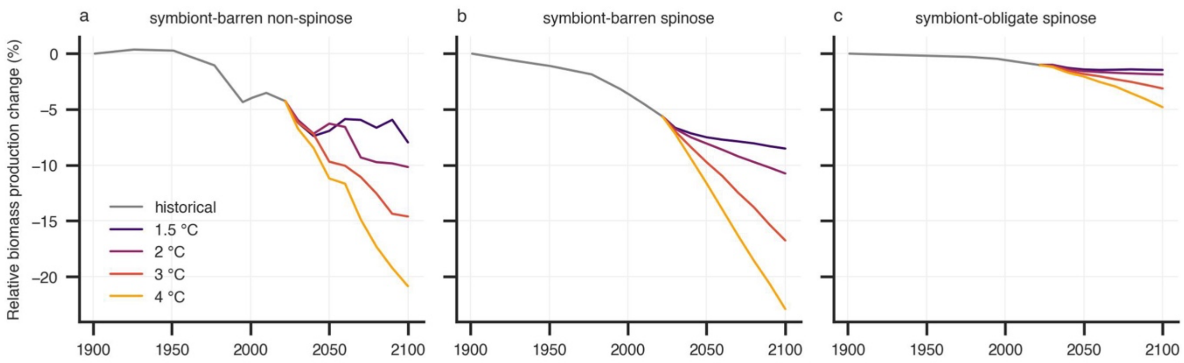

Fig. R1. The trend of simulated biomass production rate (i.e., carbon uptake fluxes in the model) under warming for each foraminifera group. The overall trend is consistent with the biomass standing stock (extended data fig. 6).

Line 97: the contraction could not have been into the Arctic but towards the high latitudes (it must have occurred on both hemispheres but not reach polar waters where the quoted optimal temperatures are never realised).

[Response] The "Arctic" is changed to "high latitudes".

Line 133: I believe that the authors should include more reasoning here on why they interpret the results as indicating acclimation – how is the conclusion on acclimation exactly derived from the observed pattern?

[Response] This is resolved following our response to the main comment #1.

Line 191: write: in the past, it is possible to explore if acclimation to future warming will allow foraminifera to maintain their biomass (and thus their ecosystem function)
Line 225: better formulation: Our simulations indicate that even when the possibility of acclimation is accounted for in the model, planktonic foraminifera will not be able to track the pace of future warming ...
Line 237: Begin by: Overall, the global biomass...

[Response] These suggestions are implemented.

Reviewer Reports on the Third Revision:

Referees' comments:

Referee #1 (Remarks to the Author):

I am pleased to see that the authors were able to incorporate all my previous comments and from my point of view, the manuscript now appears suitable for publication.

Author Rebuttals to Third Revision:

Referees' comments:

Referee #1 (Remarks to the Author):

I am pleased to see that the authors were able to incorporate all my previous comments and from my point of view, the manuscript now appears suitable for publication.

[Response] We sincerely thank the referee for helping to improve the manuscript.
